# Evolution of a laser wake cavity in a MCF plasma

**DOI:** 10.1038/s41598-024-77739-2

**Published:** 2024-11-13

**Authors:** Andreas Bierwage, Timur Zh. Esirkepov, James K. Koga, Alexander S. Pirozhkov, Nobuyuki Aiba, Kai Huang, Masaki Kando, Hiromitsu Kiriyama, Akinobu Matsuyama, Kouji Shinohara, Masatoshi Yagi, Gunsu S. Yun

**Affiliations:** 1 National Institutes for Quantum Science and Technology (QST), Rokkasho Institute for Fusion Energy, Rokkasho, Aomori 039-3212 Japan; 2 QST, Naka Institute for Fusion Science and Technology, Naka, Ibaraki 311-0193 Japan; 3 QST, Kansai Institute for Photon Science (KPSI), Kizugawa, Kyoto 619-0215 Japan; 4 Graduate School of Energy Science, Kyoto University, Uji, 611-0011 Japan; 5 Department of Complexity Science and Engineering, The University of Tokyo, Kashiwa, Chiba 277-8561 Japan; 6 Department of Physics, Pohang University of Science and Technology (POSTECH), Pohang, Gyeongbuk 37673 Republic of Korea; 7 Division of Advanced Nuclear Engineering, POSTECH, Pohang, Gyeongbuk 37673 Republic of Korea

**Keywords:** Magnetically confined plasmas, Nuclear fusion and fission, Nonlinear phenomena

## Abstract

A laser pulse focused to relativistic intensity inside a magnetically confined fusion (MCF) plasma plows away all electrons in its path. The ensuing Coulomb explosion of the ions leaves behind a cavity of microscopic size, with gradients in the electric potential and plasma density orders of magnitude stronger than anything the plasma could generate spontaneously. When posing questions concerning the practical utility of such an exotic perturbation, the life time and structural evolution of the cavity are of interest. Our simulations in a simplified 1D + 2D setting and otherwise realistic parameters suggest that a sub-mm wide seed cavity (meant to resemble the laser wake channel) collapses or disintegrates within 10 ns. The dynamics are sensitive to the relative scales of the cavity, Debye shielding and gyration. We find evidence for the possibility that the collapsing seed cavity spawns solitary micro-cavities. It remains to be seen whether such structures form and survive long enough in a 3D setting to alter the local plasma conditions (e.g., as micro-cavity clusters) in ways that may be utilized for practical purposes such as plasma initiation, diagnostics or control.

## Introduction

A magnetically confined fusion (MCF) plasma as envisioned for ITER experiments can be viewed as an open energy conversion and conveyance system: its state is determined by multi-scale dynamics that move energy and particles between the plasma core and its surface^1,2^. Transport processes in MCF plasmas are most commonly analyzed in terms of the (in)stability of normal eigenmodes or fully developed turbulence around a given reference state. Sub-threshold deformation (metastability) is also being studied^3^. However, there are also nonnormal transients, which are less amenable to systematic analysis and generalization, in part due to a higher sensitivity to initial conditions, including the form of a perturbation. For the hydrodynamic limit, nonmodal stability theory has emerged as a means for analyzing such processes^4^, where “the superposition of decaying nonorthogonal eigenfunctions can produce, in the short term, growth in the norm of a perturbation.” When these transients are sufficiently large to reach the nonlinear regime, they may launch solitary waves or cause long-lasting changes in the system’s structure. Based on such considerations and motivated by the need for precise, efficient and minimally invasive techniques to monitor and control MCF plasmas, one may pose the following question: *What is the fate and life time of microscopically small but strong perturbations that form after a relativistically intense laser pulse passes through such a medium?*

High power lasers and well-diagnosed tokamaks are expensive, large devices. To our knowledge there is currently no laboratory that has both systems operating side-by-side to be combined for an experimental tests dedicated to the above question and the ideas it spurs, some concrete examples of which are discussed in Supplementary Sec. 1. At present, we rely on scaled laser experiments and numerical simulations for feasibility tests. A preliminary report of our results was presented in 2019 in the form of a short conference paper^5^. The present article and the accompanying supplementary material contains more comprehensive analyses and discussions of the results that we have accumulated so far.

Modern laser systems can—depending on their wavelength $\lambda _{\textrm{las}} = 2\pi c/\omega _{\textrm{las}}$—produce terawatt-picosecond to petawatt-femtosecond pulses. Here, $c = 1/\sqrt{\epsilon _0\mu _0}$ is the speed of light in vacuum with permittivity $\epsilon _0$ and permeability $\mu _0$. When focused into a sub-millimeter spot, the laser’s electromagnetic field, ${\varvec{E}}_{\textrm{las}} = -{\varvec{\nabla }}\Phi _{\textrm{las}} - \partial _t{\varvec{A}}_{\textrm{las}}$ and ${\varvec{B}}_{\textrm{las}} = {\varvec{\nabla }}\times {\varvec{A}}_{\textrm{las}}$, can reach or exceed relativistic intensities, which means that an electron with charge $q_{\textrm{e}} = -e$ and rest mass $m_{\textrm{e}}$ is accelerated to relativistic speed within half a wave cycle. When expressed in terms of the normalized amplitude

1 $$\begin{aligned} a_0 = \left| \frac{eA_{\textrm{las}}}{m_{\textrm{e}} c}\right| = \left| \frac{e E_{\textrm{las}}}{m_{\textrm{e}}\omega _{\textrm{las}} c}\right| = \left| \frac{e E_{\textrm{las}}\lambda _{\textrm{las}}}{2\pi m_{\textrm{e}} c^2}\right| = \frac{|E_{\textrm{las}}|}{E_1} = \sqrt{\frac{I_{\textrm{las}}}{I_1}}, \quad \mathrm{with\; intensity} \quad I_1 = \frac{|E_1|^2}{2\mu _0 c} = \frac{1.37\times 10^{18}\,\textrm{W}/\textrm{cm}^{2}}{(\lambda _{\textrm{las}}\,[\mu \textrm{m}])^2}, \end{aligned}$$

the relativistic regime is $a_0 \gtrsim 1$ (for more information, see Section III A. of Ref.^6^ and the introduction of Ref.^7^). Such conditions can be routinely achieved with laser wavelengths around $1\,\mu \textrm{m}$ (e.g., solid-state Ti:sapphire lasers) and perhaps with some effort using $10\,\mu \textrm{m}$ $\textrm{CO}_2$ gas lasers^8,9^ (see Supplementary Sec. 1.4). In either case, the critical density

2 $$\begin{aligned} n_{\textrm{crit}} = \frac{\pi }{r_{\textrm{e}} \lambda _{\textrm{las}}^2} \approx 10^{27}\,\textrm{m}^{-3}\left( \frac{1\,\mu \textrm{m}}{\lambda _{\textrm{las}}}\right) ^2, \quad \text {with the classical electron radius} \quad r_{\textrm{e}} \equiv \frac{1}{4\pi \epsilon _0}\frac{e^2}{m_{\textrm{e}}c^2}, \end{aligned}$$

is many orders of magnitude above the (line-averaged) electron density limit $n_{\textrm{e}} \lesssim 10^{20}\,\textrm{m}^{-3}$ of a tokamak plasma,^10^ so we have $n_{\textrm{e}}/n_{\textrm{crit}} = \omega _{\textrm{pe}}^2/\omega _{\textrm{las}}^2 \ll 1$, where $\omega _{\textrm{las}}/(2\pi ) \sim 100\,\textrm{THz}$, and the Langmuir frequency for cold electron plasma waves is

3 $$\begin{aligned} \omega _{\textrm{pe}} = \left( \frac{n_{\textrm{e}}e^2}{\epsilon _0 m_{\textrm{e}}}\right) ^{1/2} \approx 2\pi \times 90\,\textrm{GHz} \left( \frac{n_{\textrm{e}}}{10^{20}\,\textrm{m}^{-3}}\right) ^{1/2}. \end{aligned}$$

**Fig. 1:**
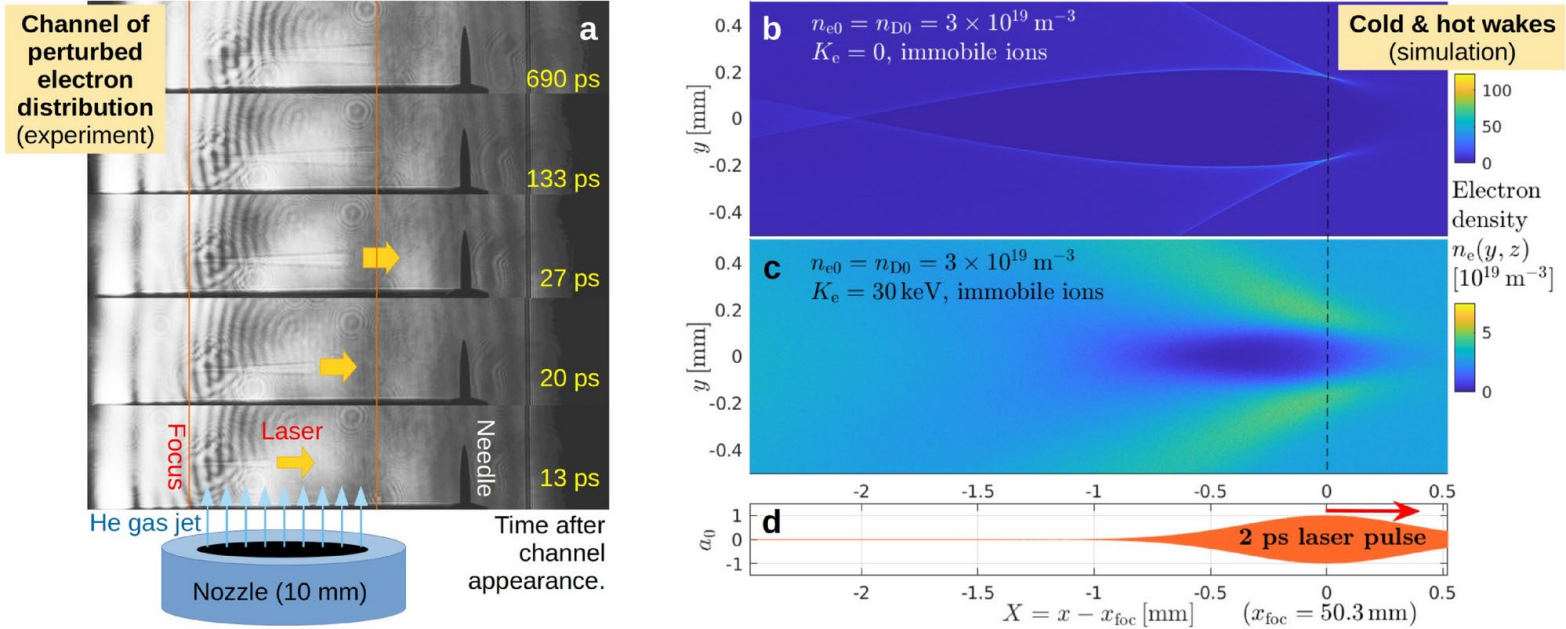
Perturbations observed behind relativistically intense laser pulses. Column (**a**) shows a sequence (bottom to top) of shadowgraphic images taken in our experiments with the J-KAREN-P laser using a low-pressure helium gas jet with an estimated electron density of $n_{\textrm{e}}/n_{\textrm{crit}} \sim 10^{-4} \cdots 10^{-3}$. The images were taken during different shots with different delays of the optical probe pulse and show the formation of a conical plasma channel and its sustainment for at least $0.7\,\textrm{ns}$. The high-contrast $35\,\textrm{fs}$ laser pulse was focused near the entrance to the gas jet (here on the left-hand side), where it was estimated to have reached a super-relativistic intensity, with $a_0 \sim 4.7$. Further details can be found in Supplementary Sec. 1.3 Panels (**b**) and (**c**) each show one snapshot of the electron density field $n_{\textrm{e}}(x,y)$ in the immediate vicinity of a relativistic laser pulse (panel (**d**)) in 2D PIC simulations initialized with cold and hot electrons, respectively. The values of the ambient density $n_{\textrm{e}} = n_{\textrm{D}} = 3\times 10^{19}\,\textrm{m}^{-3}$ in both cases and the electron temperature $K_{\textrm{e}} = 30\,\textrm{keV}$ in (**c**) represent MCF reactor-relevant conditions. These results illustrate the effect of thermal motion on an unmagnetized electron density wake. The simulations were performed with the relativistic PIC code EPOCH^11^ using a moving window that follows a $2\,\textrm{ps}$ laser pulse with wavelength $\lambda _{\textrm{las}} = 4\,\upmu \textrm{m}$ as in Fig. 3 of Ref. ^12^ (with the numerical parameters of Fig. 8a of Ref. ^12^). A quantitative comparison between laser pulses propagating through (**b**) cold and (**c**) hot plasma is presented in Supplementary Sec. 1.5.

This means that a (cold) tokamak plasma is effectively transparent to such lasers in the sense that their light passes through it nearly unaltered. The intense laser pulse does, however, leave behind a trail that can be seen in shadowgraphic images like those in Fig. 1a taken at the J-KAREN-P facility^13–15^. Here, a high-contrast $35\,\textrm{fs}$ laser pulse was focused to $a_0 \sim 4.7$ near its point of entrance to a supersonic helium gas jet. The estimated electron density $10^{23} \cdots 10^{24}\,\textrm{m}^{-3}$ was high enough for the laser plasma channel to be visible in the shadowgraphs, and low enough to count as being effectively transparent. (Further details and discussions of possible implications of these observations based on scaling arguments can be found in Supplementary Sec. 1.3.)

Such a low-density plasma also remains transparent when the electrons are heated to a reactor-relevant “temperature” (thermal energy) $K_{\textrm{e}} = m_{\textrm{e}}v_{\textrm{th,e}}^2/2$ on the order of $10\,\textrm{keV}$, in spite of the fact that the corresponding thermal velocity $v_{\textrm{th,e}}/c \sim 0.2$ becomes a significant fraction of the speed of light *c* (see Supplementary Sec. 1.5). Hence, we shall assume in the following that the laser passes through a nearly transparent plasma, so that soliton formation based on laser light trapping^16–19^ will not occur.

The laser will, however, plow all electrons out of its path whenever its amplitude reaches $a_0 \gtrsim 1$. For illustration, Fig. 1b,c show a comparison of the relativistic laser wake and bow waves in an unmagnetized cold and hot plasma, respectively. One can see that the fold singularity in the relativistic bow wave^20,21^ reaches $\textrm{max}|n_{\textrm{e}}|/n_{\textrm{e0}} \approx 120/3 = 40$ for $K_{\textrm{e}} = 0$ in Fig. 1b, whereas it is subject to strong thermal broadening for $K_{\textrm{e}} = 30\,\textrm{keV}$ in Fig. 1c, such that $n_{\textrm{e}}/n_{\textrm{e0}} \lesssim 2$ everywhere.

**Fig. 2:**
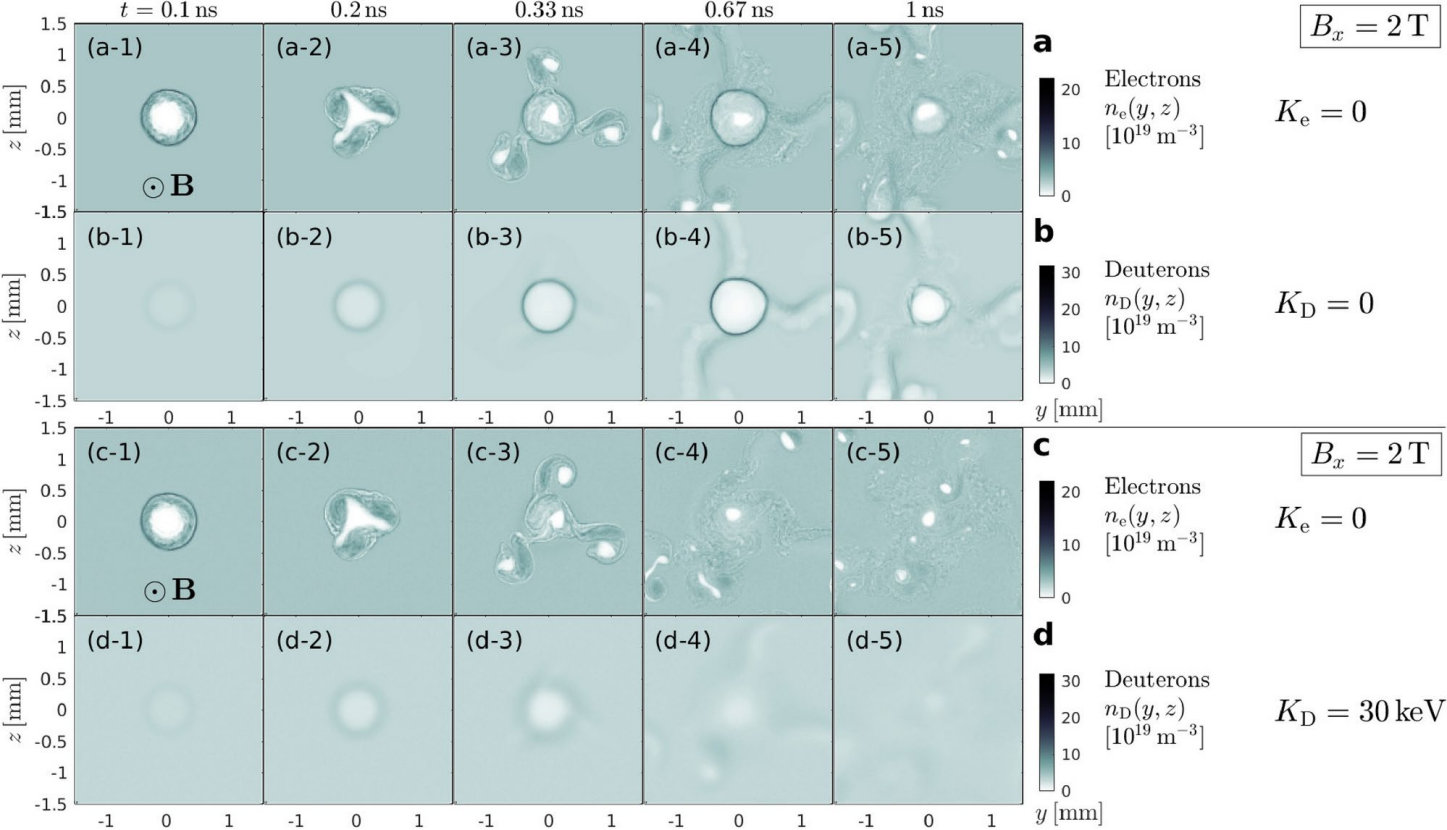
2D PIC simulations illustrating the effect of thermal motion on the Coulomb explosion of deuterons located inside a magnetized electron cavity. Rows (**a**) and (**b**) show the evolution of the density fields $n_\alpha (y,z)$ of electrons ($\alpha =\textrm{e}$) and deuterons ($\alpha =\textrm{D}$) initialized with zero velocity ($K_\alpha =0$), and rows (**c**) and (**d**) show results for a case with cold electrons and hot Maxwellian deuterons ($K_{\textrm{e}} = 0$, $K_{\textrm{D}} = 30\,\textrm{keV}$). These simulations of a stationary quadratic slab representing the *y*–*z* plane transverse to the laser’s path were performed using the code EPOCH^11^. They start from a circular hole in the electron density that is meant to approximately resemble the transverse cross-section of the magnetized laser wake channel. The 2 tesla strong ambient magnetic field vector ${\varvec{B}}$ points out of the plane. The number of particles per cell (ppc) was $N_{\textrm{ppc}} = 1$ for each cold species and $N_{\textrm{ppc}} = 40$ for the hot Maxwellian deuterons. Similar results were obtained with $N_{\textrm{ppc}} = 10$ and 100, and with different simulation box sizes and spatial resolutions. Further details concerning the numerical setup of and dynamics in the cold deuteron case (**d**) can be found in Supplementary Fig. 9.

In either case, the laser (Fig. 1d) leaves behind a deep cavity in the electron density. The multi-tesla strength $B = |{\varvec{B}}|$ of the ambient magnetic field in a MCF plasma hinders the electrons’ return, giving the ions additional time to respond via a Coulomb explosion^22,23^. The result is a microscopically small but deep cavity in the plasma density^12^. The qualitative features of the Coulomb explosion are preserved when the ions are initialized with a hot Maxwellian velocity distribution, as the simplified example in Fig. 2 illustrates for deuterons (D) with initial temperatures of $K_{\textrm{D}} = 0$ (top) and $30\,\textrm{keV}$ (bottom). One readily observable difference is that the spatial structures appear smoother and the seed cavity vanishes more quickly in the hot ion case. Meanwhile, in both cases, the collapsing seed cavity leaves behind micro-cavities in the electron density.

The examples in Fig. 2 already show where the trend goes: in a hot plasma, the original cavity is unlikely to remain intact for much longer than a few nanoseconds. What remains is a turbulent field that may contain fragments of the seed cavity in the form of solitary structures, which are at least partly nonneutral and could be long-lived.

In the present paper, we use the relativistic particle-in-cell (PIC) codes REMP^24^ and EPOCH^11^ to investigate in more detail the process shown in Fig. 2. Parameter scans in a realistically achievable range of values are performed to develop our physical intuition for the unusual combination of a tightly-focused relativistic laser pulse and a hot sparse MCF plasma. The purpose of this paper is to motivate further study on porous plasma states like that seen in the final snapshots of Fig. 2 and examine whether such exotic perturbations could be used to respond to the urgent need for better MCF plasma control techniques, which originally motivated this study and is discussed in Supplementary Sec. 1.

A realistic 3D simulation of the cavity’s evolution on the $10\,\textrm{ns}$ time scale and beyond would be computationally challenging. Even with periodic boundary conditions in the transverse direction, the simulation box should be at least $10\,\textrm{mm}$ wide to rule out spurious interactions of gyrating deuterons with the cavity and its perturbed surroundings. The requirements for the box size in the longitudinal direction are even more severe. The cavity length *L* depends on the Rayleigh length of the focused beam and the thermal electron velocity. For example, a laser with wavelength $\lambda _{\textrm{las}} \sim 1\,\upmu \textrm{m}$ and focal width of about $0.18\,\textrm{mm}$ has a Rayleigh length of about $100\,\textrm{mm}$ (cf., Fig. 1 of Ref.^12^). One also needs to add some buffer zone for the cavity to expand into^12^. That zone contains weakly perturbed plasma, where the electrons are left largely unperturbed by the laser pulse. They will then respond to the cavity’s presence, while also performing free-streaming motion along ${\varvec{B}}$.

**Fig. 3:**
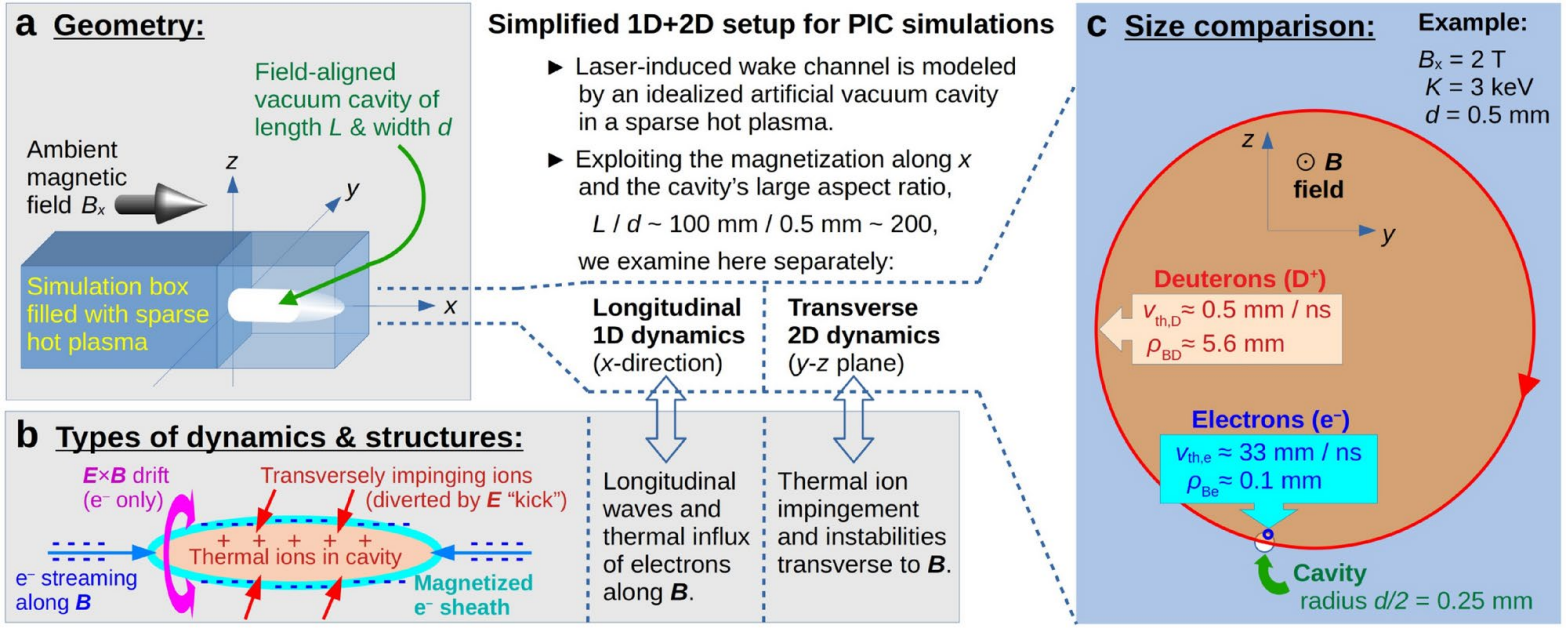
(**a**) Geometry of the simulated scenario, (**b**) types of dynamics and structures, and (**c**) a comparison between the sizes of the laser-induced vacuum cavity (white circle) and the gyroorbits of electrons (blue) and deuterons (red). In this work, we use a simplified setup where we simulate separately the transverse (2D) and unmagnetized longitudinal (1D) dynamics, exploiting the large aspect ratio $L/d \gtrsim 100$ of the cavity and the magnetization due to an ambient ${\varvec{B}}$ field along the *x*-direction.

Before engaging in such expensive 3D simulations, which we leave for future work, it is meaningful to examine the cavity’s evolution and life time in a hot plasma using a simplified setup. The approach chosen here is to consider separately the longitudinal (1D, unmagnetized) and transverse (2D, magnetized) dynamics as outlined in the center of Fig. 3.

As a working example, we consider a field-aligned laser wake channel as illustrated in Fig. 3a. The laser has propagated along the *x* axis, which is also the direction of the ambient magnetic field, here assumed to have a strength of $B_{\textrm{x}} = 2\,\textrm{T}$. The ambient plasma is assumed to consist of electrons ($\textrm{e}^-$) and deuterons ($\textrm{D}^+$) that have an isotropic Maxwellian distribution with a thermal energy of $K_{\textrm{e}} = K_{\textrm{D}} = 3\,\textrm{keV}$. The laser is assumed to have left behind a vacuum cavity with a diameter of $d = 0.5\,\textrm{mm}$, which corresponds to five times the thermal electron gyroradius $\rho _{\textrm{Be}} \equiv v_{\textrm{th,e}}/|\omega _{\textrm{Be}}| \sim 0.1\,\textrm{mm}$ and is less than a tenth of the thermal deuteron gyroradius $\rho _{\textrm{BD}} \equiv v_{\textrm{th,D}}/\omega _{\textrm{BD}} \sim 5.6\,\textrm{mm}$. Here, the gyrofrequency is

4 $$\begin{aligned} \omega _{\textrm{B}\alpha } = \frac{q_\alpha B}{m_\alpha } \quad \Rightarrow \quad \omega _{\mathrm{B\alpha }} \approx 2\pi B[\textrm{T}]\times \left\{ \begin{array}{lcl} 28\,\textrm{GHz} & : & \alpha = \textrm{e}, \\ 7.6\,\textrm{MHz} & : & \alpha = \textrm{D}.\end{array}\right. \end{aligned}$$

For illustration, Fig. 3c shows the relative proportions of *d*, $\rho _{\textrm{Be}}$ and $\rho _{\textrm{BD}}$ for this realistic example. While the above parameter values are based on present-day tokamak experiments, the situation will be similar in ITER and DEMO, where the values of *B* and *K* will both be larger, so that $\rho _{\textrm{B}\alpha }$ will have sizes comparable to those in our working example. Thus, with the laser energies and magnetic field strengths available today and in the foreseeable future, the thermal ion gyroradius is always going to be much larger than the cavity diameter, $\rho _{\textrm{BD}} \gg d$, which means that the cavity is topologically connected to a large reservoir of ions that are going to impinge on it within a matter of nanoseconds, as illustrated by the red arrows in Fig. 3b.

Other key components of the particle dynamics and cavity structure are also shown in Fig. 3b for our example: Thermal electron motion with characteristic velocity $v_{\textrm{th,e}} \sim 33\,\mathrm{mm/ns}$ produces a double layer (sheath) around the cavity boundary that forms on the picosecond time scale due to both longitudinal streaming along, and gyration across ${\varvec{B}}$. The electrons’ longitudinal streaming will be constrained to the ions’ motion on scales greater than the Debye length (here with $\sum _\alpha q_\alpha ^2 n_\alpha /K_\alpha \approx 2 e^2 n_{\textrm{e}}/K_{\textrm{e}}$)

5 $$\begin{aligned} \lambda _{\textrm{D}} \approx \frac{\lambda _{\textrm{De}}}{\sqrt{2}} = \frac{1}{\omega _{\textrm{pe}}}\left( \frac{K_{\textrm{e}}}{2 m_{\textrm{e}}}\right) ^{1/2} = \left( \frac{\epsilon _0 K_{\textrm{e}}}{2n_{\textrm{e}} e^2}\right) ^{1/2} = 16.6\,\mu \textrm{m} \times \left( \frac{n_{\textrm{e}}}{10^{20}\,\textrm{m}^{-3}}\right) ^{-1/2}\left( \frac{K_{\textrm{e}}}{1\,\textrm{keV}}\right) ^{1/2}. \end{aligned}$$

In the plane transverse to ${\varvec{B}}$, the initial sheath width will be on the order of the electron gyroradius $\rho _{\textrm{Be}}$, but it will be broadened by $\lambda _{\textrm{D}}$ as soon as it is overrun by the ions with their much greater $\rho _{\textrm{BD}}$. In fact, in our example with $K_{\textrm{e}} = 3\,\textrm{keV}$, and for the density range $n_{\textrm{e}} \sim (0.3 \cdots 30)\times 10^{19}\,\textrm{m}^{-3}$ that is of interest for tokamak plasmas (from edge to core), the Debye length $\lambda _{\textrm{D}} \sim (0.2 \cdots 0.02)\,\textrm{mm}$ can be larger or smaller than our $\rho _{\textrm{Be}} \sim 0.1\,\textrm{mm}$, and $\lambda _{\textrm{D}}$ can even be comparable to the diameter $d = 0.5\,\textrm{mm}$ of our seed cavity. We thus expect the cavity’s transverse dynamics to vary noticeably across the density range of interest, which will be confirmed by simulations below.

The electrons will also perform drift motion around the cavity axis due to the local electric field. We will later see that the flow direction will vary, so the direction of the magenta arrow in Fig. 3b has been drawn bidirectionally. Notice that, unlike in magnetohydrodynamics (MHD), the charged particles in the present case do not all drift with the same speed ${\varvec{v}}_{\textrm{E}} = {\varvec{E}}\times {\varvec{B}}/B^2$, because our electric field varies on a length scale $\Delta _E \sim \lambda _{\textrm{D}}$ satisfying

6 $$\begin{aligned} \rho _{\textrm{Be}} \lesssim \Delta _E \lesssim d \ll \rho _{\textrm{Bi}}. \end{aligned}$$

Only the electrons can be treated as a magnetized species on that scale. One consequence of Eq. (6) is that an effective magnetization current ${\varvec{j}}_{\textrm{mag}}$ and an associated magnetic perturbation $\delta {\varvec{B}}$ will arise only from gradients in the energy density (pressure) of electrons. The second consequence is that only the electron’s electric drift velocity may be assumed to be close to ${\varvec{v}}_{\textrm{E}}$ of the MHD limit (with some attenuation), whereas the ions will be subject to transient acceleration (a kick) along ${\varvec{E}}$ when they pass the cavity’s domain. One may then anticipate that the ions can be deflected before entering the cavity if the ratio

7 $$\begin{aligned} |{\varvec{E}}|/|{\varvec{u}}\times {\varvec{B}}| \sim v_{\textrm{E}}/v_{\textrm{th,i}} \end{aligned}$$

exceeds unity. We will later examine the relevance of this mechanism by measuring the value of $v_{\textrm{E}}/v_{\textrm{th,i}}$ in the simulations.

## Results

### Longitudinal dynamics (1D, unmagnetized): excitation of solitary micro-cavity waves (Fig. 4)

**Fig. 4:**
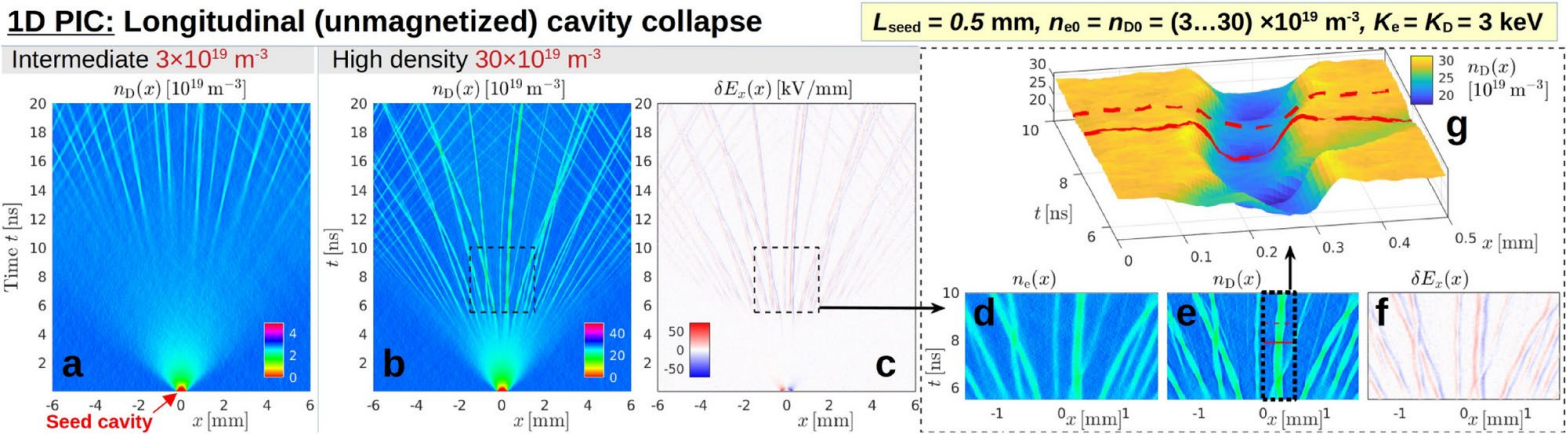
EPOCH simulation of the thermal collapse of a 1D cavity surrounded by a $3\,\textrm{keV}$ deuterium plasma with intermediate (**a**) and high density (**b**)–(**g**). The initial state is a $12\,\textrm{mm}$ long neutral plasma with periodic boundaries. A seed cavity of length $L_{\textrm{seed}} = 0.5\,\textrm{mm}$ with sharp boundaries is located around $x=0$. This simplified setup is meant to represent a laser-induced vacuum cavity (after a Coulomb explosion) and to capture aspects of the plasma dynamics along an ambient magnetic field. The colored contour plots in panels (**a**) and (**b**) show the evolution of the deuteron density profile $n_{\textrm{D}}(x)$ for $n_0 = 3\times 10^{19}\,\textrm{m}^{-3}$ and $30\times 10^{19}\,\textrm{m}^{-3},$ respectively. For the high-density case, panel (**c**) shows the electric perturbation $\delta E_x(x)$. The data in the dashed rectangles are enlarged in panels (**d**)–(**f**), including also a plot of the electron density $n_{\textrm{e}}(x)$. The density landscape of a solitary cavity from panel (**e**) is shown in more detail in panel (**g**), with solid and dashed red lines drawn for orientation at $t = 7.9\,\textrm{ns}$ and $8.7\,\textrm{ns}$, where the cavity has contracted and expanded, respectively. See Supplementary Fig. 7 for additional parameter scans.

A sufficiently violent collapse of the cavity in the unmagnetized (*x*) direction may trigger the formation of solitary structures that, albeit microscopic, could be long-lived and arise in large numbers. This is demonstrated in Fig. 4, where we present results of 1D PIC simulations of such a collapse, starting from a small vacuum cavity. Recall from Fig. 3 that the laser-induced vacuum cavity can easily have a length *L* on the order of $100\,\textrm{mm}$ or more. In the present case, however, we use only a very short seed cavity with $L_{\textrm{seed}} = 0.5\,\textrm{mm}$. The primary motivation for this choice is the need to minimize boundary effects by providing a sufficiently large reservoir of thermal electrons on both sides of the cavity. Here, we choose a box size of $L_x = 12\,\textrm{mm} \gg L_{\textrm{seed}}$ with periodic boundaries, so that the ambient density would drop by at most $4\%$ if the $0.5\,\textrm{mm}$ wide cavity was filled completely. We assume that the ions in the laser wake channel already underwent a Coulomb explosion, so we simply removed all electrons and ions from the cavity at $t=0$. Thus, the initial phase of this simulation consists of the impingement of $3\,\textrm{keV}$ Maxwellian deuterons, which wipe out the ion cavity within a nanosecond as can be seen in Fig. 4a,b. The electrons follow suit within $\pm \lambda _{\textrm{D}}$. The absence of a magnetic field and the small initial size of the cavity means that this 1D setup may also be taken as a proxy for the case where a laser had been shot transversely (or at a steep angle) with respect to an ambient magnetic field.

Shortly after the initial collapse, Fig. 4a shows how the cavity spreads with a speed of about $0.8\,\textrm{mm}/\textrm{ns}$ ($\gtrsim v_{\textrm{th,D}} \approx 0.5\,\mathrm{mm/ns}$) while it is being flooded with particles. At first, the particle distribution appears diffuse. However, after a few nanoseconds, a few dozen solitary structures become visible, which proceed to fan out from $x=0$. In the high-density case in Fig. 4b, the diffuse phase looks shorter. Moreover, the solitary structures appear to interact more strongly, to the point that some of them oscillate around one another in bundles. Such mutual interactions (which are known to occur between multi-dimensional electromagnetic solitons^25^) are one important aspect that distinguishes the present 1D structures from ideal solitons, so we consider them to be solitary waves in a broader sense, meaning: long-lived nonnormal modes, whose structure is determined by a localized self-sustaining strong perturbation of the medium.

A detailed view of the structures and crossings of micro-cavities can be gleaned from panels (**d**)–(**g**) in Fig. 4. The boundaries of these solitary cavity waves appear sharper in the ion density (**e**) than in the electron density (**d**). Their apparent sizes vary and tend to be around $\ell \sim 0.1\,\textrm{mm}$ in the present cases. Although the length scales are comparable, we do not see any obvious correlation with the Debye length, whose values are $\lambda _{\textrm{D}} \approx 0.05\,\textrm{mm}$ and $0.02\,\textrm{mm}$ in the two cases shown in Fig. 4. The electric perturbation $\delta E_x$ in Fig. 4c,f is always positive on the left-, and negative on the right-hand side, both during the initial collapse of the seed cavity and in the solitary stage. This implies that, throughout the simulation, the cavity boundary consists of a double layer (sheath) with more electrons inside the cavity and more ions on the outside. This makes sense because, in the absence of an ambient magnetic field, there is nothing aside from electric forces to constrain the electrons’ streaming into the cavity with their high velocities, $v_{\textrm{th,e}} \gg v_{\textrm{th,D}}$. The electric potential difference at each double layer in the solitary stage is about $\ell |\delta E_x| \sim 2\,\textrm{kV}$, which is comparable to the thermal energy $3\,\textrm{keV}$ of the particles in this simulation.

The particle density inside these solitary structures is about $15 \cdots 20\%$ and $25 \cdots 30\%$ below the respective ambient densities $n_0 = 3\times 10^{19}\,\textrm{m}^{-3}$ and $30\times 10^{19}\,\textrm{m}^{-3}$. While robust on long time scales of at least tens of nanoseconds, these solitary cavities can exhibit pulsations, which are perhaps visible most clearly in the surface plot in Fig. 4g. The density inside a solitary cavity wave decreases upon contraction and increases upon expansion, which seems to imply that the pulsations are due to a repeated flattening and steepening of its wall, during which particles flow all the way across the micro-cavity. The pulsation period seems to be about $3 \cdots 4\,\textrm{ns}$ and $1.5 \cdots 2\,\textrm{ns}$ for $n_0 = 3\times 10^{19}\,\textrm{m}^{-3}$ and $30\times 10^{19}\,\textrm{m}^{-3}$, respectively. Results of additional parameter scans can be found in Supplementary Fig. 7, where it is shown that a longer seed cavity tends to produce larger solitary cavities, and that the number, depth and interaction strength of the solitary structures varies with the temperature ratio, $K_{\textrm{e}}/K_{\textrm{D}}$. A better understanding of the physics and relevance of these observations remains to be established through further study in 3D.

### Transverse dynamics (2D): effect of magnetization, thermal ion influx, and cavity boundary instabilities

**Fig. 5:**
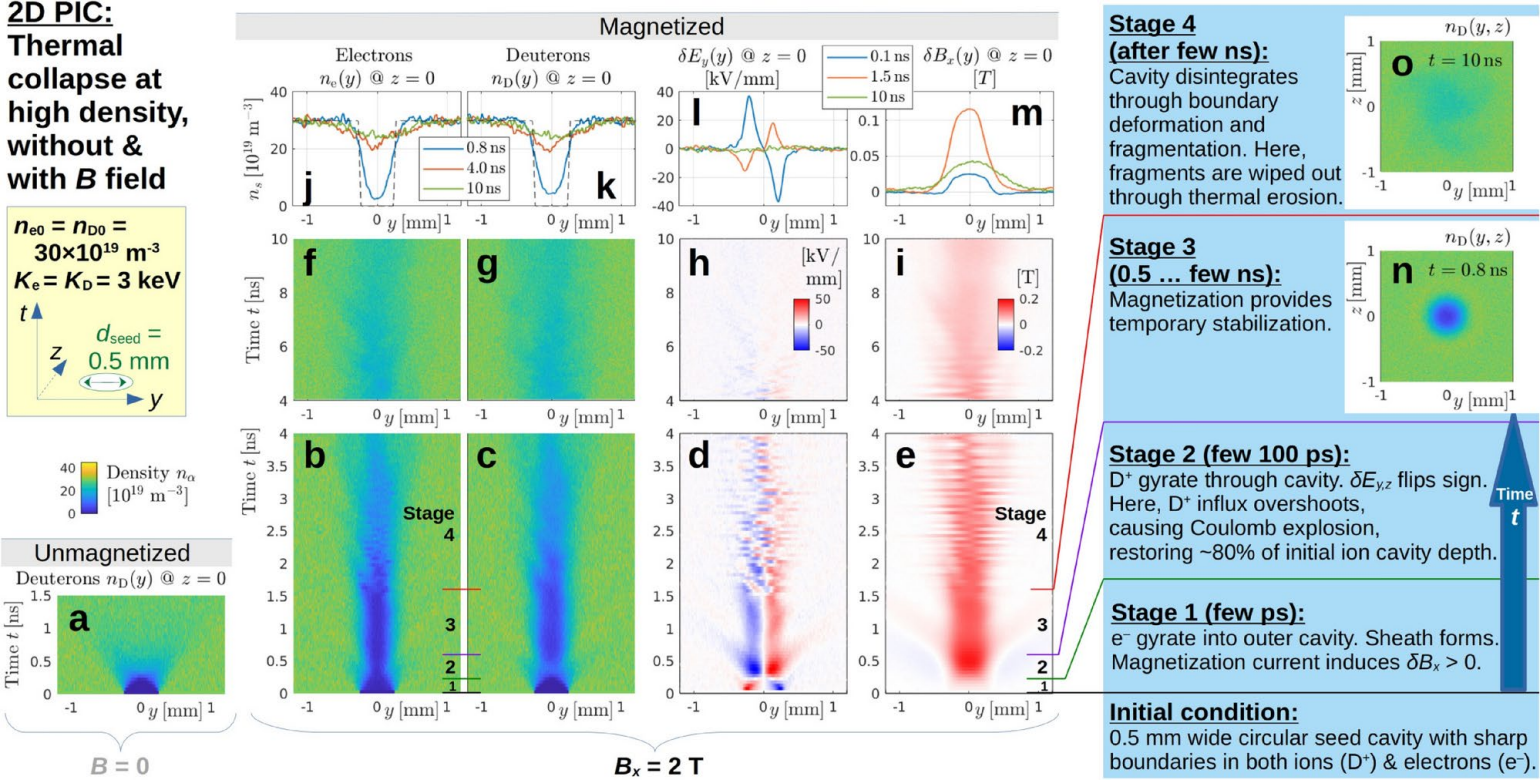
2D REMP simulation of the thermal collapse of a circular seed cavity with diameter $d_{\textrm{seed}} = 0.5\,\textrm{mm}$ in the (*y*, *z*) plane surrounded by a $3\,\textrm{keV}$ hot deuterium plasma, (**a**) without and (**b**)–(**o**) with an ambient magnetic field $B_x = 2\,\textrm{T}$. Panels (**a**)–(**c**) show the temporal evolution of the density profiles $n_\alpha (y)$ at $z = 0$ of deuterons ($\alpha =\textrm{D}$) and electrons ($\alpha =\textrm{e}$) during the first few nanoseconds in the form of colored contour plots. Panels (**d**) and (**e**) show the evolution of the electromagnetic perturbations $\delta E_y(y)$ and $\delta B_x(y)$. Only a small portion of the $12\,\textrm{mm}\times 12\,\textrm{mm}$ wide simulation box is shown. Time *t* advances upward and panels (**f**)–(**i**) show the continuation of the dynamics during the $4 \cdots 10\,\textrm{ns}$ interval. Panels (**j**)–(**m**) show the *y*-profiles in more detail for a few snapshot times, with initial ($t = 0$) density profiles drawn as black dashed lines. We distinguish four stages, labeled 1–4, that are described on the right-hand side of the figure and in more detail in the text. Their transitions are roughly indicated by colored lines in panels (**b**), (**c**) and (**e**). Panels (**n**) and (**o**) show $n_{\textrm{D}}(y,z)$ snapshots taken during stages 3 and 4. Here we used a fairly high density of $30\times 10^{19}\,\textrm{m}^{-3}$. Results of a density scan are presented in Fig. 7.

Figure 5 shows a summary of the main results of our 2D PIC simulations for the dynamics in the (*y*, *z*) plane transverse to the cavity axis *x*. Panel (**a**) shows that, in the absence of an ambient magnetic field ($B = 0$), our circular seed cavity is wiped out by the influx of thermal particles within a few $100\,\textrm{ps}$ (as in the 1D case in Fig. 4). This illustrates the effect of high plasma temperature, which seems to have been negligible in the experiments shown in Fig. 1a. The result suggests that the plasma heating exerted by the prepulse in those laser experiments did not reach the keV-level energies that we are dealing with in the present simulations. Panels (b) and (c) of Fig. 5 show that our axial magnetic field $B_x = 2\,\textrm{T}$ significantly prolongs the cavity’s life time by almost an order of magnitude. We distinguish four stages whose main features are outlined on the right-hand side of Fig. 5 and will be discussed in the following subsections. Different densities and field strengths will also be considered.

#### Stages of a cavity’s thermal collapse at high density in 2D (Fig. 5)

The simulation shown in Fig. 5 was initialized with a $d_{\textrm{seed}} = 0.5\,\textrm{mm}$ wide circular seed cavity that has a sharp boundary, where the density jumps abruptly from zero to its ambient value. A fairly high density of $n_{\textrm{e0}} = n_{\textrm{D0}} = 30\times 10^{19}\,\textrm{m}^{-3}$—as may be found in the central core of a tokamak—is used for both electrons ($\textrm{e}^-$) and ions. Here, the ions are deuterons ($\textrm{D}^+$).

**Fig. 6:**
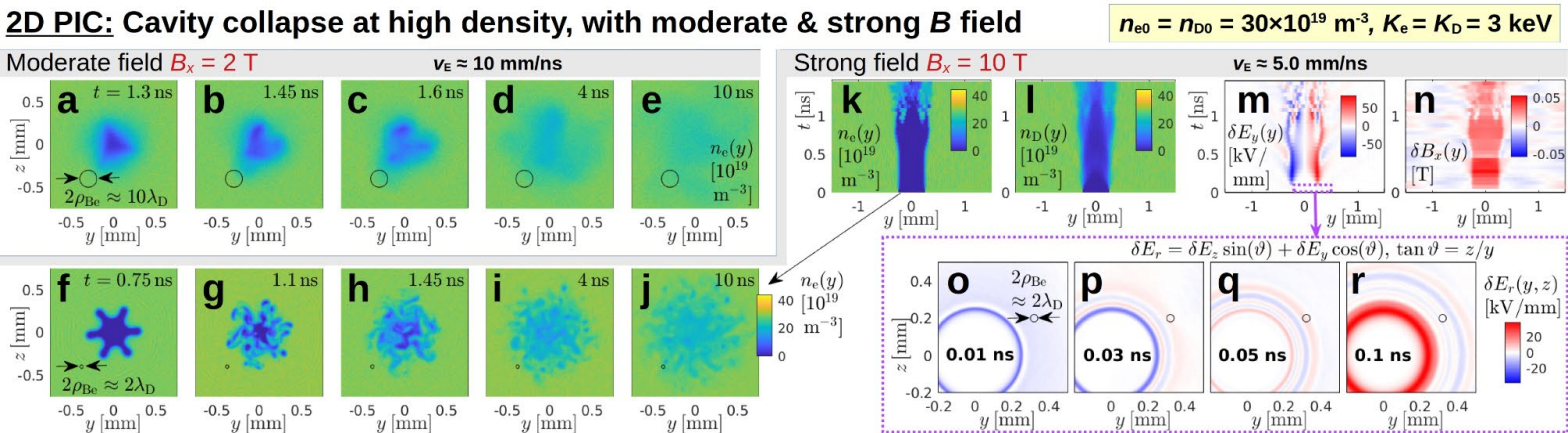
Comparison between the cavity evolution in a moderate and in a strong ambient magnetic field $B_x$. Panels (**a**)–(**e**) show snapshots of the electron density field $n_{\textrm{e}}(y,z)$ for $B_x = 2\,\textrm{T}$ of Fig. 5b. Panels (**f**)–(**j**) show $n_{\textrm{e}}(y,z)$ snapshots for the high-field case, $B_x = 10\,\textrm{T}$, whose evolution can be seen in panels (**k**)–(**r**). A variant of this case and a density scan can be found in Supplementary Fig. 12.

The ambient Maxwellian plasma has a temperature of $K_{\textrm{e}} = K_{\textrm{D}} = 3\,\textrm{keV}$, so that the electrons travel at a thermal speed of about $v_{\textrm{th,e}} \approx 33\,\mathrm{mm/ns}$ and gyrate into the outer cavity within a few picoseconds. The resulting double layer (sheath) has a characteristic width comparable to the thermal gyroradius, $\rho _{\textrm{Be}} \approx 0.1\,\textrm{mm}$. The charge separation may be difficult to see in the densities $n_{\textrm{e}}(y)$ and $n_{\textrm{D}}(y)$ plotted in Fig. 5b,c, but the associated electric field is clearly visible in Fig. 5d, where the electric perturbation $\delta E_y(y)$ is shown. (In general, the sheath’s fine structure can be more complicated and multi-layered as we will see later in Fig. 6.) The associated ${\varvec{E}}\times {\varvec{B}}$ drift of the electrons is counter-clockwise in the (*y*, *z*) plane, which should produce a magnetic perturbation $\delta B_x < 0$ that counters the ambient magnetic field $B_x = 2\,\textrm{T}$ inside the cavity. However, Fig. 5e shows that the magnetic field inside the cavity is enhanced by a perturbation $\delta B_x > 0$ with positive sign (appearing in red color), which must be due to the electrons’ magnetization current ${\varvec{j}}_{\textrm{mag}}$. All this belongs to stage 1, during which the ions still have hardly moved. The dominance of the magnetization current for magnetic induction persists throughout the simulation.

The ions impinge onto the cavity with their thermal velocity $v_{\textrm{th,D}} \approx 5.6\,\mathrm{mm/ns}$, which becomes noticeable within a few $100\,\textrm{ps}$ for our sub-millimeter-sized cavity and is labeled as stage 2 in Fig. 5. Panel (c) of Fig. 5 shows that the ion cavity tends to collapse partially during the interval $0.1\,\textrm{ns} \lesssim t \lesssim 0.5\,\textrm{ns}$, and the electrons in panel (b) follow suit to some extent, while being constrained by magnetization. One can see, by comparing panels (b) and (c) in Fig. 5 around $t \approx 0.3\,\textrm{ns}$, that the ion influx exceeds that of the electrons. The resulting reversal of the electric field $\delta E_y(y)$ is clearly visible in Fig. 5d. The associated ${\varvec{E}}\times {\varvec{B}}$ drift of the electrons also flips sign—now oriented clockwise in the (*y*, *z*) plane—and produces a magnetic perturbation $\delta B_x > 0$ that adds to the enhancement of the ambient magnetic field $B_x = 2\,\textrm{T}$ in Fig. 5e. Together with the field induced by the magnetization current, $\delta B_x/B_x$ reaches about $10\%$ ($0.2\,\textrm{T}$) in the present case.

The rapid formation (stage 1) and reversal (stage 2) of the electric field launches an electromagnetic (EM) pulse that can be seen in Fig. 5d,e during $0.3\,\textrm{ns} \lesssim t \lesssim 1.5\,\textrm{ns}$ in the form of a perturbation that travels away from the cavity with a phase velocity of about $v_{\textrm{EM}} \approx 1\,\mathrm{mm/ns}$, which corresponds to $0.5\%$ of the speed of light *c* in vacuum. Its effective wavelength is about $\lambda _{\textrm{EM}} \approx 2\,\textrm{mm}$, which corresponds to a frequency on the order of $f_{\textrm{EM}} = v_{\textrm{EM}}/\lambda _{\textrm{EM}} \approx 0.5\,\textrm{GHz}$. It is interesting that the wave’s phase velocity happens to be close to the Alfvén speed of the ambient plasma, $v_{\textrm{A}} = B_x/(\mu _0 n_{\textrm{D0}} m_{\textrm{D}})^{1/2} \approx 1.8\,\mathrm{mm/ns}$, which may facilitate resonant interactions with Alfvén waves or, rather, with their kinetic counterparts, since the wave number $k_{\textrm{EM}} \equiv 2\pi /\lambda _{\textrm{EM}}$ is large in the sense that $k_{\textrm{EM}}\rho _{\textrm{BD}} \sim 20 \gg 1$. The practical implications, if any, remain to be understood.

In the present case with relatively high density, the deuteron influx and the associated electric perturbation seem to overshoot. The ensuing Coulomb explosion restores about $80\%$ of the initial ion cavity depth by the end of stage 2. This is followed by a relatively quiescent phase that may last a few nanoseconds and is referred to here as stage 3. The details of the force balance that underlies this quasi-steady state remain to be understood. We speculate that a key role is played by the electric field ${\varvec{E}}$, which diverts the ions around the cavity if $|\delta E_y|/|v_{\textrm{th,i}}B_x| \sim v_{\textrm{E}}/v_{\textrm{th,i}} \gg 1$, as we anticipated earlier in connection with Eq. (7). Indeed, we find that $v_{\textrm{E}}(t = 1\,\textrm{ns}) \approx (20\,\mathrm{kV/mm}) / (2\,\textrm{T}) = 10\,\mathrm{mm/ns}$ is much faster than the thermal speed $v_{\textrm{th,D}} \approx 0.5\,\mathrm{mm/ns}$ with which our gyrating $3\,\textrm{keV}$ deuterons impinge on the cavity in this case.

Our approximately circular cavity—whose cross-section during the quasi-steady state is shown in Fig. 5n—is unstable to deformations, which become visible at about $t \approx 1.5\,\textrm{ns}$ in the density profiles in Fig. 5b,c. We have not yet attempted to clarify the physical mechanisms that cause such instabilities, so our discussion of this matter in the following paragraphs will remain mostly limited to the phenomenological level. During stage 4, the cavity in Fig. 5 disintegrates and the fragments seem to disappear via thermal erosion during the course of a few nanoseconds. At $t = 10\,\textrm{ns}$, which is the end of our simulation, panels (j), (k) and (o) of Fig. 5 show that very little remains of our cavity in the present high-density case.

**Fig. 7:**
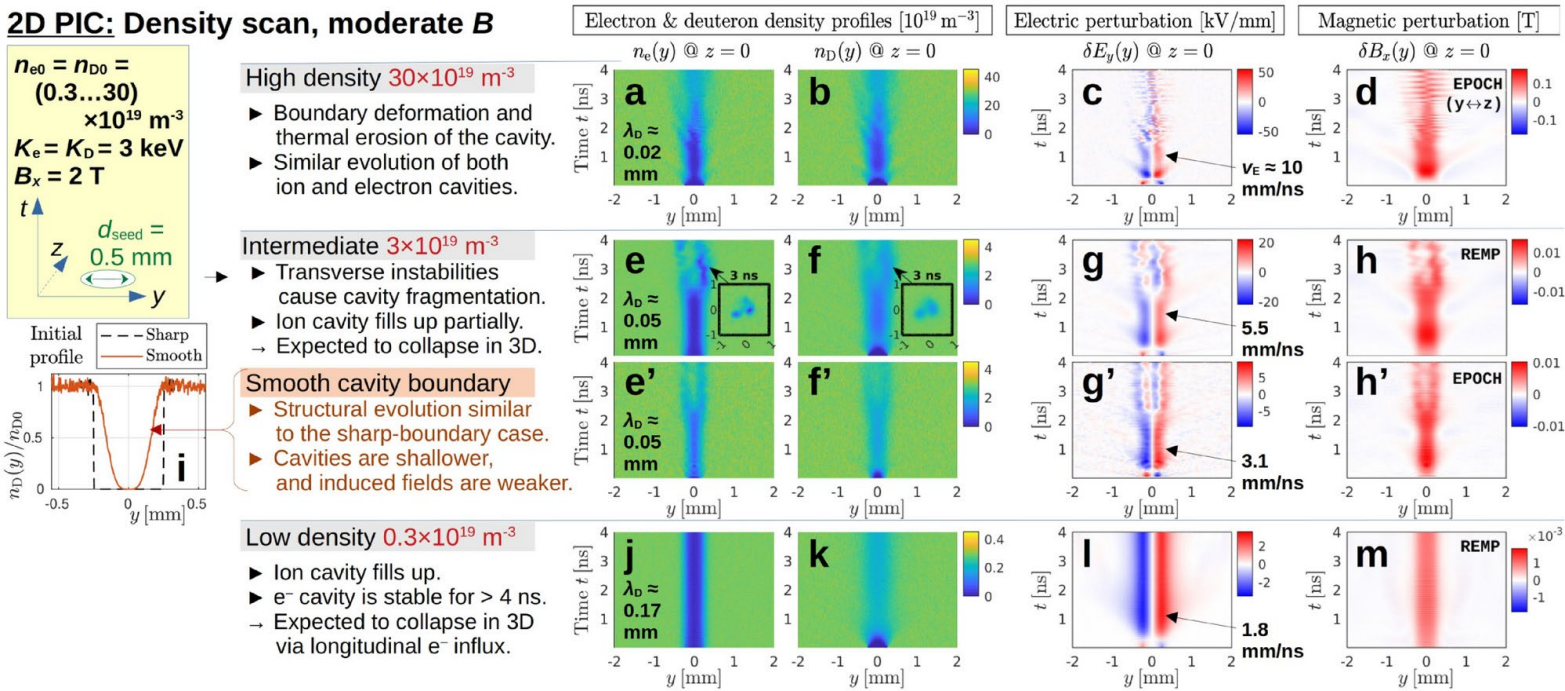
Results of 2D PIC simulations using EPOCH and REMP with densities in the range $(0.3 \cdots 30)\times 10^{19}\,\textrm{m}^{-3}$. As in Fig. 5, the color contour plots show the evolution of $n_{\textrm{e}}(y)$, $n_{\textrm{D}}(y)$, $\delta E_y(y)$ and $\delta B_x(y)$, arranged column-wise. For each case, the values of the Debye length $\lambda _{\textrm{D}}$ from Eq. (5) and the ${\varvec{E}}\times {\varvec{B}}$ velocity $v_{\textrm{E}}$ at $t \approx 1\,\textrm{ns}$ are shown in columns (**a**) and (**c**), respectively. The high-density case ($30\times 10^{19}\,\textrm{m}^{-3}$) in panels (**a**)–(**d**) is essentially identical to that in Fig. 5b–n, demonstrating excellent agreement between the two codes in 2D. Here, a 3 times smaller box ($4\,\textrm{mm}\times 4\,\textrm{mm}$) was used since only the first $4\,\textrm{ns}$ were simulated, during which thermal deuterons travel about $2\,\textrm{mm}$. For the case with intermediate density ($3\times 10^{19}\,\textrm{m}^{-3}$), the results for the default (sharply bounded) seed cavity in (**e**)–(**h**) are complemented in (**e’**)–(**h’**) with results of simulations initialized with a smoothly bounded seed cavity (motivated by Fig. 2d). The initial density profiles with sharp and smooth cavity boundary are shown in panel (**i**) on the left-hand side. The main observations are described next to column (**a**) and in the text. More snapshots of the fragmentation process in the intermediate-density case (**e**)–(**h**) can be found in Supplementary Fig. 11, which also contains additional results for another initial condition. Notice that the low-density case ($0.3\times 10^{19}\textrm{m}^{-3}$) in panels (**j**)–(**m**) has $\lambda _{\textrm{D}} \sim d_{\textrm{seed}}/2$, which permits significant charge separation on the cavity scale.

In the following paragraphs, we discuss how the dynamics of our 2D cavity depend on the strength of the ambient magnetic field and the plasma density, based on the results shown in Figs. 6 and 7. The plasma temperature is kept at $3\,\textrm{keV}$ in all cases.

#### Cavity boundary instabilities and effect of a stronger magnetic field giving $\rho _{\textrm{Be}} \ll d_{\textrm{seed}}$ (Fig. 6)

When the cavity collapses during stage 4 in Fig. 5, at the end of the quasi-steady phase, one can observe a deformation of the cavity boundary, followed by a fragmentation of the cavity itself. While the original symmetry breaking is presumably due to PIC noise and numerical inaccuracies, the instability develops in a relatively coherent fashion. The five snapshots in Fig. 6a–e show the form of this deformation in the electron density field $n_{\textrm{e}}(y,z)$. First, the circular cavity acquires a triangular shape (a). Then it breaks up into a few fragments (b,c), which eventually disappear in a diffuse blur (d,e). The diffusion is expected to be a consequence of electron gyration, whose orbit is indicated as a black circle with diameter $2\rho _{\textrm{Be}} \approx 0.2\,\textrm{mm}$ in Fig. 6a–e. Evidently, the gyroorbit’s size is similar to that of the seed cavity and its fragments.

Figure 6f–j shows how this instability is modified when the strength of the ambient magnetic field is increased by a factor 5 to $B_x = 10\,\textrm{T}$, so that $2\rho _{\textrm{Be}} \approx 0.04\,\textrm{mm} \approx 2\lambda _{\textrm{D}} \ll d_{\textrm{seed}}$. Instead of a triangular distortion, we now observe a boundary deformation in the form of a period-6 ripple that grows to a size comparable to the cavity radius as seen in panel (f). This structure then decays into many smaller fragments of scale $\rho _{\textrm{Be}}$ in panel (g), which seems to lead to a turbulent state in (h)–(j).

The first $1.5\,\textrm{ns}$ of the cavity’s evolution are shown in Fig. 6k–n. At first glance, the density cavities in (k) and (l) appear robust during the first few $100\,\textrm{ps}$, but the electromagnetic perturbations in (m) and (n) reveal low-frequency (GHz) pulsations right from the start. It remains to be clarified whether the cavity boundary is linearly unstable to perturbations of arbitrary amplitude, or whether it is destabilized nonlinearly via an earlier overshoot of the electromagnetic fluctuations (or both).

The early sheath dynamics in the present case are more complicated than what we discussed in stage 1 of Fig. 5, where we had $\rho _{\textrm{Be}} \approx 5\lambda _{\textrm{D}}$. In the present case, with $\rho _{\textrm{Be}} \approx \lambda _{\textrm{D}}$, a multi-layered sheath develops that can be seen in Fig. 6o–q, which shows snapshots of the radial electric field $\delta E_r = \delta E_z\sin \vartheta + \delta E_y\cos \vartheta$ with $\vartheta = \arctan (z/y).$ However, within $100\,\textrm{ps},$ the ion influx establishes again a positive $\delta E_r > 0$ at the cavity boundary, as can be seen in Fig. 6r, whose polarization remains unchanged until the cavity collapses. Notice also that, as long as the original cavity remains intact, we have $v_{\textrm{E}}/v_{\textrm{th,D}} \approx 5.0/0.5 = 10 \gg 1$, which is consistent with our earlier assertion that ion impingement is hindered by electric kicks.

#### Plasma density scan from small ($\lambda _{\textrm{D}} \ll d_{\textrm{seed}}/2$) to large Debye length ($\lambda _{\textrm{D}} \sim d_{\textrm{seed}}/2$) (Fig. 7)

The results in Figs. 5 and 6 were obtained with a high density of $30\times 10^{19}\,\textrm{m}^{-3}$, that may be found in the core of a tokamak plasma. Results of a density scan towards lower densities relevant for the tokamak edge are shown in Fig. 7. The ambient field strength of $B_x = 2\,\textrm{T}$ is the same as before. For easier comparison, Fig. 7a–d shows again the results of the high-density case, but now simulated using EPOCH.^11^ The results are essentially identical to those of REMP^24^ in Fig. 5b–e. Note that the values of $\lambda _{\textrm{De}}$ and $v_{\textrm{E}}$ referred to in the following discussion can be found, respectively, in columns (a) and (c) of Fig. 7.

As we have discussed in the previous section, both the electron and ion cavities in the high density case ($30\times 10^{19}\,\textrm{m}^{-3}$) are temporarily restored after an early partial implosion, but they eventually collapse within a few nanoseconds through what appear to be transverse instabilities. At high density, $n_{\textrm{e}}$ and $n_{\textrm{D}}$ in Fig. 7a,b evolve in a similar fashion when viewed on the spatial scale of the seed cavity, because $\lambda _{\textrm{D}} \ll d_{\textrm{seed}}/2$ prohibits significant charge separation on that scale. This situation changes when one proceeds to lower densities—namely, $3\times 10^{19}\,\textrm{m}^{-3}$ in Fig. 7e–h, and $0.3\times 10^{19}\,\textrm{m}^{-3}$ in Fig. 7j–m, which are of interest for the plasma edge — where the Debye length $\lambda _{\textrm{D}}$ approaches the size of our seed cavity radius $d_{\textrm{seed}}/2$. The consequence is that the electron cavity remains deep during the quasi-steady phase in panels (e) and (j), while an increasing number of gyrating ions passes through the cavity in panels (f) and (k).

The cavity in the high-density case undergoes triangular distortion and fragmentation within $1.5\,\textrm{ns}$, as we saw in Fig. 6a–c. In contrast, the quasi-steady state in the intermediate case in Fig. 7e–h lasts about $2\,\textrm{ns}$, and—as the insets in panels (e) and (f) show—the cavity here undergoes elliptic distortion and decays first into a pair of fragments, which then decay further. (See Supplementary Fig. 11 for details and additional results obtained with another initial condition.)

In order to test whether the sharp boundary of the seed cavity is a cause of the ensuing instability, we repeated the simulation for the intermediate density with a seed cavity that started with a smooth boundary density profile of the form $\sin ^4(\pi r/d_{\textrm{seed}})$ for $r^2 = y^2 + z^2 \le (d_{\textrm{seed}}/2)^2$. This is motivated by the observations in Fig. 2d. The smoothed profile we used is plotted as a solid curve in Fig. 7i and approximately resembles the shape of the cavity seen at about $0.8\,\textrm{ns}$ in Fig. 5j. The results are shown in Fig. 7e’–h’, where one can see that the overall dynamics are similar to what we saw in panels (e)–(h), both in structure and duration. At least for the present parameters, we see neither a stabilizing nor a destabilizing effect of the initial cavity profile. The main difference is that the electromagnetic perturbations $\delta E_y$ and $\delta B_x$ are only about half as strong in the case with smooth cavity boundary. (We note that the noisy ripples in Fig. 7g’ are most likely aliasing artifacts, since that simulation used a 15 times lower snapshot sampling rate than in panel (g).)

Notice that the electromagnetic perturbations in Fig. 7g’,h’ still exhibit some overshoot around $0.3\,\textrm{ns} \lesssim t \lesssim 1\,\textrm{ns},$ which supports our earlier speculations that overshoots may be a cause of the ensuing instability. In contrast, there is hardly any overshoot in panels (l) and (m), where the electromagnetic perturbations of the low-density case ($0.3\times 10^{19}\,\textrm{m}^{-3}$) are plotted. The ion cavity in panel (k) fails to recover from its initial collapse and remains shallow thereafter. This observation is consistent with the fact that $\lambda _{\textrm{D}} \sim d_{\textrm{seed}}/2$ in the low-density case, which implies that significant charge separation across seed-cavity-scale distances is now possible. Moreover, the magnitude of $\delta E_y$ in Fig. 7l implies that the velocity ratio $v_{\textrm{E}}/v_{\textrm{th,D}} \sim 1.8$ is of order unity in the low-density case, which means that the magnitude of the electric field may not suffice to divert impinging thermal ions. This consistency with observations can be taken as evidence to support our earlier conjecture that the electric deflection of ions may play a role in preventing them from flooding the cavity in the high-density case, where we had $v_{\textrm{E}}/v_{\textrm{th,D}} \sim 10$.

To summarize these results: the overshoot and subsequent Coulomb explosion seen in the high-density case is weaker in the intermediate case, and absent in the low-density case. Meanwhile, the electrons in Fig. 7j are held back by the magnetic field and their cavity remains deep and robust for the remainder of this 2D simulation. A spectral analysis shows that the transformed cavity is only subject to rapid oscillations at the upper hybrid frequency^26^ $\omega _{\textrm{UH}} = (\omega _{\textrm{pe}}^2 + \omega _{\textrm{Be}}^2)^{1/2} \approx 2\pi \times 57.4\,\textrm{GHz}$ (see Supplementary Fig. 14(i,j)), but it otherwise appears stable in 2D for at least $4\,\textrm{ns}$ and perhaps much longer.

Of course, in three dimensions—with a laser wake channel of finite length and a curved magnetic field—the positively charged cavity seen in Fig. 7j can be expected to fill up rapidly via longitudinal electron influx along $B_x$. In our $3\,\textrm{keV}$ example, the thermal electron speed is $v_{\textrm{th,e}} \sim 33\,\textrm{mm}/\textrm{ns}$, so that a $100\,\textrm{mm}$ long cavity can be expected to be neutralized within $10\,\textrm{ns}$ via longitudinal thermal streaming, which will presumably be enhanced by the attractive positive charge inside the ion-filled cavity. The next question is then whether the cavity will disappear without a trace or whether it will spawn solitary micro-cavities as seen in Fig. 4. Addressing this question will require 3D simulations, whose realization may require some more ingenuity.

## Discussion and conclusions

In the present study, we have taken first steps in the exploration of laser-plasma interactions in a new parameter regime, where a relativistically intense laser pulse travels through a typical MCF plasma and causes a highly localized but strong perturbation of the electron density in its path. We have made simplifications with the purpose of developing our physical intuition and looking for interesting and potentially exploitable phenomena for subsequent study. The simplifications that were made here for the sake of quickly gaining first insights imply that uncertainties remain and no strong conclusion can be drawn yet. For instance, the instabilities we saw in 2D simulations with intermediate and high density may be overestimated, because they may at least in part be due to an overshoot that occurred at the beginning of those simulations. Although the observed behavior appears to be physically reasonable, it remains to be validated with realistically shaped cavity density profiles and velocity distributions that resemble more closely the conditions around an actual laser-induced cavity. A preliminary discussion of this matter can be found in Supplementary Sec. 3.2. Nevertheless, the fundamental processes and general trends that we observed and discussed are expected to be robust.

Our simulations, performed in a simplified 1D+2D setting as described in Fig. 3 suggest that a laser-induced vacuum cavity faces unfavourable conditions in a hot tokamak plasma, which may limit the original cavity’s life time to a mere few nanoseconds. At a high particle density of $30\times 10^{19}\,\textrm{m}^{-3}$, which can be achieved in the central core of a tokamak, our seed cavity with initially circular cross-section and a diameter of $d_{\textrm{seed}} = 0.5\,\textrm{mm}$ collapsed due to boundary instabilities and thermal erosion on the nanosecond time scale. A stronger magnetic field did not prevent the collapse in this case. It merely reduced the size of the fragments, which tend to be on the electron gyroradius scale. At a low density of $0.3\times 10^{19}\,\textrm{m}^{-3}$ that may be found in a tokamak plasma’s remote outskirts, the large Debye length $\lambda _{\textrm{D}} \sim d_{\textrm{seed}}/2$ allowed the cavity to be flooded by gyrating thermal ions. Meanwhile, the electron cavity remained intact and seemed stable in 2D. However, such a positively charged cavity is expected to collapse in the third dimension via longitudinal influx of electrons along the magnetic field. At intermediate densities around $3\times 10^{19}\,\textrm{m}^{-3}$ that are typical for the tokamak periphery and edge, the cavity is predicted to suffer from transverse fragmentation, thermal erosion and particle influx in all directions.

The sub-mm diameter $d_{\textrm{seed}}$ of the seed cavity chosen in our study was motivated by constraints accounting for the presently available laser power and the beam line geometry in a tokamak device (see Supplementary Sec. 1.4). In order to eliminate the influx of gyrating ions, the laser wake channel would have to be significantly wider than the typical diameter $2\rho _{\textrm{Bi}}$ of the thermal ion gyroorbits. Even with high fields on the order of $B \sim 10\,\textrm{T},$ the laser pulse energy would have to be increased by more than an order of magnitude to realize conditions with $d_{\textrm{seed}} > 2\rho _{\textrm{Bi}}$ for plasma temperatures on the order of $3 \cdots 10\,\textrm{keV}$.

If the original cavity disintegrates inevitably as our simulations suggest, there is a chance that its fragments will form solitary structures that—like ideal solitons—may be long-lived. Indeed, solitary micro-cavities are seen to form from the fragments of the seed cavity in our simplified 1D (Fig. 4) and 2D simulations (Figs. 2 and 7e). Electromagnetic solitons that form in the electron population at near-critical density ($n_{\textrm{e}}/n_{\textrm{crit}} \gtrsim 0.1$) by trapping frequency-shifted laser light at scales of the electron plasma wavelength without the involvement of ions were already shown to exist in multiple dimensions and in warm or hot^27^ plasma (see Ref.^28^ for a review). In contrast, the structures we observed here form after the ion response, so they are more reminiscent of so-called post-solitons^19,25^. The solitary micro-cavities in our simulations are influenced by electron magnetization and charge separation effects on the respective scales $\rho _{\textrm{Be}}$ and $\lambda _{\textrm{De}}$, so one may speak of “Larmor-Debye solitons”.

We find the observation of such micro-cavities under MCF plasma conditions ($n_{\textrm{e}} \ll n_{\textrm{crit}},$ $K \sim 3 \cdots 30\,\textrm{keV},$ $B \sim 2 \cdots 10\,\textrm{T}$) encouraging because we envision that, if they can be created in sufficiently large numbers, the resulting micro-cavity clusters may open avenues for plasma diagnostics and control applications, which originally motivated this research (see Supplementary Sec. 1). Before that, one should verify the formation of solitary micro-cavities behind intense laser pulses in a more realistic 3D setting.

The information gained from our simplified 1D+2D simulations will be useful for the design of 3D simulations and suitable diagnostics needed to analyze their complex dynamics. One may also experiment with laser pulses that have circular instead of linear polarization or orbital angular momentum (so-called Laguerre pulses). In addition, one should examine the isotope-dependence of the dynamics and the influence of high-*Z* impurities, which may be significant in the tokamak edge. For instance, preliminary simulations for a magnetized cold plasma show that cavities in a deuterium plasma evolve differently from those in a hydrogen plasma under otherwise identical conditions (see Supplementary Fig. 9).

Aside from the long-term dynamics of solitary structures, one may also study in more detail the electromagnetic radiation that is emitted from the seed cavity during its early relaxation process. For instance, in one of the cases that we simulated, we observed an electromagnetic pulse that propagated with a phase velocity similar to the ambient Alfvén speed (Fig. 5). If that radiation is detectable, the strong spatial and temporal localization of its source may be useful for plasma diagnostics; for instance, to measure the magnetic field line pitch with high precision in regions where other methods fail. A second, delayed and weaker laser pulse may be also used to diagnose the plasma response, as in the established pump-probe method.

Another factor that may be of interest for practical applications is the strength of the magnetic perturbation. In our idealized 2D simulations, the magnitude of the magnetic field perturbation $\delta B_x$ stays below $0.1\,\textrm{T}$ for edge plasma parameters, where the electron density $n_{\textrm{e}}$ is limited to about $10^{20}\,\textrm{m}^{-3}$ in tokamaks^10^. Note that the above $\delta B_x$ is the component *along* the ambient magnetic field $B_x$, whereas the transverse components $\delta B_{y,z}$ were at noise level in the present 2D simulations and below $0.01\,\textrm{T}$ in 3D simulations of a cold plasma (cf. Fig. 12 in Ref.^12^). We also note that the amplitude of the perturbation $\delta B_x$ was found to decrease with increasing ambient field strength $B_x$ (cf. Figs. 5e,i and 6n). We expect these results to be fairly robust, since they are largely determined by the maximally achievable current density $|{\varvec{j}}|_{\textrm{max}} \sim e n_{\textrm{e}} c$, which is constrained by the ambient electron density. The generation of strong magnetic field perturbations that exceed background fluctuations would require a significant increase in electron density, which can be realized in MCF devices only transiently; for instance, through the injection of cryogenic pellets that consist of hydrogen isotopes and may contain certain high-*Z* impurities^29^.

To conclude, let us borrow an example from biology as an inspiring analogy: Just like bubble-nets allow certain whale species to hunt and feed with minimal effort, laser-induced clouds of solitary micro-cavities producing a porous plasma domain may have some attractive applications, so the properties and dynamics of this exotic plasma state should be explored further. Ultimately, we believe that, experiments are required for a conclusive test of ideas concerning the feasibility and utility of laser-assisted regulation of fusion plasmas in reactors, which may include new techniques for initiation, diagnostics and control as outlined in Supplementary Sec. 1. Meanwhile, further simulations can help to gather the evidence that is needed to invest resources and effort into such experimental tests.

## Methods

### Physical model, codes and benchmark

The equations solved are Maxwell’s equations for the electric and magnetic field vectors ${\varvec{E}}$ and ${\varvec{B}}$,

8 $$\begin{aligned} \frac{1}{c^2}\frac{\partial {\varvec{E}}}{\partial t} = {\varvec{\nabla }}\times {\varvec{B}} - \mu _0\sum _\alpha {\varvec{j}}_\alpha , \qquad \frac{\partial {\varvec{B}}}{\partial t} = -{\varvec{\nabla }}\times {\varvec{E}}, \qquad -{\varvec{\nabla }}\cdot {\varvec{E}} = \frac{\sum _\alpha q_\alpha n_\alpha }{\epsilon _0}, \qquad {\varvec{\nabla }}\cdot {\varvec{B}} = 0, \end{aligned}$$

which are written here in SI units with speed of light $c = (\epsilon _0\mu _0)^{-1/2}$, vacuum permittivity $\epsilon _0$, and vacuum permeability $\mu _0$. The subscript $\alpha$ labels the species, electrons and ions, with electric charge $q_\alpha$ and rest mass $m_\alpha.$ The particle number density $n_\alpha$ and current density vector ${\varvec{j}}_\alpha$ are obtained using suitable constitutive relations for finite-sized simulation particles, here labeled by the subscript *s*, that are defined by a particle-in-cell (PIC) form-factor (shape function), and whose positions ${\varvec{x}}_s(t)$ and velocities ${\varvec{u}}_s(t)$ obey the relativistic Newton-Lorentz equations of motion,

9 $$\begin{aligned} \frac{\textrm{d}{\varvec{u}}_s}{\textrm{d}t} = \frac{q_s}{m_s} \left( {\varvec{E}}({\varvec{x}}_s,t) + \frac{{\varvec{u}}_s}{\gamma _s}\times {\varvec{B}}({\varvec{x}}_s,t)\right) , \qquad \frac{\textrm{d}{\varvec{x}}_s}{\textrm{d}t} = \frac{{\varvec{u}}_s}{\gamma _s}, \qquad \gamma _s = \left( 1 + \left| \frac{{\varvec{u}}_s}{c}\right| ^2\right) ^{1/2}. \end{aligned}$$

For implementation details, see the documentations for the codes REMP^24^ and EPOCH^11^. Further discussions of methods and related results can be found in our previous paper^12^.

Note that the right-handed coordinates $(x',y',z')$ of the original REMP simulations were relabeled as (*y*, *z*, *x*), with *x* serving as the direction of the laser channel axis and ambient magnetic field. For the present 1D and 2D simulations, EPOCH results were found to agree well with those of REMP, except that the orientation of the structures in the (*y*, *z*) plane may differ randomly. For this reason, we flipped the transverse coordinates ($y\leftrightarrow z$) in Fig. 7a–d from EPOCH, in order to demonstrate agreement with Fig. 5b–e from REMP.

### Discretization

All simulations were run with time steps $\Delta t \le 0.999\times \Delta t_{\textrm{CFL}}$ that were shorter than the Courant-Friedrichs-Lewy (CFL) limit. Many convergence tests were performed with respect to spatial resolution and the number of particles per cell (ppc) in order to ascertain that the results are reliable. A few examples of such sensitivity tests are shown in Supplementary Fig. 13. Below we summarize the default values for the numerical parameters used in the runs whose results were shown in the paper. We emphasize that our default meshes had 40 grid points per thermal electron gyroradius $\rho _{\textrm{Be}}$ and at least 9 grid points per electron Debye length $\lambda _{\textrm{De}}$, even in the high-density case ($30\times 10^{19}\,\textrm{m}^{-3}$).

The 1D runs in Fig. 4 used a box of size $L_x = 12\,\textrm{mm}$ that was divided into $N_x = \text {4800}$ cells, with $N_{\textrm{ppc}} = 400$ simulation particles per cell and per species (electrons and deuterons).

The $10\,\textrm{ns}$ run in 2D with $B_x = 2\,\textrm{T}$ in Fig. 5 was performed with a $L_y \times L_z = 12\,\textrm{mm}\times 12\,\textrm{mm}$ large simulation box that was discretized by a uniform mesh consisting of $N_y\times N_z = \text {4800}\times \text {4800}$ grid points and initialized with $N_{\textrm{ppc}} = 40$ particles per cell and per species. The $4\,\textrm{ns}$ runs in Fig. 7 used the same resolution but a box that was three times smaller in both dimensions ($4\,\textrm{mm}\times 4\,\textrm{mm}$), since thermal ions traveled only a third of the distance. In order to maintain a resolution of 40 samples per electron gyroradius, the high-field case with $B_x = 10\,\textrm{T}$ in Fig. 6 was run with a 5 times larger number of grid points per unit length. Meanwhile, the size of the simulation box was reduced to $3\,\textrm{mm}\times 3\,\textrm{mm}$, since the ion gyroradii were smaller.

The surface plot in Fig. 4g and the profile snapshots in panels (j)–(m) of Fig. 5 were smoothed by averaging over 15 grid points, and only every third data point is plotted (a raw image can be seen in Fig. 7i). The diagnostic time resolution along the vertical axes of Fig. 7e–h,j–m is $\Delta t_{\textrm{dg}} = 3.3\,\textrm{ps}$, which is sufficient to resolve and uniquely distinguish frequencies up to $150\,\textrm{GHz}$. All other time traces were sampled at a 15 times lower rate, giving $\Delta t_{\textrm{dg}} = 50\,\textrm{ps}$, so they may contain some aliasing artifacts (see the spectral analysis in Supplementary Fig. 14).

## Supplementary Information

Supplementary information

## Data Availability

The datasets used and analyzed in the current study are available from the corresponding author on reasonable request. REMP is maintained by its developer, Timur Zh. Esirkepov, and currently subject to regulations of National Institutes for Quantum Science and Technology (QST). EPOCH is currently freely available under GPLv3.
