# Supplementary material for: Evolution of a laser wake cavity in a MCF plasma
Source: Sci Rep. 2024 Nov 13;14:27853. doi: 10.1038/s41598-024-77739-2 (PMC11560945; doi:10.1038/s41598-024-77739-2)
Supplement: Supplementary file 1 — Supplementary information [file 41598_2024_77739_MOESM1_ESM.pdf]

Supplementary material for "Evolution of a laser wake cavity in a MCF plasma"

Andreas Bierwage[1,2*], Timur Zh. Esirkepov[3+], James K. Koga[3+], Alexander S. Pirozhkov[3+], Nobuyuki Aiba[2,1], Kai Huang[3], Masaki Kando[3], Hiromitsu Kiriyama[3], Akinobu Matsuyama[4,1], Kouji Shinohara[5,2], Masatoshi Yagi[1], and Gunsu S. Yun[6,7]

[1]National Institutes for Quantum Science and Technology (QST), Rokkasho Institute for Fusion Energy, Rokkasho, Aomori 039-3212, Japan
[2]QST, Naka Institute for Fusion Science and Technology, Naka, Ibaraki 311-0193, Japan
[3]QST, Kansai Institute for Photon Science (KPSI), Kizugawa, Kyoto 619-0215, Japan
[4]Graduate School of Energy Science, Kyoto University, Uji 611-0011, Japan
[5]Dept. of Complexity Science and Engineering, The University of Tokyo, Kashiwa, Chiba 277-8561, Japan
[6]Dept. of Physics, Pohang University of Science and Technology (POSTECH), Pohang, Gyeongbuk 37673, Republic of Korea
[7]Division of Advanced Nuclear Engineering, POSTECH, Pohang, Gyeongbuk 37673, Republic of Korea
[+]These authors contributed equally: Timur Zh. Esirkepov, James K. Koga and Alexander S. Pirozhkov.
[*]e-mail: bierwage.andreas@qst.go.jp

ABSTRACT

A laser pulse focused to relativistic intensity inside a magnetically confined fusion (MCF) plasma plows away all electrons in its path. The ensuing Coulomb explosion of the ions leaves behind a cavity of microscopic size, with gradients in the electric potential and plasma density orders of magnitude stronger than anything the plasma could generate spontaneously. When posing questions concerning the practical utility of such an exotic perturbation, the life time and structural evolution of the cavity are of interest. Our simulations in a simplified 1D+2D setting and otherwise realistic parameters suggest that a sub-mm wide seed cavity (meant to resemble the laser wake channel) collapses or disintegrates within 10 ns. The dynamics are sensitive to the relative scales of the cavity, Debye shielding and gyration. We find evidence for the possibility that the collapsing seed cavity spawns solitary micro-cavities. It remains to be seen whether such structures form and survive long enough in a 3D setting to alter the local plasma conditions (e.g., as micro-cavity clusters) in ways that may be utilized for practical purposes such as plasma initiation, diagnostics or control.

Contents

1 LASART: Physical & technical feasibility of high-power laser applications in tokamaks 2
1.1 Application ideas: Laser-induced plasma breakdown & controlled transport barrier leakage (Supplementary Fig. 1) 3
1.2 Edge-localized modes (ELM): Subjective review of physics and control problems 3
1.3 Scaled experiments: Long-lived laser wake channel boundaries in unmagnetized cold gas (Supplementary Figs. 2 & 3) 4
1.4 Technical constraints on the choice of lasers: Relativistic focus & window size (Supplementary Fig. 1) 7
1.5 Laser interaction with sparse hot plasma: Transparency to near-/mid-infrared light & wake structure (Supplementary Figs. 4 & 5) 7
1.6 Constraints on laser pulse trains and recycling: Plasma drifts in an H-mode tokamak edge 9
1.7 Considerations regarding magnetic reconnection: Topology, resonance & low perturbation strength 10
1.8 Status summary: Evolution of a laser-induced plasma cavity (Supplementary Fig. 6) 11
2 Additional results for long-lived micro-cavities in 1D PIC simulations 12
2.1 Sensitivity check in 1D: Scans of seed cavity length and temperatures (Supplementary Fig. 7) 12
3 Additional results for cavity dynamics in 2D PIC simulations 13
3.1 Sensitivity check in 2D: Cold plasma and isotope effect (Supplementary Figs. 8–10) 13
3.2 Sensitivity check in 2D: Effect of elevated density ridge around the seed cavity (Supplementary Fig. 11) 16
3.3 Sensitivity check in 2D: Density scan in a strong ambient magnetic field (Supplementary Fig. 12) 17
3.4 Sensitivity check in 2D: Spatial resolution and number of particles per cell (Supplementary Fig. 13) 17
3.5 Spectral analysis of electric and magnetic fluctuations in hot deuterium plasma (Supplementary Fig. 14) 18
References 20

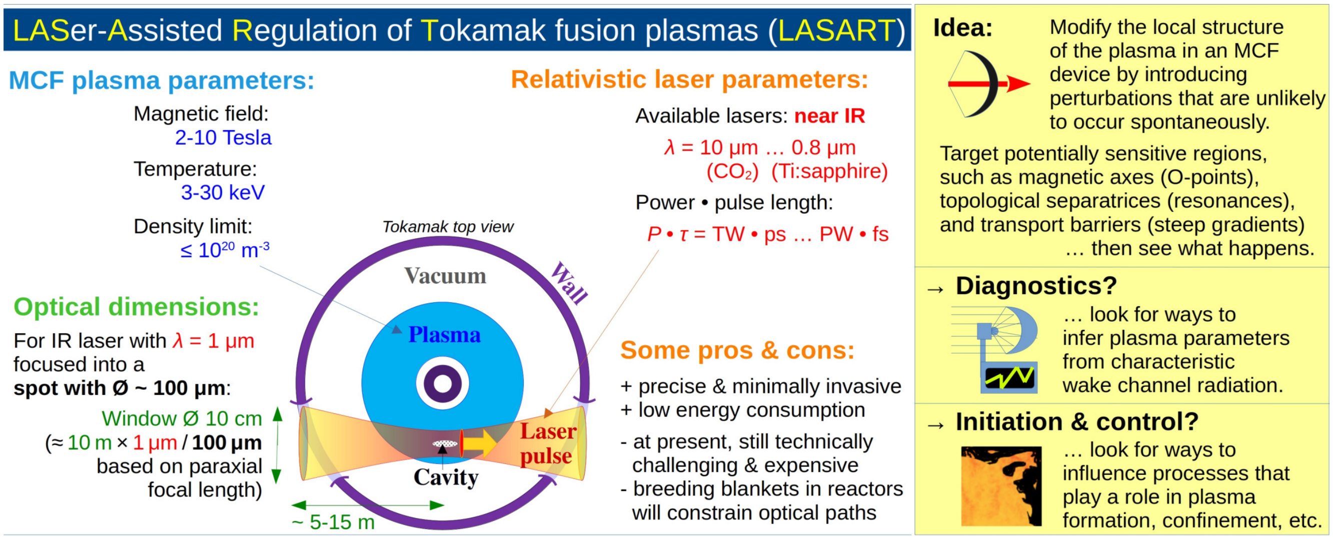

Supplementary Fig 1. Outline of the objective of project "LASART". The first stage of our feasibility study (2017–present), which was reported in Refs.[1,2] and in this paper, focused on the numerical study of the early plasma response after being perturbed by a relativistically intense laser pulse. This work was originally motivated by the need for better control of plasma edge transport.

1 LASART: Physical & technical feasibility of high-power laser applications in tokamaks

Our exploratory research on "Laser-assisted regulation of tokamak fusion plasmas (LASART)" envisions the injection of relativistic laser pulses into a tokamak fusion plasma as shown schematically in the center of Supplementary Fig. 1, where we have also listed a few pros (+) and cons (–). On the plus side, such lasers can be applied with high precision in both time and space. They are minimally invasive in the sense that they allow to apply a strong perturbation only where intended. This feature is linked to their low energy consumption compared to conventional antenna heating and fast particle beam injection, which is another notable advantage. On the minus side, high-power lasers are still plagued by technical challenges and are relatively expensive, although this is partly due to the fact that currently operating systems of this kind are designed to be versatile research tools whose capabilities exceed our needs here by far. We believe that a dedicated laser system trimmed for our purposes could be simpler, more robust, more efficient, and less expensive than the existing cutting-edge research models like J-KAREN-P. It should be noted however that, even if such a dedicated laser can be built and if useful perturbations can be produced at certain injection angles in principle, we anticipate that difficult compromises will have to be struck in order to open the required optical paths through the massive tritium breeding blankets that will line the inner wall of a deuterium-tritium fusion reactor.

In this study, we constrain our analysis to MCF plasma and laser parameter values that are realistic, so that an experimental test would be feasible in the 'foreseeable' future. Although there are reasons to be skeptical and one may suspect that "*not much would happen*", we decided to take a closer look. In Refs.[1,2] and in this paper, we took first steps using mostly numerical methods. The result is not yet conclusive and we encourage further studies — preferably experimental tests — because something *did* happen in the simulations, and we think it is interesting to explore whether there are useful implications.

For diagnostics applications, one does not really want too much to happen to the plasma. This avenue is, however, not actively explored here. This is a goal-oriented research project, and our first priority is to gather information about the feasibility of urgently needed control applications. Two concrete ideas — one of which is less, the other more speculative — are outlined in the following Supplementary Sec. 1.1: laser-induced breakdown (LIB), and controlled transport barrier leakage.

The following notes may perhaps best be viewed as a collection of quick, back-of-the-envelope estimates and guesses that we have collected over the course of about six years. Due to the complexity of the problem, many such estimates and guesses had to be made in order to survey the largely unexplored terrain from start to goal based on limited prior knowledge. The amount of material, even if preliminary, warrants its collection here for further critical consideration. The speculatory parts are to be replaced step-by-step with results of more accurate calculations, such as those reported in the main paper. The following notes are also meant to describe the broader context within which this work was performed. The question we ask at the beginning of the main paper about the possible utility of high power laser pulses for MCF plasma initiation, diagnostics and control is still open, but we believe to have made a few steps towards its answer by simulating the early stages of the plasma response (main paper) and initiating discussions concerning possible roadblocks and opportunities in the following paragraphs.

1.1 Application ideas: Laser-induced plasma breakdown & controlled transport barrier leakage (Supplementary Fig. 1)

For control applications, we do not know how much perturbation is good enough. It is only obvious that the intense laser pulse itself does not carry enough energy (some Joules) and deposits even less energy in the plasma (which is effectively transparent) to have any *direct* impact on the overall energy budget of an MCF plasma.

In the case of laser-induced breakdown (LIB) — whose application to superconducting tokamaks is a possible avenue for future research — the laser is meant to provide additional seed charges, while the acceleration energy is provided externally. Here, the motivating problem is that it can be challenging to reliably trigger a Townsend avalanche in superconducting tokamaks due to practical constraints that limit the allowed loop voltage.[3,4] This problem can already be tackled by providing a sufficient number of sufficiently powerful gyrotrons that assist the breakdown process through electron cyclotron heating. However, it is preferable to optimize as many gyrotrons as possible for operation at keV rather than eV temperatures, so an alternative method would be welcome. Laser pulses may provide such an alternative. For instance, the nonneutral cavities seen in the simulations reported in the present paper may assist in providing the additional seed charges needed to initiate a plasma with low loop voltage. Moreover, the pulses can, in principle, be applied precisely in those regions of the prefill gas volume where an avalanche is most easily triggered; namely, near magnetic axes (a.k.a. "magnetic nulls" of the poloidal field).

In the case of edge transport control — which is the problem that originally motivated this project — the energy to be released will come from the plasma itself. The laser is not meant to (and cannot) 'make the container spill over'. Instead the idea is to induce additional leakage by poking holes into that 'container' (here an MCF transport barrier). This basic idea is not new by any means; it is likely to date back to prehistoric ages. One purpose of the present study is to find out what kind of 'holes' we can produce with intense laser pulses. The fact that we performed only simulations in a simplified 1D+2D setup implies that the evidence we have found so far is only weak evidence. However, we did not find nothing; we found solitary micro-cavities. Whether these can serve the role of metaphorical 'leak holes in a container' remains to be seen. The next step is to determine whether the (nonneutral) micro-cavities seen in our simplified 1D+2D simulations can form in a 3D setting and, if so, how long they will survive. Their survival time will presumably have an impact on the possible applications.

We suspect that micro-cavities may have to be produced in sufficiently large numbers to change the *local plasma state* in a way that might affect the way how the plasma edge regulates its pressure gradients. Here, the wording 'plasma state' underlines the fact that we consider more than mere energy content (which is not affected by the laser pulses considered) and inspect also structural changes, which can be induced by intense laser pulses, as our simulations have demonstrated. If we can produce an exotic plasma state — such as a porous domain populated by nonneutral solitary micro-cavities that may or may not be localized toroidally — one can proceed to examining the question whether and, if so, how this exotic plasma state may affect the way in which energy is transported across adjacent magnetic flux surfaces or flux tubes. The desired outcome would be a continuous well-dosed leakage of excess pressure under conditions of overall high confinement (preferably high confinement of energy, less of particles). More concretely and perhaps more importantly, our aim is the efficient and reliable prevention of spontaneous large plasmoid ejection events known as edge-localized modes (ELM)[5–7] of Type-I.

1.2 Edge-localized modes (ELM): Subjective review of physics and control problems

Steep gradients in plasma density and temperature can be maintained by so-called transport barriers in a MCF plasma.[8,9] While the barrier physics are still a subject of intensive research, there is much evidence suggesting that magnetic geometry, turbulence and sheared rotation are key ingredients. A hand-waving and possibly controversial explanation may go like this: *Turbulence contains a zonal flow component, which is a form of sheared rotation. Above a certain threshold of injected power (heating or beams) or self-generated power input (fusion reactions), and in the presence of a geometric constraint — such as magnetic shear reversal or a magnetic separatrix — the turbulent energy transfer can stagnate and be diverted into strong zonal flows that reinforce the stagnation of transport. Metastable distortions in the magnetic geometry in response to shifts in the local and global force balance may contribute, and the large ion-electron mass ratio and impurities may also play a role.*

Irrespective whether or not this is an accurate depiction of the events occurring in a real tokamak plasma, it seems reasonable to assume that, as a general rule, the accumulation of energy and material, and the associated steepening of gradients cannot continue indefinitely. If the residual fluxes across the barrier do not suffice, the barrier must ultimately rupture in one way or another. In the case of the celebrated edge transport barrier that provides the so-called high-confinement mode (H-mode),[10] it appears that real tokamak plasmas often realize this relaxation in the form of ELMs, which are a subject of intensive research and whose avoidance or mitigation is a high-priority issue for ITER experiments[11] and DEMO power plants.

Researchers have found that the venting of energy across the H-mode edge barrier can occur in various ways and in multiple stages that could be related to the normal and nonnormal modes of perturbation that were mentioned in the introduction of the main paper. For instance, normal modes in the form of so-called edge harmonic oscillations contribute to toroidal symmetry breaking and provide a mechanism for continuous pressure venting with tolerable heat loads on plasma-facing components and exhaust structures.[12] Unfortunately, this attractive regime tends to be fragile due to a variety of factors, one of which is sheared

plasma rotation, including zonal flows. And, also unfortunately, when the plasma parameters are optimized for confinement performance and efficiency, the self-regulation of the H-mode edge tends to occur in the form of abrupt plasmoid ejection events known as Type-I ELMs, which are somewhat reminiscent of coronal mass ejections on the Sun. Such events cannot be tolerated in reactor-grade devices such as ITER and DEMO due to potential damage to plasma-facing components of the device.

Various techniques for Type-I ELM avoidance exist, the most reliable ones being ELM suppression/mitigation using resonant magnetic perturbations (RMP)[13,14] and perhaps ELM pacing using cryogenic pellets.[15] These techniques are expected to be crucial for the success of ITER, but it is not clear whether they can be routinely used in a power plant. Both pellets and RMP coil fields constitute significant perturbations that may have adverse effects elsewhere. Pellets may cause over-fueling that overloads not only the confined plasma but also the cryo-pumps outside. RMP coils must be carefully tuned,[16] and even then they may trigger other deleterious normal-mode-like instabilities or plasma deformations. In addition, RMPs may enhance losses of energetic particles such as beam ions and fusion-born alpha particles that may damage plasma-facing components even without ELMs. Moreover, the fact that external RMP coils would have to be extremely massive, and in-vessel RMP coils should be avoided in a deuterium-tritium fusion reactor with its high neutron fluxes, poses further obstacles for their application. Therefore, alternative paths for ELM control and mitigation continue to be explored,[17–19] and one may ask: *Can intense laser pulses be used for precisely timed and minimally invasive venting of the edge transport barrier?*

This idea was stimulated by a discovery made around 2010. Using advanced electron cyclotron emission (ECE) imaging techniques that allowed to observe the evolution of edge dynamics before, during and after an ELM crash in unprecedented detail, it was found that a solitary perturbation appears before the onset of the crash. This solitary structure can be clearly distinguished from the normal-mode-like oscillations that exist at earlier times: (i) Unlike the helically fluted quasi-2D structure of a normal eigenmode and "blob" filaments,[20,21] there appears to be a 3D localized protrusion, somewhat similar to a solar prominence. The structure also has (ii) a higher amplitude and (iii) a different velocity than the eigenmode-like fluctuations that are seen at earlier times. The solitary structures were observed on several devices; in particular, KSTAR[22–24], ASDEX-Upgrade[25] and EAST[26]. The occurrence of large collapses seems to correlate with the occurrence of solitary structures.[24] The details of the process remain unclear, but there exists evidence that sheared flows may play a crucial role. For instance, such flows can produce a spectrum of nearly degenerate modes that — according to nonmodal stability theory[27] — may then develop nonnormal solitary structures and boost them into the nonlinear regime, where they can become long-lived.[28–32] The solitary filament bursts during ELM events in experiments were also found to be associated with radio-frequency wave emissions,[33,34] including whistler waves,[35] which may be interpreted as evidence for fast 3D magnetic reconnection. Our question then becomes: *Can an intense laser pulse trigger the formation of such a self-sustained solitary structure, and can it do so below the threshold where a disastrous Type-I ELM would occur, for instance, by seeding a 3D magnetic reconnection event?*

Evidently, the questions we posed above consist of much speculation and uncertainty, in part, because nonnormal modes and magnetic reconnection — both their evolution and effects — are topics of ongoing research, as are transport barriers and ELMs. We do not know what is needed to make H-mode operation both safe and efficient in a fusion power plant. The present study should thus be viewed as a curiosity-driven exploration that focuses on a small part of a larger problem.

The ELM problem is a serious one and we think that it makes sense to grasp at any straws that one can possibly find. As mentioned in the introduction of this Supplementary Sec. 1, the advantage of laser pulses compared to other ELM mitigation and ELM pacing techniques like pellet injection and magnetic perturbation coils would be their high precision, minimal invasiveness, and low energy consumption. Reported in this paper are the latest results obtained during the project's first exploratory phase (2017–present), which was primarily concerned with the physical feasibility of the concept.[1,2] Other ideas to address the ELM problem also continue to be explored,[17–19] and the final solution could be a combination of various measures. Moreover, the insights gained from the LASART project may have applications other than those we had originally considered, and we do, of course, hope that the present paper will stimulate new ideas and contribute to the development of robust solutions.

In the following subsections, we discuss various aspects related to these matters. This feasibility study is not complete, so the following notes only reflect the current status of our survey of evidence, ideas and projections. There are many gaps that are tentatively patched by guesses with the goal of enabling us to anticipate major roadblocks and opportunities that may lie ahead, and choose our research path accordingly.

1.3 Scaled experiments: Long-lived laser wake channel boundaries in unmagnetized cold gas (Supplementary Figs. 2 & 3)

In 2018, a series of experiments exploring burst intensification by singularity emitting radiation (BISER) in multi-stream flows[36] and laser wake field acceleration (LWFA)[37] was performed at the J-KAREN-P laser facility,[38–40] which offered an opportunity to take images of laser-induced plasma channels in cold hydrogen and helium gas jets. During the preparations for those experiments, some shots were made at relatively low densities around $10^{23}...10^{24}\,\mathrm{m}^{-3}$, where self-focusing effects and resulting instabilities are effectively nil, so that the observations may be scaled to tokamak densities that are another three to six orders of magnitude lower: around $10^{18}\,\mathrm{m}^{-3}$ for the prefill gas used for plasma initiation and $10^{19}...10^{20}\,\mathrm{m}^{-3}$ for steady-state

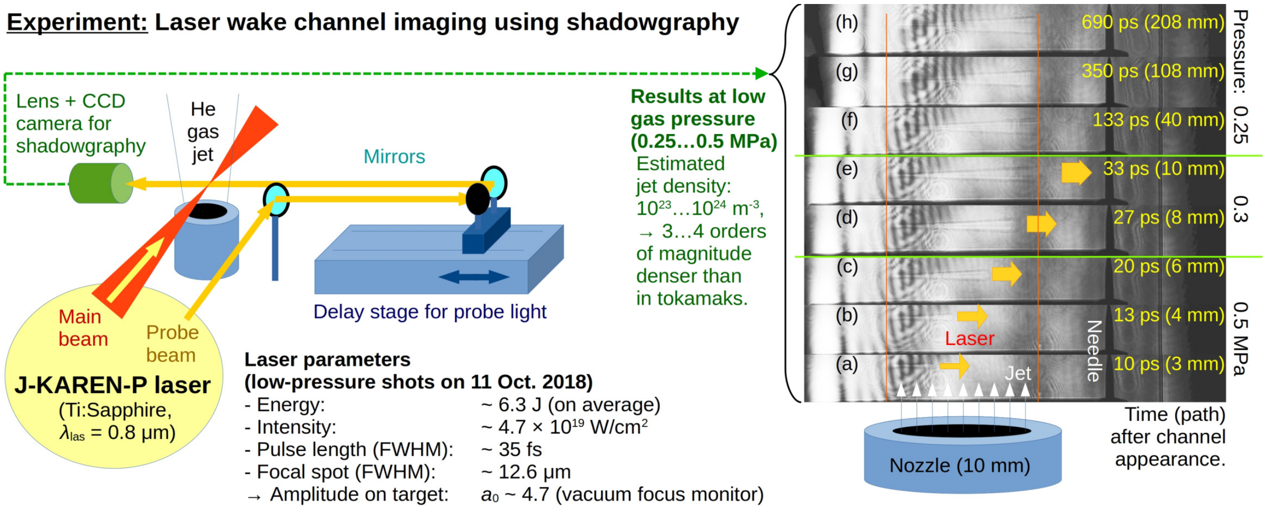

Supplementary Fig 2. Laser wake channel imaging using shadowgraphy on J-KAREN-P. The experimental setup and parameters are shown on the left. On the right, we present a sequence of eight snapshots labeled (a)–(h), showing the evolution of the channel's shadow within the first nanosecond after the laser pulse entered the gas jet. (Fig. 1(a) of the main paper contains a reduced set of these data.) These data were collected during preparations for experiments that had other purposes, so that the gas pressure (see the right edge of the figure) and nozzle position varied somewhat. The images were aligned using the calibration needle appearing in black in the right part of each snapshot. The orange arrow moving rightward indicates the position of the 35 fs long laser pulse. The focal spot (about 12.6 μm wide) was close to the laser's point of entrance to the supersonic gas jet, which is located here near the left edge of the nozzle below panel (a), and whose inner diameter was 10 mm.

operation. Although the interpretation of the experimental observations is not straightforward as discussed below, we consider them to be a necessary prerequisite to justify the numerical study of laser wake channel dynamics in sparse plasmas on long time scales.

The relevant portion of the experimental setup and the operational parameters are shown on the left-hand side of Supplementary Fig. 2. Here, the main beam passed through a helium gas jet and the interaction region was imaged by a transversely incident probe beam using the shadowgraphy technique. The probe beam had been split off from the main beam beforehand, and the timing of the image was controlled by a set of mirrors, two of which are mounted on a long delay stage. In these experiments, the laser's normalized amplitude defined in Eq. (1) of the main paper was estimated to reach the highly relativistic regime with $a_0 \sim 4.7$ at the focal spot.

The right-hand side of Supplementary Fig. 2 shows a sequence of snapshots of the channel's shadow. During the first 20 ps, one can clearly see that a channel, appearing in light gray, formed behind the laser pulse. The darker stripes above and below are interpreted as the channel's boundary. The maximal delay realized here was about 0.7 ns, during which this presumed channel boundary appears robust. Unfortunately, the shadowgraphy technique does not allow us to determine whether the channel's interior has been refilled or remained void, because the shadow's contrast is thought to be related to the 2nd derivative of the refractive index, so that it represents variations in the electron density gradient landscape. An empty channel may be difficult to distinguish from a uniformly filled channel if the latter is still surrounded by a ridge of higher density (a remnant of the Coulomb explosion).

Be that as it may, since no significant changes are observed after the laser pulse has exited the gas jet, one may assume that at least the channel boundary is stable on the nanosecond time scale for densities around $10^{23}...10^{24}\,\mathrm{m}^{-3}$. If one then naïvely assumes that the long-term channel dynamics still scale with the Langmuir frequency (which is by no means certain), a simple extrapolation suggests that the structure could be stable on the 100 ns scale at tokamak-relevant densities. The reasoning that underlies this naïve scaling is as follows.

As an illustration, Supplementary Fig. 3 shows how the size of the structures in the laser's wake – and, hence, the time scale of their evolution —- varies with the particle density in 2D PIC simulations. Panels (a) and (b) show the situation that is thought to have been present in the laser experiments, namely an effectively cold gas with a density around $10^{23}\,\mathrm{m}^{-3}$. Panel (c) shows how the scales are stretched in both space and time when the simulation is performed with a tokamak-relevant density of $3\times10^{19}\,\mathrm{m}^{-3}$.

The time scale $\tau_{\rm wake}$ and characteristic wavelength $\lambda_{\rm wake}$ of the magnetized plasma response in the wake of a laser pulse is determined by the upper hybrid frequency[41] as

$$\tau_{\rm wake} \approx 2\pi/\omega_{\rm UH} \propto n_{\rm e}^{-1/2}, \quad \lambda_{\rm wake} = c\tau_{\rm wake} \approx 2\pi c/\omega_{\rm UH}, \tag{1}$$

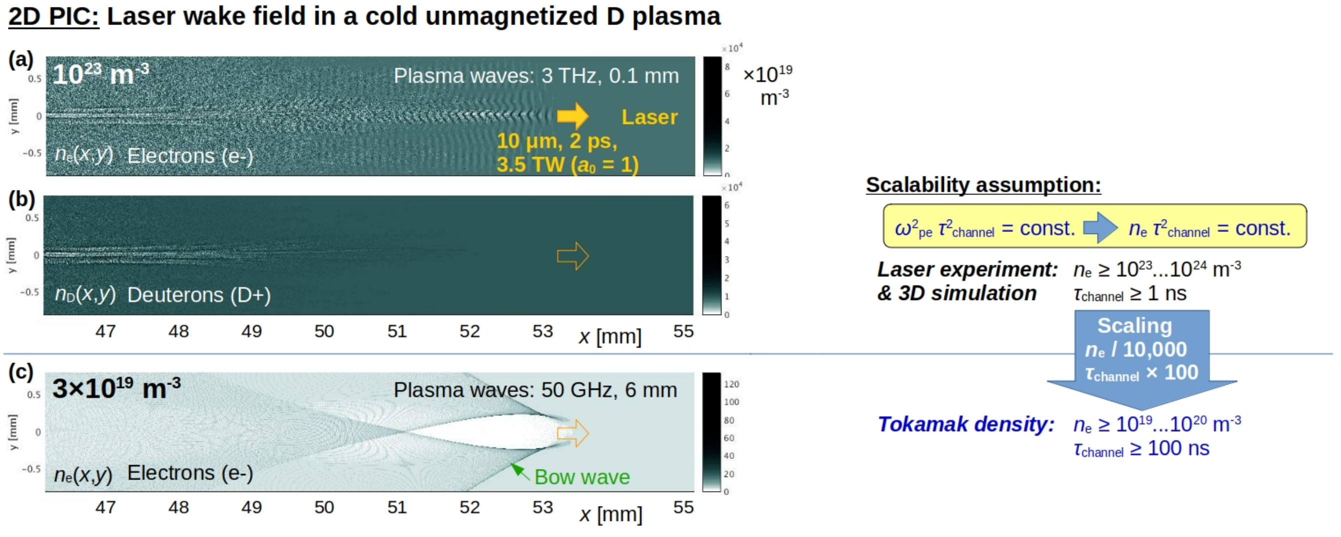

Supplementary Fig 3. Results of 2D PIC simulations of the wake field behind a relativistically intense laser pulse for densities $10^{23}\,\mathrm{m}^{-3}$ and $3\times10^{19}\,\mathrm{m}^{-3}$, illustrating the scaling arguments discussed in Supplementary Sec. 1.3. Making use of limited similarity (see Ref.[1] for details and some related 3D simulations), the simulations were performed with a scaled laser wavelength of $\lambda_{\rm las} = 10\,\mu$m and pulse length of $\tau_{\rm pulse} = 2\,$ps. The normalized amplitude was $a_0 = 1$ at the focal spot located at $x_{\rm foc} \approx 46\,$mm (left edge of the figure). The orange/yellow arrow indicates the location of the laser pulse at the time of the snapshots. Panels (a) and (b) show the electron and ion density fields for the high density expected to have been present in the experiment in Supplementary Fig. 2. Panel (c) shows the electron density field in the wake of the same laser pulse but for the low particle density of a tokamak plasma. The values of the respective frequency $\omega_{\rm pe}$ and wavelength $\lambda_{\rm pe} = 2\pi c/\omega_{\rm pe}$ of cold plasma waves are shown in the upper right corners of panels (a) and (c). No ambient magnetic field was applied here.

with

$$\omega_{\rm UH}^2 = \omega_{\rm pe}^2 + \omega_{\rm Be}^2, \quad \omega_{\rm Be} = |q_{\rm e}|B/(\gamma_{\rm e} m_{\rm e}) \approx 2\pi\times 28\,{\rm GHz}\times B\,[{\rm T}]. \tag{2}$$

For tokamak-relevant ambient field strengths $B = |\boldsymbol{B}| \sim 2...10\,$T, the gyrofrequency $\omega_{\rm Be}$ lies in the 100 GHz range. The Lamgmuir frequency $\omega_{\rm pe}$ defined in Eq. (3) of the main paper has a similar value on the order of $\sim 100\,$GHz in a tokamak and $\sim 10\,$THz in the laser experiments shown in Supplementary Fig. 2. For the purpose of order-of-magnitude estimates as we perform here, one may ignore the effect of the magnetic field and assume $\tau_{\rm wake} \sim 2\pi/\omega_{\rm pe}$. The point is that, for $\omega_{\rm pe}^2/\omega_{\rm Be}^2 \sim 9\times n_{\rm e}[10^{20}\,{\rm m}^{-3}]/(B[{\rm T}])^2 \gtrsim 1$, the characteristic time scale of the channel dynamics is proportional to $n_{\rm e}^{-1/2}$ and essentially independent of B. Our assumption is then that the channel life time $\tau_{\rm channel}$ scales with the wake period $\tau_{\rm wake}$ as

$$\tau_{\rm channel}^2/\tau_{\rm wake}^2 = {\rm const.} \quad \Rightarrow \quad n_{\rm e}\tau_{\rm channel}^2 \approx {\rm const.} \tag{3}$$

If our naïve assumptions hold, a 10,000-fold decrease in the plasma density should then yield a 100-fold increase in the channel's life time. Applied to our cold gas example, this means that the life time of at least 1 ns that we inferred from the laser experiment in Supplementary Fig. 2 should translate to a lower bound of the channel life time that is on the order of 100 ns for tokamak-relevant densities.

While this result is promising, one may anticipate that the channel's stability may be underestimated due to the absence of an ambient magnetic field in the above experiments, and overestimated due to the fact that a cold gas was used instead of a hot one, although a certain amount of (yet-to-be-diagnosed) heating is thought to occur during the laser pre-pulse. While the ambient magnetic field certainly has a stabilizing effect in the early stages of the formation of a vacuum cavity in the laser wake channel, the field's long-term effect is not obvious and may depend on other parameters, such as density (see Fig. 6 of the main paper and Supplementary Fig. 12 in Supplementary Sec. 3.3). Longitudinal inflow of charged particles is also limited in the above experiment by the fact that the channel spans the entire width of the gas jet in Supplementary Fig. 2, whereas the laser wake channel in a tokamak with its curved magnetic field lines would be connected to a large reservoir of hot charged particles. For instance, deuterons with a "temperature" (thermal energy) of $K_{\rm D} = m_{\rm D}v_{\rm th,D}^2/2 \sim 3\,$keV have a thermal speed of $v_{\rm th,D} \sim 0.5\,{\rm mm/ns}$, so they may cross the narrow channel of width $d \sim 0.5\,$mm on the nanosecond time scale.

Clearly, it is necessary to understand the effects of magnetization and thermal motion, which were absent in the above scaling exercise and are the theme of the main paper. Meanwhile, we continue here with discussions of related technical aspects and try to foresee the possible long-term evolution of the observed laser-induced perturbation and its potential uses.

1.4 Technical constraints on the choice of lasers: Relativistic focus & window size (Supplementary Fig. 1)

The relativistic regime $a_0 \gtrsim 1$ can be routinely achieved with laser wavelengths $\lambda_{\rm las} \sim 1\,\mu$m (e.g., solid-state Ti:sapphire lasers such as J-KAREN-P[38–40]) and perhaps with some effort using $10\,\mu$m CO_2 gas lasers.[42,43] In the future, free electron lasers (FEL) may also be considered, since quantum-level coherence is not required for our purposes. The classical coherence of FEL light is sufficient. We only require rapid electron acceleration to relativistic speeds, which classically coherent light achieves with high efficiency. Advantages of FELs include their tunable frequencies and high repetition rates up to GHz levels.[44,45] Intra-cavity experiments can already be performed with fairly high power, but extracting intense light is still challenging.[46]

Longer wavelengths may appear to be preferable since their lower critical density $n_{\rm crit} \propto \lambda_{\rm las}^{-2}$ implies stronger absorption. However, the absorption should be kept low enough for the laser pulse to be able to reach the desired destination without too much attenuation. Moreover, a longer wavelength $\lambda_{\rm las}$ has a shorter Rayleigh length that, in turn, influences the required size of optical components, including the window through which the laser enters the vacuum vessel. Below are some estimates.

The focal length can be characterized by the Rayleigh length $L_{\rm R}$,

$$L_{\rm R} \equiv \frac{\pi w_{\rm foc}^2}{\lambda_{\rm las}} = \frac{\pi}{2\ln 2}\frac{d_{\rm foc}^2}{\lambda_{\rm las}}, \tag{4}$$

where $w_{\rm foc}$ is the $1/{\rm e}$ amplitude waist radius, and $d_{\rm foc} = w_{\rm foc}\sqrt{2\ln 2}$ is the full-width-half-maximum (FWHM) diameter of the intensity distribution. In the paraxial approximation, the FWHM diameter $d(X)$ at distance X from the focal spot is

$$d(X) \approx d_{\rm foc}\sqrt{1+X^2/L_{\rm R}^2} \overset{X\gg L_{\rm R}}{\approx} d_{\rm foc}X/L_{\rm R} \approx 0.44\times X\lambda_{\rm las}/d_{\rm foc}. \tag{5}$$

In practice, the window through which the (unfocused) laser pulse enters the vacuum chamber at a distance X from the focal spot should be larger than d by a factor 1.2 for super-Gaussian and 2...3 for Gaussian near-field beam distributions, if some clipping is allowed. Here, we shall assume that the window should be at least 2 times larger than the FWHM diameter d:

$$d_{\rm win} \gtrsim 2\times d \approx X_{\rm win}\lambda_{\rm las}/d_{\rm foc}. \tag{6}$$

For a focal spot with FWHM diameter $d_{\rm foc} \sim 100\,\mu$m as was assumed in our simulations, the Rayleigh length then becomes $L_{\rm R} \sim 0.02\,{\rm m}/\lambda_{\rm las}[\mu{\rm m}]$ and — as illustrated in Supplementary Fig. 1 — the required window diameter at a distance on the order of $X_{\rm win} \sim 10\,$m is $d_{\rm win} \sim 10\,$cm for $\lambda_{\rm las} = 1\,\mu$m, which seems technically feasible.

Evidently Eq. (6) and technical limitations for the allowable window size constrain the allowed laser wavelength to the micrometer scale. If we were to limit the window size to $d_{\rm win} = 10\,$cm and use a CO_2 gas laser with $\lambda_{\rm las} = 10\,\mu$m, the spot size would have to be increased to $d_{\rm foc} = 1\,$mm. The relation between the power $P_{\rm las}$, intensity $I_{\rm las}$, and focal spot size $d_{\rm foc}$ of a linearly polarized Gaussian laser pulse is

$$P_{\rm las} = I_{\rm las}\frac{\pi w_{\rm foc}^2}{2} = a_0^2 I_1 \frac{\pi d_{\rm foc}^2}{4\ln 2} = 1.55\times 10^{18}\,\frac{\rm W}{\rm cm^2}\times\frac{a_0^2 d_{\rm foc}^2}{(\lambda_{\rm las}\,[\mu{\rm m}])^2}, \tag{7}$$

where we used $I_{\rm las}/a_0^2 = I_1 = (1.37\times 10^{18}\,{\rm W/cm^2})/(\lambda_{\rm las}\,[\mu{\rm m}])^2$ from Eq. (1) of the main paper. Such a CO_2 laser would have to achieve a peak power of 155 TW for $a_0 = 1$. This is not far from the 100 TW power of the planned Brookhaven Experimental Supra-Terawatt Infrared at ATF (BESTIA),[43,47,48] which may pave the way for the future use of CO_2 lasers in an MCF setting.

For the time being, however, only solid-state Ti:sapphire lasers can routinely meet the above requirements. With $\lambda_{\rm las} = 0.8\,\mu$m and $d_{\rm foc} = 0.1\,$mm, such a laser can attain $a_0 = 1$ with a power of 242 TW, which is a regime that has already been well-established and characterized at J-KAREN-P.[38] The above technical constraints contributed to our decision to postpone scans of the seed cavity size, which has been fixed at $d_{\rm seed} = 0.5\,$mm in our present simulations. Instead, we examined the sensitivity of the results with respect to the seed cavity profile (see Fig. 7 of the main paper and Supplementary Sec. 3).

1.5 Laser interaction with sparse hot plasma: Transparency to near-/mid-infrared light & wake structure (Supplementary Figs. 4 & 5)

A cold plasma is effectively transparent to the kinds of lasers that can achieve relativistic intensities to date. However, tokamak plasmas can reach fairly high temperatures on the order of 10 keV, so one should also evaluate the interaction of the laser pulse with "warm" plasma waves. This appears to be still a subject of active research,[52] especially in the presence of strong density fluctuations and suprathermal electrons as is to be expected for intense laser-plasma interactions. In the simple limit of electrons having a uniform number density $n_{\rm e}$, a Maxwellian "temperature" (kinetic energy) $K_{\rm e} = m_{\rm e}v_{\rm th,e}^2/2$, thermal gyroradius $\rho_{\rm Be} = v_{\rm th,e}/\omega_{\rm Be}$, and gyrofrequency $|\omega_{\rm Be}| = eB/m_{\rm e} \approx 2\pi\times 28\,{\rm GHz}\times B[{\rm T}]$, the angular frequency ω and wavelength λ of warm plasma waves are related by the Bohm-Gross dispersion relation[53]

$$\omega(\lambda)^2 - \omega_{\rm pe}^2 = 12\pi^2\frac{\lambda_{\rm De}^2}{\lambda^2}\omega_{\rm pe}^2 = 3\frac{4\pi^2}{\lambda^2}\frac{K_{\rm e}}{m_{\rm e}} = 6\pi^2\frac{\rho_{\rm Be}^2}{\lambda^2}\omega_{\rm Be}^2 \approx \left(2\pi\times 215\,{\rm GHz}\frac{\rho_{\rm Be}}{\lambda}B\,[{\rm T}]\right)^2, \tag{8}$$

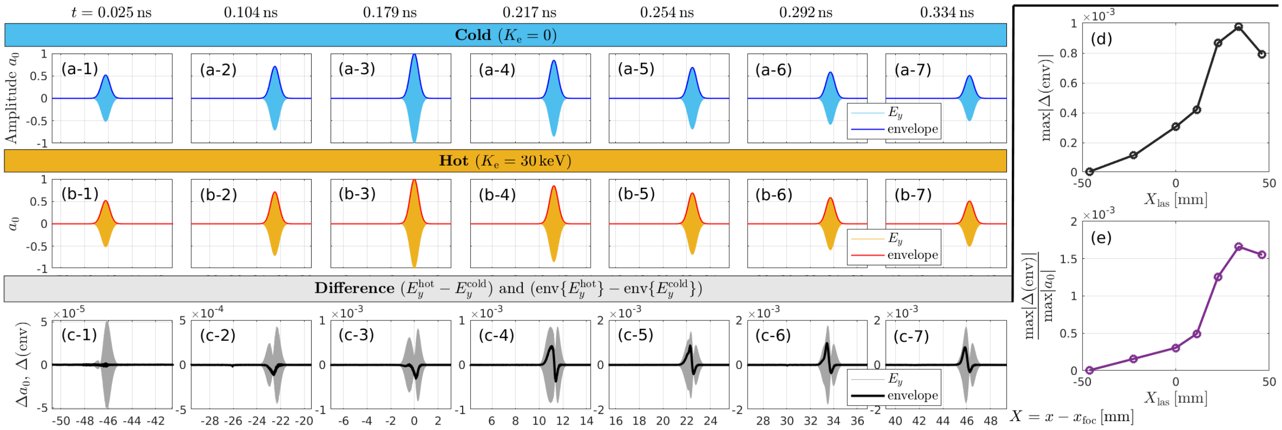

Supplementary Fig 4. Propagation of a laser pulse focused to relativistic intensity in a hot plasma. This EPOCH[49] simulation was performed with a moving window that follows a Gaussian 2 ps laser pulse with wavelength $\lambda_{las} = 4\,\mu$m. The ions are taken to be immobile and electrons are initialized with (a) zero velocity, or (b) a hot Maxwellian velocity distribution with thermal energy $K_e = m_e v_{th,e}^2/2 = 30$ keV. These are the same cases as in Fig. 1(b,c) of the main paper. We loaded $N_{ppc} = 10$ particles per cell (ppc). The simulation box is about 10 mm wide in x and follows the laser pulse for about 100 mm (0.33 ns). The focal spot, where the amplitude reaches $a_0 = 1$, is located roughly half-way at $x_{foc} \approx 50.3$ mm, and the laser pulse passes it in the third snapshot ($t \approx 0.18$ ns). Rows (a) and (b) each show seven shapshots of the amplitude of the electric field component E_y normalized to a_0 (light colors) as well as its envelope (solid line). Row (c) shows the differences between the pulse in the hot and cold plasma in terms of amplitude Δa_0 (gray) and envelope Δ(env) (black). Panel (d) shows the evolution of the peak value of the envelope difference, and panel (e) shows the same data divided by the instantaneous peak amplitude max$|a_0|$ of the laser field. One can see that the pulse distortion for hot electrons stays below 0.2% compared to the cold electron case, so the plasma remains essentially transparent in spite of the fact that $v_{th,e}/c \approx 0.33$ is not far from unity. Further study is needed to clarify the details of these results. The differences in the evolution of the laser pulses could be due to stimulated Raman scattering (SRS, see Appendix A of Ref.[50] or Chapter 7 of Ref.[51]). The sharp peak in the distortion about 30 mm behind the focal spot in (d) and (e), and its tendency to decrease afterwards could be due to a combination of the focusing geometry and the threshold condition for the SRS instability to occur.[51]

which emerges in the derivation of Landau damping (see Eq. 2.25.8 on p. 98 of Ref.[54]). Notice that the electron Debye length

$$\lambda_{De} = \frac{1}{\omega_{pe}}\left(\frac{K_e}{m_e}\right)^{1/2} = \left(\frac{\epsilon_0 K_e}{n_e e^2}\right)^{1/2} = 23.5\,\mu\text{m}\left(\frac{n_e}{10^{20}\,\text{m}^{-3}}\right)^{-1/2}\left(\frac{K_e}{1\,\text{keV}}\right)^{1/2} \tag{9}$$

for a typical tokamak plasma with multi-keV temperature is not very far from the laser wavelengths $\lambda_{las} \sim 1...10\,\mu$m nor from the electron gyroradii $\rho_{Be} \sim 20...100\,\mu$m that we considered in the introduction of the main paper.

Although the dispersion relation (8) is only valid for $\lambda_{De} \ll \lambda$ and, hence, violated here in the limit $\lambda \to \lambda_{las}$, this limit still yields a prediction that makes physical sense: given that we have $\omega_{pe} \ll \omega_{las}$ for our parameters of interest, the condition $\omega/\omega_{las} \ll 1$ for the hot plasma to be transparent to the laser pulse translates Eq. (8) into

$$\sqrt{3} v_{th,e}/c \ll \lambda/\lambda_{las} \to 1. \tag{10}$$

In other words, transparency can be assumed only if the electron thermal velocity is much smaller than the phase velocity of the laser light, namely $v_{th,e} \ll c$. From the formula

$$E = \gamma m_0 c^2 = K + m_0 c^2, \qquad \gamma = \left(1 - v^2/c^2\right)^{-1/2} \quad \Rightarrow \quad v^2/c^2 = 1 - \left(1 + K/(m_0 c^2)\right)^{-2}, \tag{11}$$

we find $v/c \approx 0.1$ for $K = 3$ keV that we used in the present study, so the transparency assumption seems to be justified here.

Meanwhile, the target temperature in the core of a reactor-grade plasma is around $K \sim 30$ keV, which corresponds to $v/c \approx 0.33$. In order to see what fate the laser pulse will encounter in this regime, we performed 2D EPOCH[49] simulations that follow the pulse for a distance of about 100 mm, centered around the focal spot. The results for $K_e = 0$ and $K_e = 30$ keV are compared in Fig. 4, which shows that the thermal distortion of the pulse stays below 0.2%. Consequently, we may regard even a reactor-grade plasma as being effectively transparent to the lasers of interest.

The wake that the laser leaves behind in the electron density is, however, strongly affected by the electron temperature as we have shown in Fig. 1(b,c) of the main paper. Another view of the same data is given in Supplementary Fig. 5(a,b), where we applied filters to enhance the visibility of the wake's structure. Supplementary Fig. 5 also illustrates the influence of a 2 T

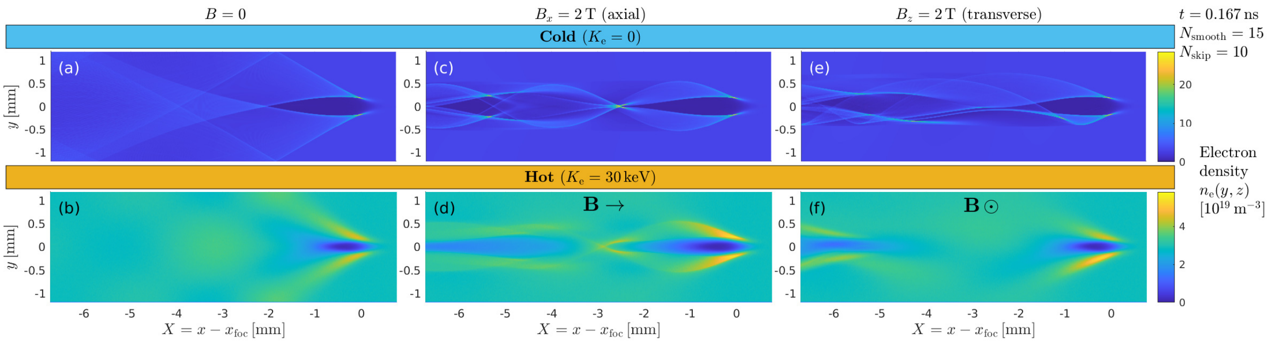

Supplementary Fig 5. Electron wake behind a relativistic laser pulse in a cold plasma (top) and with 30 keV hot Maxwellian electrons (bottom), without and with an ambient magnetic field. The unmagnetized case in (a) and (b) is the same as in Supplementary Fig. 4 above and in Fig. 1(b)–(d) of the main paper, except that the present figure shows filtered data: we averaged over $N_{\rm smooth} = 15$ grid points and then plotted only every 10th sample. This improves the visibility of the wake structure, while attenuating the cusp singularities in the cold plasma case ($\max|n_{\rm e}| \sim 120 \rightarrow 30$) and reducing the noise in the hot electron case. Periodic boundary conditions along the y-direction cause the bow wave to reenter the simulation box in (a) and (b) about 2 mm behind the laser (here located at $X_{\rm las} = 0$). Panels (c)–(f) show results obtained with a 2 T field oriented in the x- or z-direction. The cold plasma cases were presented and discussed in Fig. 2 of Ref.[1]. Note that 2D simulations give a distorted image of the effect of axial magnetization (B_x). For comparisons between 2D and 3D results, see Ref.[1].

strong ambient magnetic field $\boldsymbol{B}$ in panels (c)–(f). Here, the main effect of $\boldsymbol{B}$ is that it causes the bow wave to remain confined near the wake channel. In 3D, an axial field $B_x = 2$ T also inhibits the collapse of the wake behind the laser, so the on-axis cusp singularity near $X \approx -3$ mm in Fig. 5(c) should be viewed as an artifact of the reduction to 2D (cf. Fig. 15 of Ref.[1]).

1.6 Constraints on laser pulse trains and recycling: Plasma drifts in an H-mode tokamak edge

While axial magnetization helps to sustain the cavity that has been left behind by the relativistic laser pulse on short time scales (~ 0.1 ns), the results in Fig. 7 of the main paper suggest that a cavity whose width exceeds the Debye length is subject to fragmentation, while smaller cavities are filled with thermal ions. In order to prolong the cavity's existence, one may consider ways to reinforce it via a laser pulse train, or — taking advantage of the MCF plasma's transparency — through pulse recycling using mirrors. Besides requiring substantial technical effort, there are some physical obstacles to this idea, such as the long laser path and bulk plasma motion, which we shall discuss in this section.

In the edge of a tokamak plasma operating in so-called high-confinement mode (H-mode), the electric drift velocity associated with a radial electric field E_r is on the order of[55,56]

$$v_{\rm E} \approx E_r/B \sim 20\,{\rm kV/(m\cdot T)} = 0.02\,{\rm mm/ns}. \tag{12}$$

Similar values can be expected for the diamagnetic drift velocity $v_{*\alpha}$, which can be written

$$v_{*\alpha} \approx \frac{\nabla P_\alpha}{m_\alpha n_\alpha \omega_{{\rm B}\alpha}} \approx \frac{v_{{\rm th},\alpha}^2}{2\omega_{{\rm B}\alpha}\Delta_{P\alpha}} = \frac{v_{{\rm th},\alpha}}{2}\frac{\rho_{{\rm B}\alpha}}{\Delta_{P\alpha}}. \tag{13}$$

The subscript α labels the particle species with thermal energy $K_\alpha = m_\alpha v_{{\rm th},\alpha}^2/2$, thermal gyroradius $\rho_{{\rm B}\alpha} = v_{{\rm th},\alpha}/\omega_{{\rm B}\alpha}$, and pressure gradient scale length $\Delta_{P\alpha} = P_\alpha/|\nabla P_\alpha|$. For instance,[55] in a $B \approx 4$ T field, deuterons ($\alpha = {\rm D}$) with thermal energy $K_{\rm D} = 3$ keV and pressure gradient scale length of about 10 gyroradii, $\Delta_{P{\rm D}}/\rho_{\rm BD} \sim 10$, have a diamagnetic velocity of about $v_{*{\rm D}} \lesssim 0.03$ mm/ns. We view the laser-induced wake channel as a perturbation of the background density that (unlike drift or Alfvén waves) would be a cause rather than an object of diamagnetic drift. Thus, assuming that the channel follows only $v_{\rm E}$, it takes ~ 30 ns for a 0.5 mm wide cavity to be displaced by a distance comparable to its own diameter. Since the drift velocity is difficult to measure or predict accurately, it may be difficult to track and reinforce the moving cavity inside a very sparse plasma with a laser pulse train for more than a few dozen nanoseconds. A possibly wobbly surface and global plasma pulsations or displacements can complicate the matter further.

It seems that, even if a cavity survives the interval between successive laser pulses, the idea of reinforcing that existing cavity in the H-mode edge may be difficult to realize in large devices like ITER or DEMO. First, the above-mentioned motions are difficult to measure or predict accurately, especially in reactors that will have very limited diagnostics due to spatial constraints and high neutron fluxes. This complicates the accurate aiming of the laser. Second, it takes 33 ns for light to travel a distance of 10 m, but at least the seed cavity is unlikely to survive that long, according to the results of the main paper. (Meanwhile, shooting the laser at the solitary micro-cavities that we found in our simulations is likely to destroy them.) Therefore, we anticipate that beam recycling — if its technical challenges can be overcome — may perhaps be used for producing multiple cavities, but hardly to reinforce an existing one or its fragments.

1.7 Considerations regarding magnetic reconnection: Topology, resonance & low perturbation strength

Leaving aside questions pertaining to the technical feasibility and efficiency, let us return to questions concerning the physical feasibility of high-power laser-assisted MCF plasma control. Given the large separation of spatial scales between the meter-scale plasma and the sub-millimeter focal spot of the laser, it is natural to consider the possibility of using the laser pulse to trigger multi-scale self-organization processes that are already known to exist in magnetized plasmas. One candidate that fits the bill is magnetic reconnection. Moreover, one may expect that a laser pulse's chances to trigger a macroscopic response are largest in regions with steep pressure gradients — namely, transport barriers, which are sometimes considered to be a key ingredient for ITER and for its successors to become efficient fusion reactors. In Supplementary Sec. 1.1 above, we have discussed some speculations concerning the possibility of causing a macroscopic response in a MCF plasma by applying a microscopically small but intense perturbation with relativistically intense laser pulses. In this section, we elaborate further on the underlying physical reasoning. At the moment, this is merely an exercise of trying to find matching patterns in existing knowledge and apply those patterns to the questions at hand. As stated before, the purpose is to motivate and guide subsequent deeper research.

Nonmodal structures can become permanent if they are boosted into the nonlinear regime. Resonances may be considered to be a natural place for this to occur, because resonances are always nonlinear: mathematically, any resonant perturbation with nonzero amplitude will cause a change in phase space topology in the vicinity of the resonance concerned. That is why, in practice, resonances (including island separatrices) always acquire the form of stochastic layers due to random noise imposed on deterministic chaos. The resonances can be effectively broadened by various kinds of background fluctuations (where the definition of "background" depends on the spatio-temporal scales of interest), so one can expect that there is a scale-dependent amplitude threshold for a coherent nonlinear structure to emerge and survive in the fluctuating environment.

Magnetically confined plasmas with toroidal topology typically have a rich set of geometric resonances, which are located on toroidal surfaces where the orbit helicity h of a charged particle's trajectory has a rational value, $h = p/n$, with p and n being integers.[57] In the limit of zero mass-to-charge ratio, h becomes identical to the so-called safety factor q. In the plasma core, the profiles of q and h usually have weak to moderate gradients, so that resonances tend to be sparse and hence highly discrete, unless one considers modes with very short wavelengths (large p and n). Such resonances are also the loci of magnetic reconnection, which is effectively two-dimensional if one transforms out the helical twist. However, the situation in an H-mode edge can be different. H-modes are most often generated in diverted plasmas, which are bounded by a separatrix where $q \rightarrow \infty$ mathematically and resonances hence tend to be dense. This is thought to facilitate 3D reconnection.

It is worth noting that, due to the difference between the field helicity q and the orbit helicity h, which arises from guiding center drifts, the location and existence of resonances varies depending on a particle's mass-charge ratio, kinetic energy and velocity pitch for a given magnetic configuration. Magnetic reconnection hence affects different groups of charged particles in different ways, which, in turn, can affect the reconnection process as it relies on the plasma currents associated with charged particle motion. The implications of this are still being untangled and continue to lead to new insights.[58]

The situation is further complicated by the fact that some processes of practical interest — including magnetic reconnection itself — can occur on time scales short enough for the concept of resonance to become obscure. In the present context, resonance conditions are statements about the topological structure of the magnetic field and particle orbits, but if the field evolves while the particles are tracing it out (as is done for Poincaré analysis), the observed structure becomes diffuse. The full implications are not clear to us, with the consequence that we find it difficult to determine criteria (with respect to size, structure and life time) under which a laser-induced perturbation (or its offspring) would be able to affect transport in sensitive regions of the plasma, such as transport barriers and resonances.

Our tentative assumption is that topology must have emerged — that is, information must have propagated — via at least one toroidal transit of Alfvén waves, and that the cavity thus becomes embedded in that topological structure of the plasma. On this basis, we may postulate a critical life time of $\tau_{\rm crit} \gtrsim 1\,\mu$s. While this time scale is known to be relevant for global structure formation, it may be too restrictive for the actual trigger process that precedes the ejection of a (preferably small) plasmoid.

Another possible, and somewhat related, avenue of thought that yields a similar result for $\tau_{\rm crit}$ is to assume that the cavity should survive long enough for the plasma current to pass by its length L of, say, some $\gtrsim 10$ mm (depending on the beam's Rayleigh length and field alignment). The mean speed $v_{\rm cur}$ associated with a current $I_{\rm p}$ through a cross-section surface A is

$$v_{\rm cur} \approx \frac{I_{\rm p}}{e n_{\rm e} A} \approx 0.1\,{\rm mm/ns} \times \frac{I_{\rm p}[{\rm MA}]}{A[{\rm m}^2]} \sim 0.01\,{\rm mm/ns} \tag{14}$$

for typical tokamaks. For $L \gtrsim 10$ mm equated to $v_{\rm cur}\tau_{\rm crit}$, this yields again $\tau_{\rm crit} \gtrsim 1\,\mu$s. Here, the underlying rationale is that magnetic reconnection can be viewed as a process of current redistribution, whose time scale may thus play a role for determining how long it would take for a perturbation to have an effect on the reconnection process, which then translates into a criterion for the minimal life time of that perturbation. While the overall magnetic geometry in a tokamak is determined by the global current distribution, magnetic reconnection involves strong focusing of current in a sheet-like, ribbon-like or even point-like fashion that can also be associated with electron acceleration. Again, this means that our above requirement of regular plasma current to pass by the perturbed region could be too restrictive.

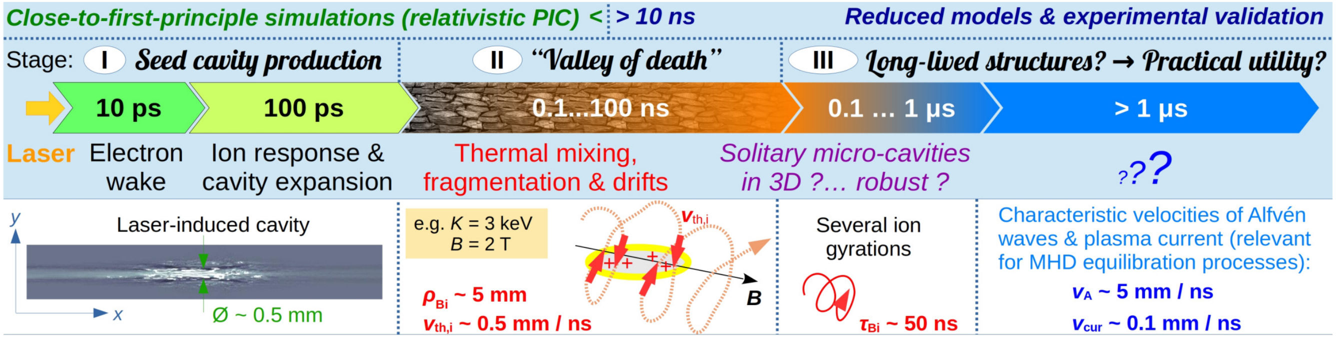

Supplementary Fig 6. Evolutionary stages of a laser-induced cavity as summarized in Supplementary Sec. 1.8. So far, we analyzed the dynamics up to time scales on the order of 10 ns, which can be covered by PIC simulations where we have found weak evidence (in a simplified 1D+2D setting) for the formation of solitary micro-cavities. If this can be confirmed in a more realistic 3D setting, and if these structures can be shown to be robust, our preliminary speculations (Supplementary Secs. 1.1 and 1.7) about the practical utility of intense laser pulses in MCF plasmas should be verified, ideally through experimental tests.

Refining the above estimates for a critical life time and determining other criteria (e.g., for the size and structure of a perturbation) for effective laser-assisted regulation of tokamak plasmas will require a better understanding on both ends: the process to be controlled (e.g., transport barrier leakage and plasmoid ejection) and the perturbation that is being applied with the purpose of control. In the present paper, we found weak evidence for the possibility that a relativistically intense laser pulse may leave behind a porous channel containing nonneutral solitary micro-cavities, and it remains to find out whether this will also happen in reality and, if so, what influence these perturbations may have. One point that seems to be relatively clear is that the magnetic perturbation $\delta B \lesssim 0.1\,\mathrm{T} \ll B$ associated with a laser-induced microscopic cavity is too small and too short-lived to have any effect on its own because we expect it to be indistinguishable from ubiquitous background fluctuations (see the Discussion section of the main paper). At present, the generation of long-lived strong perturbations of the particle density in solitary form is the only concrete *ansatz* we have found that holds at least some promise of being useful for control applications.

1.8 Status summary: Evolution of a laser-induced plasma cavity (Supplementary Fig. 6)

The purpose of this study was to obtain first insight into the response of a tokamak plasma when it is perturbed by a relativistically intense laser pulse. We distinguish three stages as illustrated in Supplementary Fig. 6: Stage I is dominated by the strong field of the laser and its wake. It begins with the laser plowing away electrons and exciting plasma wake oscillations, which are modified here by the moderately strong ambient magnetic field of a tokamak. This is followed by the ion response in the form of a Coulomb explosion. Stage I lasts about 100 ps, where thermal ion influx may still be ignored. Using the relativistic PIC codes `REMP`[59] and `EPOCH`,[49], this stage was studied in a previous paper,[1] which predicted robustness of the laser-induced cavity up to the nanosecond scale when starting from an initially cold deuterium plasma. The contour plot in the lower left corner of Supplementary Fig. 6 shows the central cavity of the 0.5 mm wide laser wake channel as seen in such a simulation.[1]

During stage II, which we studied in the present paper, the cavity was found to be subject to thermal mixing and instabilities when surrounded by a multi-keV plasma. For instance, 3 keV deuterons in a 2 T field have a gyroradius of $\rho_{\mathrm{BD}} \sim 5\,\mathrm{mm}$ that is one order of magnitude larger than the cavity diameter in our example. At low density ($\sim 0.3 \times 10^{19}\,\mathrm{m}^{-3}$) the ions can traverse our Debye-scale cavity in about 1 ns. The positive charge will attract electrons that can enter the cavity along the magnetic field. At high density ($\sim 30 \times 10^{19}\,\mathrm{m}^{-3}$), the ions are deflected by electric forces but the cavity boundary is found to become unstable in our simulation setup. In Supplementary Fig. 6, we call this interval from 0.1 to 100 ns the "*valley of death*" — a term borrowed from the jargon of start-up entrepreneurship, referring to the challenging phase from innovation to application.

If the cavity or its offspring could somehow survive through the thermal valley of death, it would enter stage III, which we have tentatively associated with the microsecond time scale and beyond (Supplementary Sec. 1.7). We define stage III to be the hypothetical regime (hence marked "???" in Supplementary Fig. 6), where the likelihood for plasma control applications (like those discussed in Supplementary Sec. 1.1) would reach a significant level. In order to overcome the valley of death, one may consider to reinforce the original seed cavity with the aid of laser pulse trains and — in a sufficiently small device and with much technical effort — through pulse recycling. However, the task of aiming the laser at a microscopic cavity is expected to be challenged by drifts and plasma rotation (Supplementary Sec. 1.6), so we tend to be skeptical about this option.

Another possibility — which is weakly supported by the latest results reported in this work — is that the laser-induced cavity may spawn solitary micro-cavities during stage II, as in Fig. 2 of the main paper. While still uncertain, we consider this to be a path that is worth to be explored further. It should be checked whether micro-cavity phenomena similar to those seen in our simplified 1D+2D setting can arise in 3D and, if so, whether these perturbations may survive into stage III. The idea is that — just like the process of nonlinear self-focusing of a relativistic laser pulse in a sufficiently dense medium[60–62] — nonnormal modes in the form of solitary micro-cavities may disregard global dispersion and boundary conditions, and dig themselves up in their own local wells.

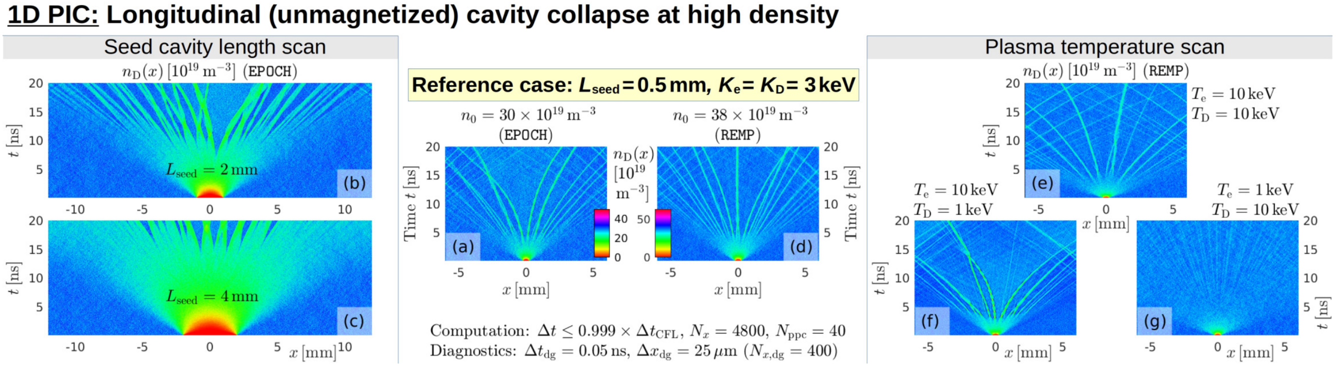

Supplementary Fig 7. Results of parameter scans for the thermal collapse of a 1D cavity in the absence of a magnetic field. As a reference case, we choose the high-density case from Fig. 4(b)–(g) of the main paper. The contour plot for the evolution of the deuteron density $n_D(x)$ shown in panel (a) was obtained using EPOCH and is equivalent to the result in Fig. 4(b), except that the parameter scans in the present figure were performed with a smaller number of particles ($N_{ppc} = 40$ instead of 400). The result of a similar simulation performed with REMP is shown in panel (d). The relevant features of the dynamics are the same in both simulations, bearing in mind that the detailed trajectories of individual solitary cavity waves are sensitive to noise. The reference case was simulated with a box of length $L_x = 12$ mm and initialized with a seed cavity of length $L_{seed} = 0.5$ mm and an ambient Maxwellian deuterium plasma with temperature $T_e = T_D = 3$ keV. Panels (b) and (c) show results of EPOCH simulations initialized with longer seed cavities, $L_{seed} = 2$ mm and 4 mm. Here, the size of the simulation box was doubled to $L_x = 24$ mm. The result in panel (c) was also reproduced with a box length of $L_x = 96$ mm (not shown). Panels (e)–(g) show results of REMP simulations initialized with different temperatures. In panel (e), the temperature was raised to 10 keV for both electrons and ions. In panels (f) and (g), we reduced either the deuteron or the electron temperature to 1 keV, respectively.

Dynamics up to the 10 ns scale — stage I and early stage II — can be explored close to first principles with relativistic PIC simulations. In contrast, the time scales beyond 10 ns — advanced stage II and stage III — require more ingenuity and one may need to resort to reduced models, which increases uncertainties and requires experimental validation for conclusive answers.

2 Additional results for long-lived micro-cavities in 1D PIC simulations

2.1 Sensitivity check in 1D: Scans of seed cavity length and temperatures (Supplementary Fig. 7)

The simplified 1D simulations of the longitudinal unmagnetized cavity dynamics in Fig. 4 of the main paper have four free parameters besides the ion's mass and charge: namely, the seed cavity length L_{seed}, the ambient plasma density n_{e0}, and the temperatures T_e and T_D. The trend for increasing density was already illustrated in Fig. 4 of the main paper. Here, we use the high-density case (30×10^{19} m^{-3}) as a reference — which is reproduced with both codes in Supplementary Figs. 7(a,d) — and vary the parameters L_{seed}, T_e and T_D.

Panels (a)–(c) show results for increasing seed cavity length $L_{seed} = 0.5$ mm, 2 mm and 4 mm at fixed temperature $T_e = T_D = 3$ keV. We observe that the solitary cavities that are located near the center of the original cavity, and which propagate only slowly, have significantly increased in size. The rapidly propagating cavities in the peripheral region tend to be smaller. This seems to be true even for the reference case in panel (a), but it becomes more obvious for the larger initial cavity lengths in (b) and (c). The velocity at which the cavity spreads out has not changed, which is as expected since the ion thermal velocity has not been altered. The duration of the diffusive phase (before the solitary cavities appear) seems to become longer with increasing L_{seed}.

Panels (d) and (e) show the effect of increasing both T_e and T_D from 3 keV to 10 keV. The only readily visible difference is the larger rate of spreading, which is consistent with the higher thermal velocity of the ions. The larger rate of spreading may also be one reason for the fact that the solitary cavities in panel (e) become sparse more rapidly than in (d), although the number of solitary cavities may, in fact, have decreased somewhat. A comparison between panels (e) and (f) shows that the solitary cavities remain sparse while the rate of spreading is reduced when T_D is reduced to 1 keV. Interestingly, however, the spreading rate does not decrease quite as much as expected. In fact, this may be due to another possible effect: namely, the spontaneous emergence of solitary cavities from ambient plasma ahead of the thermal ion front. Finally, a comparison between panels (e) and (g) shows that reducing T_e to 1 keV produces a dense cloud of barely discernible fragments that seem to spread out at what appear to be fairly constant velocities.

We have recorded these preliminary results here for the sake of completeness, but cannot currently offer physical explanations for all observations that were made. Further study is required.

3 Additional results for cavity dynamics in 2D PIC simulations

3.1 Sensitivity check in 2D: Cold plasma and isotope effect (Supplementary Figs. 8–10)

The 2D PIC simulations that we presented in Figs. 5–7 of the main paper were initialized with a circular cavity in a 3 keV hot deuterium plasma permeated by an ambient magnetic field with strength $B_x = 2\,\text{T}$. Here, we discuss results of similar 2D PIC simulations of a cavity in a cold plasma, again with a 2 T ambient field. One purpose of this exercise was to see whether the simplified 2D setting is capable of reproducing the stability of the cavity on the nanosecond time scale that we observed in earlier 3D simulations.[1] However, with the initial conditions used in Figs. 5–7 of the main paper, this system would remain trivially stable when the thermal velocity is zero or very small, so we have used instead a setup where the initial ion density is uniform and a cavity exists only in the electron distribution. As shown in Supplementary Fig. 8, the electrons from the cavity are deposited in a surrounding ridge of elevated density to maintain overall charge neutrality. This configuration is constructed as follows.

The electron and ion charge density profiles are modeled as

$$q_e n_\text{e}(r) = q_\text{e} n_\text{e0} \times \begin{cases} \epsilon & : \quad r < r_0, \\ \epsilon + 1 - y(r)/y_0 & : \quad r \geq r_0, \end{cases} \qquad \text{with} \quad y(r) = G''(r), \qquad y_0 = y(r_0), \qquad r^2 = y^2 + z^2, \tag{15a}$$

$$q_\text{i} n_\text{i}(r) = q_\text{i} n_\text{i0} \times (\epsilon + 1) = \text{const.}, \tag{15b}$$

where the function $y(r)$ defining the electron density profile is the second derivative $G'' \equiv \text{d}^2 G/\text{d}r^2$ of a bell-shaped function

$$G(r) = \exp\left(-\frac{(r/D)^\kappa}{2}\right), \qquad G'(r) = -\frac{\kappa}{2D^\kappa} r^{\kappa-1} G(r), \qquad G''(r) = \left(\frac{\kappa^2}{4D^{2\kappa}} r^{2(\kappa-1)} - \frac{\kappa(\kappa-1)}{2D^\kappa} r^{\kappa-2}\right) G(r), \tag{16}$$

as shown in Supplementary Fig. 8. The parameters chosen for our simulations are

$$n_\text{e0} = 3\times 10^{19}\,\text{m}^{-3}, \quad \epsilon = 10^{-4}, \quad D = 0.3\,\text{mm}, \quad r_0 = D\left(\frac{3(\kappa-1) - (5\kappa^2 - 6\kappa + 1)^{1/2}}{\kappa}\right)^{1/\kappa}, \quad \kappa = 5.828428, \tag{17}$$

where $r_0 \approx 0.265\,\text{mm}$ is derived from the condition $G''' = 0$, with

$$G''' = -\frac{\kappa^3}{8D^{3\kappa}} r^{\kappa-3} \left(r^{2\kappa} - \frac{6D^\kappa(\kappa-1)}{\kappa} r^\kappa + \frac{4D^{2\kappa}(\kappa-1)(\kappa-2)}{\kappa^2}\right) G(r), \tag{18}$$

and the exponent κ is chosen such that charge neutrality is satisfied overall,

$$\int \text{d}y \int \text{d}z (Z_\text{i} n_\text{i} - n_\text{e}) = 2\pi \int_0^\infty \text{d}r\, r (Z_\text{i} n_\text{i} - n_\text{e}) = 0, \qquad \text{with} \quad Z_\text{i} \equiv \frac{q_\text{i}}{e}, \tag{19}$$

which means that the electron density would become perfectly flat if the electrons comprising the hump around $r \approx 0.4\,\text{mm}$ in Supplementary Fig. 8(d) were used to fill the cavity in the region $r \lesssim 0.33$. The electric field is given by Gauss' law in cylinder coordinates,

$$\frac{\epsilon_0}{e} \nabla \cdot \boldsymbol{E} = Z_\text{i} n_\text{i}(r) - n_\text{e}(r) \qquad \Rightarrow \qquad \frac{1}{r}(r\hat{E}_r)' = \begin{cases} 1 & : \quad r < r_0, \\ y(r)/y_0 = G''(r)/y_0 & : \quad r \geq r_0, \end{cases} \qquad \text{with} \quad \hat{E}_r = \frac{\epsilon_0 E_r}{e n_\text{e0}}. \tag{20}$$

The solution in the region $r \geq r_0$ is

$$y_0 \hat{E}_r(r \geq r_0) - y_0 \hat{E}_{r0} = \frac{1}{r}\int_{r_0}^{r} \text{d}\tilde{r}\,\tilde{r} G'' = \frac{1}{r}[\tilde{r}G']_{r_0}^{r} - \frac{1}{r}\int_{r_0}^{r} \text{d}\tilde{r}\, G' = -\frac{G(r)}{r}\left(\frac{\kappa r^\kappa}{2D^\kappa} + 1\right) + \frac{G(r_0)}{r}\left(\frac{\kappa r_0^\kappa}{2D^\kappa} + 1\right) \equiv F(r), \tag{21}$$

with $\hat{E}_{r0} \equiv \hat{E}_r(r_0)$. After matching the solutions across r_0, we obtain the following expression for the radial electric field:

$$\frac{\epsilon_0}{e n_\text{e0}} E_r(r) = \begin{cases} r/2 & : \quad r < r_0, \\ r_0/2 + F(r)/y_0 & : \quad r \geq r_0. \end{cases} \tag{22}$$

This setup was implemented in an `input.deck` file for the code `EPOCH`,[49] and Supplementary Fig. 9 shows the results of three simulations performed with (a)–(d) immobile ions, (e)–(h) hydrogen (protons), and (i)–(l) deuterium.

All simulations begin with oscillations in the density of the magnetized electrons with a period of about 13 ps. The spectral analysis of the associated electric and magnetic fluctuations in Supplementary Fig. 10 shows a corresponding peak around $f = 80\,\text{GHz}$, which is near the frequency $\omega_\text{UH} \approx 2\pi \times 74.6\,\text{GHz}$ of an upper-hybrid wave given by Eq. (1) for unperturbed plasma parameters. The reason for the $\approx 5\,\text{GHz}$ discrepancy is not known. It may be connected with the acceleration of electrons, which changes the dispersion relation[52] as in Eq. (8). The origins and values of the other peaks also remain to be

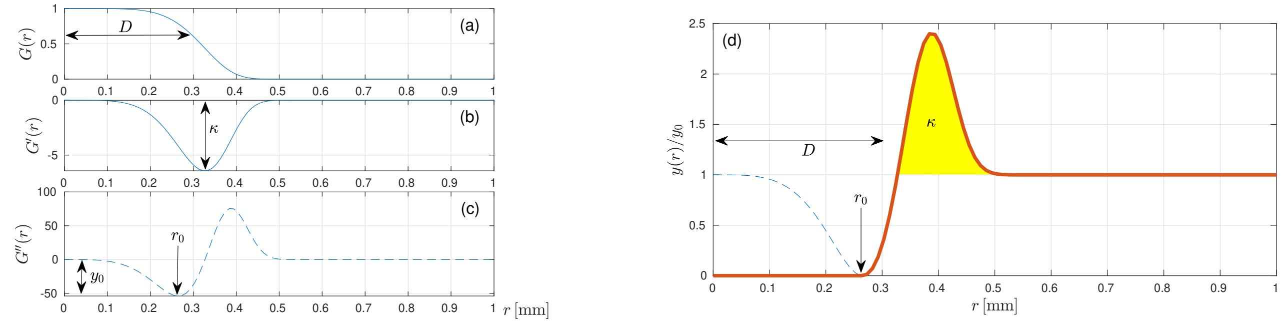

Supplementary Fig 8. Model of the initial electron density profile $n_e(r)$ in Eq. (15a) with $r^2 = y^2 + z^2$ for the simulations in Supplementary Figs. 9 and 10.

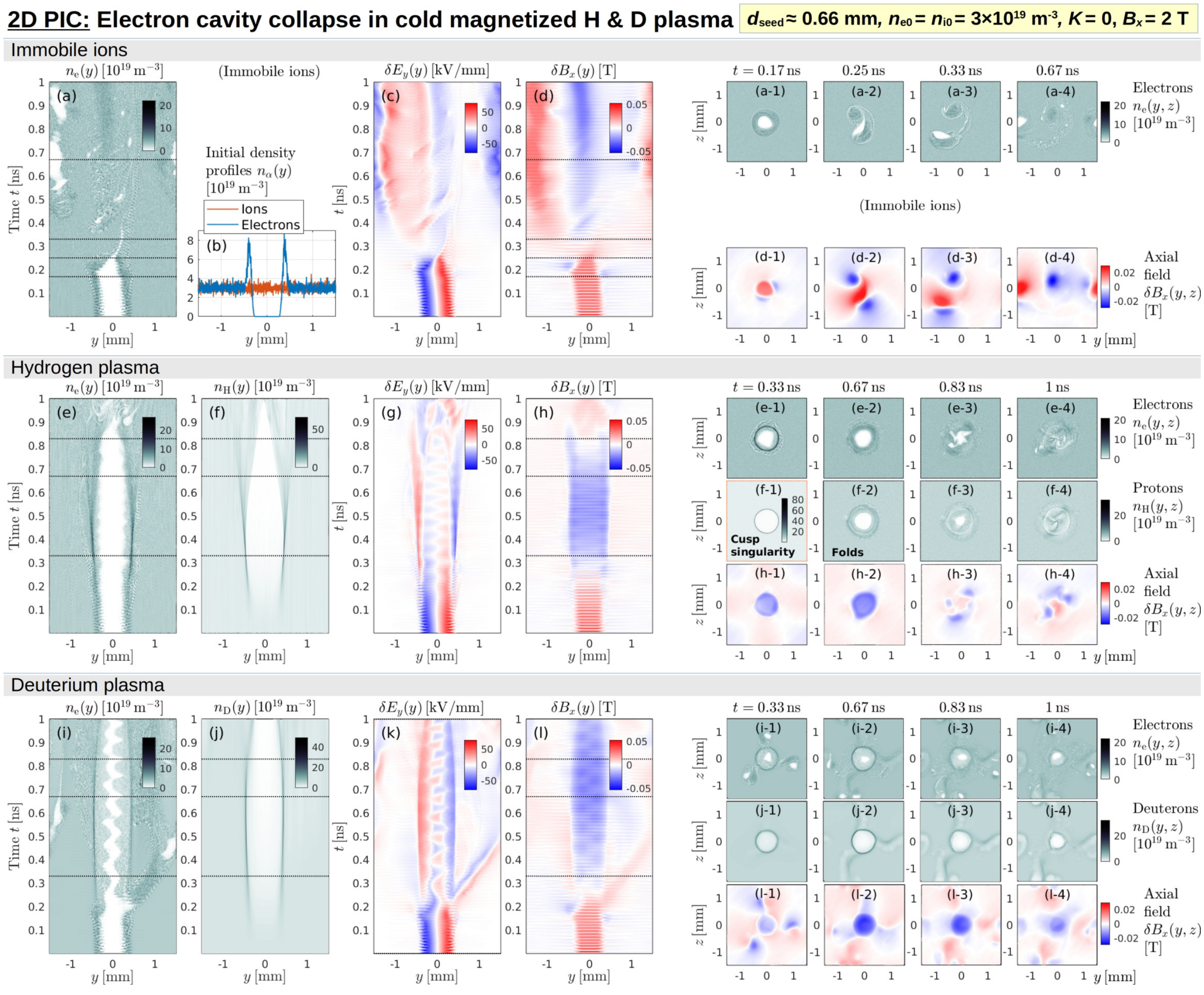

Supplementary Fig 9. 2D PIC simulations showing the evolution of a magnetized cold plasma initialized with uniformly distributed ions and an electron cavity with a diameter of $d_{seed} \approx 0.66$ mm as shown in Supplementary Fig. 8(d). The initial profiles with noise are shown in panel (b). The temporal evolution of the density profiles $n_\alpha(y)$ of electrons ($\alpha = \mathrm{e}$) and ions ($\alpha = \mathrm{H,D}$), and the electromagnetic perturbations $\delta E_y(y)$ and $\delta B_x(y)$ are arranged column-wise in the left part of the figure. The right part shows a series of four snapshots of $n_\alpha(y,z)$ and $\delta B_x(y,z)$ taken at the times indicated by dotted lines on the left. Panels (a)–(d) show results obtained with immobile ions, panels (e)–(h) for hydrogen plasma ($\alpha = \mathrm{H}$), and (i)–(l) for deuterium ($\alpha = \mathrm{D}$). Compared to the hot plasma simulations in Figs. 5–7 of the main paper, the present EPOCH runs used fewer particles per cell ($N_{ppc} = 4$, since the plasma is cold) and a smaller simulation box ($L_y = L_z = 3$ mm, since there is no thermal gyration and since we simulate a shorter time window of only 1 ns), while keeping the same resolution ($N_y = N_z = 1200$). Similar results were obtained with higher resolution ($N_y = N_z = 2400$) and more particles ($N_{ppc} = 16$). See Fig. 2 of the main paper for a comparison between results obtained with cold and 30 keV hot deuterons.

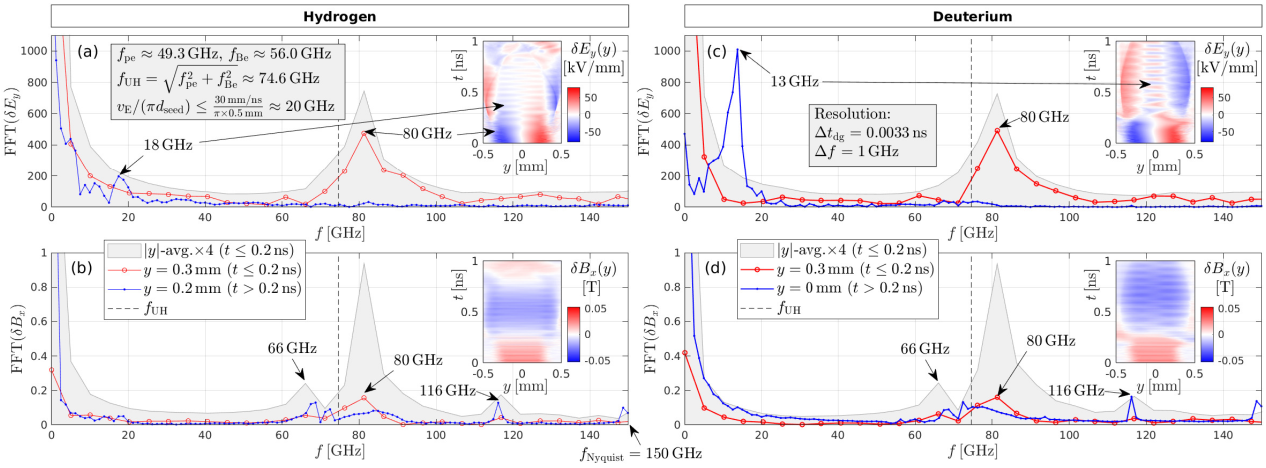

Supplementary Fig 10. Results of spectral analyses of the electromagnetic fluctuation data δE_y and δB_x in panels (g), (h), (k) and (l) of Supplementary Fig. 9, which are partially shown again in the insets in panels (a)–(d). Fast Fourier transforms (FFT) were performed in time at $z = 0$, different y locations, and in different time windows as specified in the legends. The gray shaded areas are spectra averaged over the domain $-0.5\,\text{mm} \leq y \leq 0.5\,\text{mm}$. The upper hybrid frequency $f_{\text{UH}} = 74.6\,\text{GHz}$ of the unperturbed plasma is indicated by a vertical dashed line.

understood, and it is interesting that the electric and magnetic fluctuation spectra differ, except for the peak at 80 GHz. The electric fluctuation activity in the frequency band below 20 GHz seems to be consistent with the electric drift frequency with which electron gyroorbits are expected to revolve around the cavity: $|\delta E_y| \lesssim 60\,\text{kV/mm}$ implies that $v_{\text{E}}/(\pi d_{\text{seed}}) \lesssim 20\,\text{GHz}$. In that low-frequency band, we observe isotope-dependent pulsation frequencies; namely, 18 GHz for hydrogen and 13 GHz for deuterium. These pulsations can be clearly discerned in the raw data (indicated by arrows), but their cause remains to be identified. The magnetic fluctuations exhibit additional isotope-independent high-frequency peaks at 66 GHz and 116 GHz, whose causes also remain to be clarified.

Concerning the temporal evolution of the cavities in Supplementary Fig. 9, the following observations can be made. In the case with immobile ions, Supplementary Fig. 9(a) and the snapshots labeled (a-1)–(a-4) show that the cavity is subject to strong deformations, break-up and millimeter-scale drifts within a few 100 ps. The initial hole+ridge structure breaks up into a spatially separated hole-clump pair, whose magnetization induces complicated magnetic perturbations $\delta B_x(y,z)$ that can be seen in Supplementary Fig. 9(d-1)–(d-4).

When the ions are also evolved, their dynamics begin with a Coulomb explosion that can be seen in Supplementary Fig. 9(f) and (j). Interestingly, the ion velocity distribution turns out to be such that their density profile evolves via two folds and, at their joining, a cusp singularity that is reminiscent of the bow wave and cavity wall (the folds) and their joining (the cusp singularity) in the electron distribution behind the laser pulse in Supplementary Fig. 5(a) of Supplementary Sec. 1.5, which is the subject of BISER studies.[36] In the present case, the cusp singularity in the ion density is ring-shaped and exists only for a brief moment. At least for our setup, the cusp appears sharper in hydrogen than in deuterium. It occurs at about $t \approx 0.33\,\text{ns}$ in Supplementary Fig. 9(f), and snapshot (f-1) shows that the ion density reaches a magnitude on the order of $\sim 100 \times 10^{19}\,\text{m}^{-3}$, which is 30 times higher than the ambient ion density.

For both hydrogen and deuterium, the cavity tends to collapse after the initial Coulomb explosion, but Supplementary Fig. 9 also shows some notable differences in the detailed evolution during that phase (that is, during $t \gtrsim 0.2\,\text{ns}$):

- The time scale and magnitude of electron density oscillations seen in panels (e) and (i) differ. These oscillations appear weaker and faster in the case of hydrogen (18 GHz) than for deuterium (13 GHz) as can also be seen in Supplementary Fig. 10.
- The cavity in the hydrogen plasma closes more rapidly within the 1 ns window shown, while the central cavity in the deuterium plasma appears to be longer-lived, although its diameter has shrunk significantly by the end of the simulation.
- While the imploding cavity in hydrogen retains a compact body (albeit with distorted boundary and turbulent internal structure), the cavity in deuterium spontaneously emits a triplet of smaller daughter cavities, that drift away from the mother cavity. The daughter cavities themselves are clearly visible only in the electron density in Supplementary Fig. 9(i-1)–(i-4), but their motion leaves behind distinct tracks in the deuteron distribution in Supplementary Fig. 9(j-2)–(j-4). Interestingly, the tracks themselves consist of regions with reduced and elevated densities.

It may be worth investigating whether the daughter cavities in these 2D simulations with deuterium are related to the solitary micro-cavity waves seen in the 1D simulations in Fig. 4 of the main paper and Supplementary Fig. 7 of Supplementary Sec. 2.1, or to the ion-filled electron cavity in Fig. 7(j,k) of the main paper and Supplementary Fig. 12(i,j) of Supplementary Sec. 3.3.

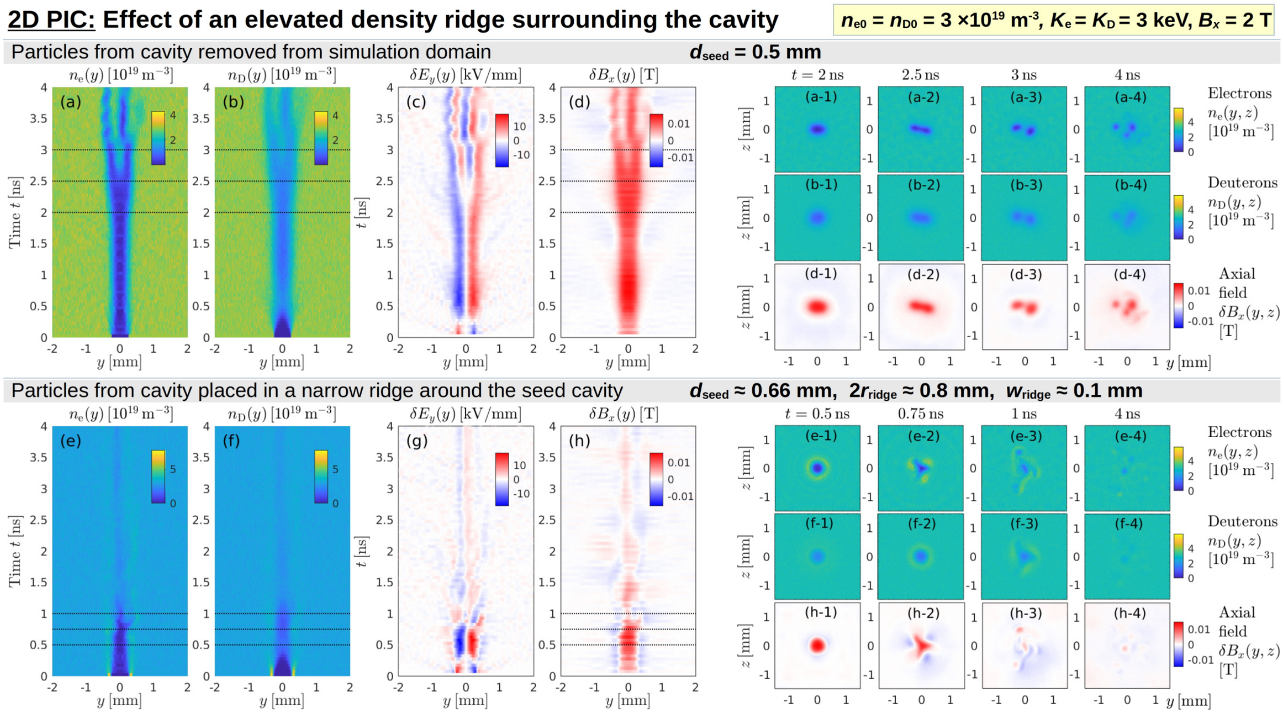

Supplementary Fig 11. Comparison of 2D PIC simulations of a magnetized hot deuterium plasma initialized without (top) and with (bottom) a ridge of elevated density surrounding the central seed cavity. The results for the simple cavity in panels (a)–(d) are the same as in Fig. 7(e)–(h) of the main paper, where the cavity diameter was $d_{seed} = 0.5$ mm. The simulation shown in panels (e)–(h) used the radial density profile shown in Supplementary Fig. 8 for both electrons and ions. In this case, the cavity diameter is about $d_{seed} \approx 2\times 0.33$ mm $= 0.66$ mm. The ridge has a width of about $w_{ridge} \approx 0.1$ mm and peaks at a radius of $r_{ridge} \approx 0.4$ mm from the cavity center. The temporal evolution of the density profiles $n_e(y)$ and $n_D(y)$, and the electromagnetic perturbations $\delta E_y(y)$ and $\delta B_x(y)$ are arranged column-wise in the left part of the figure. The right part shows a series of four snapshots of $n_e(y,z)$, $n_D(y,z)$ and $\delta B_x(y,z)$ taken at the times indicated by horizontal dotted lines on the left. Both simulations were performed using EPOCH with the numerical parameters $L_y = L_z = 4$ mm, $N_y = N_z = 1600$, $N_{ppc} = 40$. We note that some of the pulsations that appear in panels (c), (d), (g) and (h), especially those with periods of about 0.1 ns, are probably aliasing artifacts since these plots were produced with a relatively low snapshot sampling frequency $1/\Delta t_{dg} = 20$ GHz that does not resolve upper hybrid oscillations (74.6 GHz) in these cases (see Supplementary Sec. 3.5 for spectral analyses).

On a more general note, the results in Supplementary Fig. 9 constitute interesting examples that illustrate in what ways the magnetization of the plasma can influence the cavity collapse dynamics in the absence of thermal motion. These results also show that isotope effects should be considered in further studies. Moreover, our impression is that the cavities in the 2D simulations shown in Supplementary Fig. 8 seem to be less robust than in the 3D simulation reported in Ref.[1]. This supports a conjecture we made in the discussion section of the main paper, where we speculated that the simplified 2D setting used in the present study might overestimate the effect of cavity boundary instabilities, for instance, due to overshoots resulting from the artificial initial condition. 3D simulations of a cavity in a magnetized hot plasma are needed to address this issue.

3.2 Sensitivity check in 2D: Effect of elevated density ridge around the seed cavity (Supplementary Fig. 11)

Another test we performed as a variation of the 2D PIC simulations in Figs. 5–7 of the main paper was to initialize the magnetized hot deuterium plasma with electron and ion cavities that are both surrounded by a ridge as in Supplementary Fig. 8. Such a ridge can be expected to form as the result of the ions' Coulomb explosion in a realistic 3D simulation where the cavity is produced by an actual laser pulse. Our mock-up in Supplementary Fig. 8 is meant to mimic the shape of the profile, but our choice of a Maxwellian velocity distribution is not realistic for the ridge ions, which would have been accelerated in a somewhat coherent way during an actual Coulomb explosion. Our cavity models without and with surrounding Maxwellian ridge should therefore be viewed as extreme limits. The simulation results are compared in Supplementary Fig. 11.

One can see at a glance that the presence of the Maxwellian ridge has a devastating effect on the cavity. While the simple cavity in panels (a)–(d) on the left-hand side of Supplementary Fig. 11 is relatively robust for nearly 3 ns, the ridge-rimmed cavity in panels (e)–(h) collapses after about 1 ns. While this difference may appear large here, it does, of course, make no real difference as far as the much longer "valley of death" in Supplementary Fig. 6 of Supplementary Sec. 1.8 is concerned.

The details are nevertheless interesting. One striking feature is that the relaxed electron cavity in the plot of $n_e(y,z)$ in Supplementary Fig. 11(e) consists of a central remnant that has spawned three daughter cavities. The situation is remarkably similar to the one found in Supplementary Fig. 9(i) of Supplementary Sec. 3.1, in spite of the fact that the latter case was

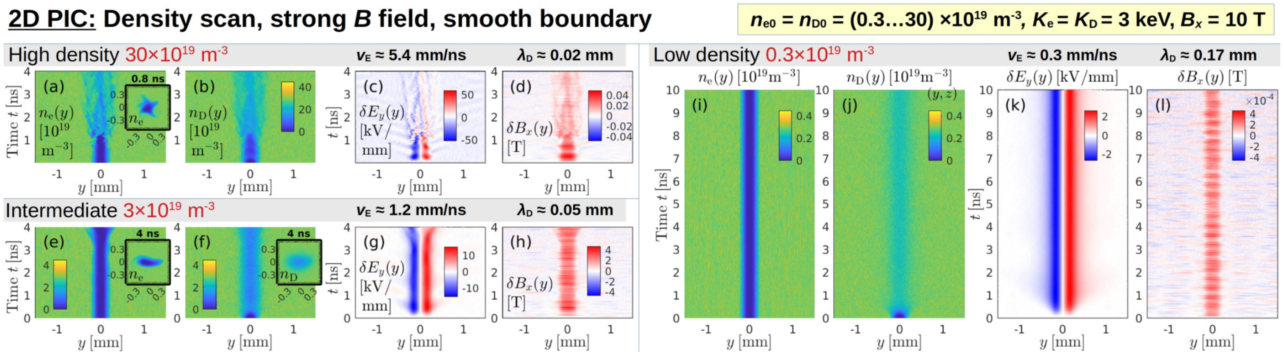

Supplementary Fig 12. Summary of results of 2D PIC simulations using EPOCH[49] with densities in the range $(0.3...30) \times 10^{19}\,\mathrm{m}^{-3}$ and a strong ambient magnetic field $B_x = 10\,\mathrm{T}$. The initial profile of the cavity is chosen to be smooth as in Fig. 7(i) of the main paper. The Debye lengths λ_{De} shown above panels (d), (h) and (l) are the same as in Fig. 7 of the main paper. The values of v_{E} at $t \approx 1\,\mathrm{ns}$ are shown above panels (c), (g) and (k). Pulsations like those seen in panels (h) and (l) are most likely aliasing artifacts since these plots were produced with a relatively low snapshot sampling frequency $1/\Delta t_{\mathrm{dg}} = 20\,\mathrm{GHz}$ that does not resolve the upper hybrid oscillations (here, $\omega_{\mathrm{UH}} > \omega_{\mathrm{Be}} \approx 2\pi \times 280\,\mathrm{GHz}$) that were present in these simulations (see also the spectral analyses in Supplementary Fig. 14 of Supplementary Sec. 3.5 below). The numerical parameters used where $L_y = L_z = 3\,\mathrm{mm}$, $N_y = N_z = 1200$, $N_{\mathrm{ppc}} = 40$.

initialized with a flat ion distribution and with all particles at rest (zero temperature). Hence, this spawning phenomenon seems to be robust with respect to the different initial velocity distributions used in Supplementary Figs. 9 and 11, at least on the short time scale of a few nanoseconds that we inspected here. It would be interesting to check whether this also happens in 3D and whether these daughter cavities can become long-lived, like the solitary cavity waves found in our 1D simulations in Fig. 4 of the main paper and in Supplementary Fig. 7 of Supplementary Sec. 2.1 above, or like the ion-filled electron cavity in Fig. 7(j,k) of the main paper and in Supplementary Fig. 12(i,j) of Supplementary Sec. 3.3 below.

3.3 Sensitivity check in 2D: Density scan in a strong ambient magnetic field (Supplementary Fig. 12)

In Fig. 7 of the main paper, we presented results of a density scan in the presence of a moderate ambient magnetic field with strength $B_x = 2\,\mathrm{T}$. That figure also contained a case initialized with a smoothly bounded cavity. Here, Supplementary Fig. 12 shows the results of another density scan in a 5 times stronger field, $B_x = 10\,\mathrm{T}$. The gyroradius of our 3 keV deuterons is $\rho_{\mathrm{BD}} \approx 1.1\,\mathrm{mm}$, so the orbit diameter is now 4 times larger than the cavity. The electron gyroradius is now only $\rho_{\mathrm{Be}} \approx 0.02\,\mathrm{mm}$. The Debye lengths are the same as in Fig. 7 of the main paper. The $\boldsymbol{E} \times \boldsymbol{B}$ velocities are reduced by a factor 2...5 as one can infer from the values of v_{E} shown above panels (c), (g) and (k) of Supplementary Fig. 12. This reduction of v_{E} has two reasons: the stronger magnetic field and the use of a smooth cavity boundary for the initial condition as in Fig. 7(i) of the main paper.

The overall trends that one can see in the density-dependence with the 10 T field in Supplementary Fig. 12 are similar to those we saw with the 2 T field in Fig. 7 of the main paper. At high density ($30 \times 10^{19}\,\mathrm{m}^{-3}$), the cavity still evolves through all the stages that were outlined in Fig. 5 of the main paper. Although the early reversal of the electric field is not visible in panels (c), (g) and (k) of Supplementary Fig. 12 due to coarse diagnostics, it can be observed at higher diagnostic resolution as we have shown in Fig. 6(o)–(r) of the main paper. The seed cavity in the high-density plasma is still short-lived, lasting not even a nanosecond. The results in Supplementary Fig. 12(a)–(d) obtained with smooth boundary are similar to those in Fig. 6 of the main paper, where the seed cavity boundary was sharp.

At intermediate density ($3 \times 10^{19}\,\mathrm{m}^{-3}$) a comparison between Supplementary Fig. 12(e)–(h) and Fig. 6(e')–(h') of the main paper (both with smoothly bounded seed cavities) indicates that a stronger magnetic field prolonged the life time of the cavity in this case from about 2 ns to nearly 4 ns. At low density ($0.3 \times 10^{19}\,\mathrm{m}^{-3}$), where the Debye length is comparable to the seed cavity radius, $\lambda_{\mathrm{D}} \approx d_{\mathrm{seed}}/2$, the electron cavity is robust and stable during the entire simulation, which lasted 10 ns in Supplementary Fig. 12(i)–(l).

3.4 Sensitivity check in 2D: Spatial resolution and number of particles per cell (Supplementary Fig. 13)

Many convergence tests were performed to ascertain the reliability of the results that are reported in this work. As an example, Supplementary Fig. 13 shows an overview of results from simulations of the high-density case ($30 \times 10^{19}\,\mathrm{m}^{-3}$) with moderate field strength $B_x = 2\,\mathrm{T}$, where we varied the spatial resolution $\Delta_{y,z} = L_{y,z}/N_{y,z}$ and the number of particles per cell, N_{ppc}. We consider these results to be in reasonable agreement. The exact timing and detailed form of the cavity collapse varies slightly, which is to be expected because these dynamics are sensitive to PIC noise and other minor numerical inaccuracies. Similarly small differences can be seen when this case is run with a larger domain size, $L_x = L_y = 12\,\mathrm{mm}$ instead of 4 mm, as was done in Fig. 5 of the main article. In fact, these details vary even when the same code with the same parameters is compiled and run on different computers. This is the reason for the small differences between the results in Supplementary Fig. 13(e)–(h) (run in 2019 on a Linux cluster) and Supplementary Fig. 14(c)–(f) of Supplementary Sec. 3.5 (run in 2023 on JFRS-1).

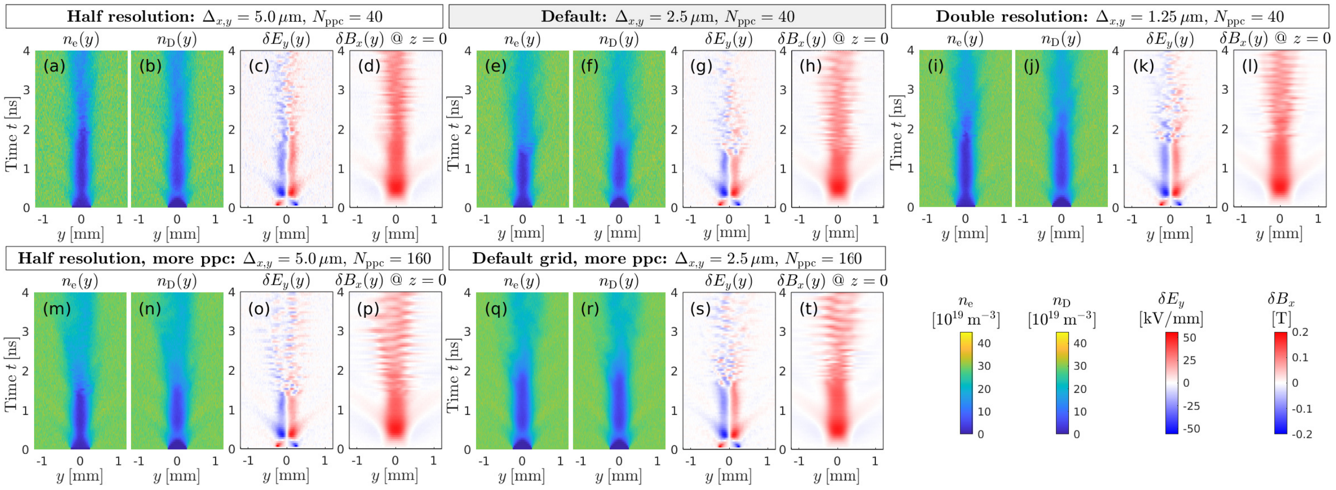

Supplementary Fig 13. Examples of numerical sensitivity tests performed with REMP for the high-density case ($n_0 = 30 \times 10^{19}\,\mathrm{m}^{-3}$) with moderate ambient magnetic field ($B_x = 2\,\mathrm{T}$). The results in panels (e)–(h) are effectively the same as in Supplementary Fig. 14(c)–(f) with default resolution $\Delta_{x,y} = L_{y,z}/N_{y,z} = 2\,\mathrm{mm}/1600 = 2.5\,\mu\mathrm{m} \approx 0.1 \times \lambda_{\mathrm{De}}$, and $N_{\mathrm{ppc}} = 40$ particles per cell. In the upper row, panels (a)–(d) show results obtained with half resolution, and panels (i)–(l) with double resolution. In the lower row, we show results obtained with a 4 times larger number of particles per cell for half resolution (m)–(p) and default resolution (q)–(t).

3.5 Spectral analysis of electric and magnetic fluctuations in hot deuterium plasma (Supplementary Fig. 14)

In Supplementary Fig. 10, we presented results of spectral analyses of the electric and magnetic fluctuations that arose during the evolution of a seed cavity in a cold magnetized plasma consisting of hydrogen and deuterium with high density ($30 \times 10^{19}\,\mathrm{m}^{-3}$). Results of a similar analysis for a 3 keV hot deuterium plasma with densities in the range $(0.3...30) \times 10^{19}\,\mathrm{m}^{-3}$ are summarized in Supplementary Fig. 14. The REMP simulations analyzed here were shown in Figs. 5 and 7 of the main paper, and in Supplementary Fig. 13 of Supplementary Sec. 3.4.

While the cold plasma oscillations in Supplementary Fig. 10 showed a clear peak near the expected upper hybrid frequency $f_{\mathrm{UH}} = \omega_{\mathrm{UH}}/(2\pi) = 74.6\,\mathrm{GHz}$, such a distinct peak can be seen in the hot plasma spectra only in the low-density case in Supplementary Fig. 14(i,j), where $f_{\mathrm{UH}} = 58.1\,\mathrm{GHz}$. That peak becomes very sharp when one excludes the initial transients and performs the FFT only over the time window $t > 2\,\mathrm{ns}$, as the blue curve in Supplementary Fig. 14(i) shows. The intermediate-density case in Supplementary Fig. 14(g,h) shows much activity in the frequency range 50...75 GHz, which is not far from the expected $f_{\mathrm{UH}} = 74.6\,\mathrm{GHz}$. In the high-density case, spectra in Supplementary Fig. 14(a,b) are rather noisy. Only the 190 GHz peak is clearly seen in both the electric (a) and magnetic fluctuation spectra (b), but only during the time window $t > 1.3\,\mathrm{ns}$, where the instability develops. The upper hybrid frequency in the high-density case is $f_{\mathrm{UH}} = 165.7\,\mathrm{GHz}$, but Supplementary Fig. 14(a,b) shows no clear peak nearby. Note that the 9-point average used for the red and blue curves in Supplementary Fig. 14 corresponds to an average over the Debye length $\lambda_{\mathrm{D}} \approx 0.02\,\mathrm{mm} \approx 9\Delta_y$ in the high-density case.

Supplementary Fig. 14 shows various peaks below 20 GHz that may be partly attributed to electric drifts, whose nominal frequencies for seed-cavity scale structures lie in the range $1\,\mathrm{GHz} \lesssim v_{\mathrm{E}}/(\pi d_{\mathrm{seed}}) \lesssim 6\,\mathrm{GHz}$ for the three cases shown in Supplementary Fig. 14. However, the origin of most peaks in Supplementary Fig. 14 remains obscure and we cannot rule out the possibility that some of them are aliasing artifacts.

Further insight may be gained by considering thermal effects. For instance, the Bohm-Gross dispersion relation in Eq. (8) of Supplementary Sec. 1.5 implies that warm plasma frequency shifts are in the range

$$\frac{f_{\mathrm{BG}}}{f_{\mathrm{pe}}} = \frac{\sqrt{3}\lambda_{\mathrm{De}}}{\lambda} \approx \frac{1}{\lambda[\mathrm{mm}]} \times \begin{cases} 0.04 & : \quad \text{high } 30 \times 10^{19}\,\mathrm{m}^{-3}, \\ 0.13 & : \quad \text{intermediate } 3 \times 10^{19}\,\mathrm{m}^{-3}, \\ 0.41 & : \quad \text{low } 0.3 \times 10^{19}\,\mathrm{m}^{-3}. \end{cases} \tag{23}$$

On electron gyroradius scales ($\lambda \sim \rho_{\mathrm{Be}} \approx 0.1\,\mathrm{mm}$) in the high-density case, we may thus expect the frequency $f = f_{\mathrm{pe}} \times (1 + f_{\mathrm{BG}}/f_{\mathrm{pe}})^{1/2} \approx f \times 1.08$ to be shifted by nearly 10%. Adding the gyrofrequency contribution, we may then expect a warm plasma upper hybrid oscillation at 180 GHz, and Supplementary Fig. 14(a,b) shows indeed a clear peak at the nearby frequency of 190 GHz. However, this may also be a coincidence since there are also various other peaks of comparable or larger size at other frequencies. In the intermediate-density case, the nominal Bohm-Gross frequency shift for $\lambda \sim \rho_{\mathrm{Be}}$ is nearly 65%, so the expected warm-plasma upper hybrid oscillation would be around 100 GHz. The magnetic fluctuation spectrum in

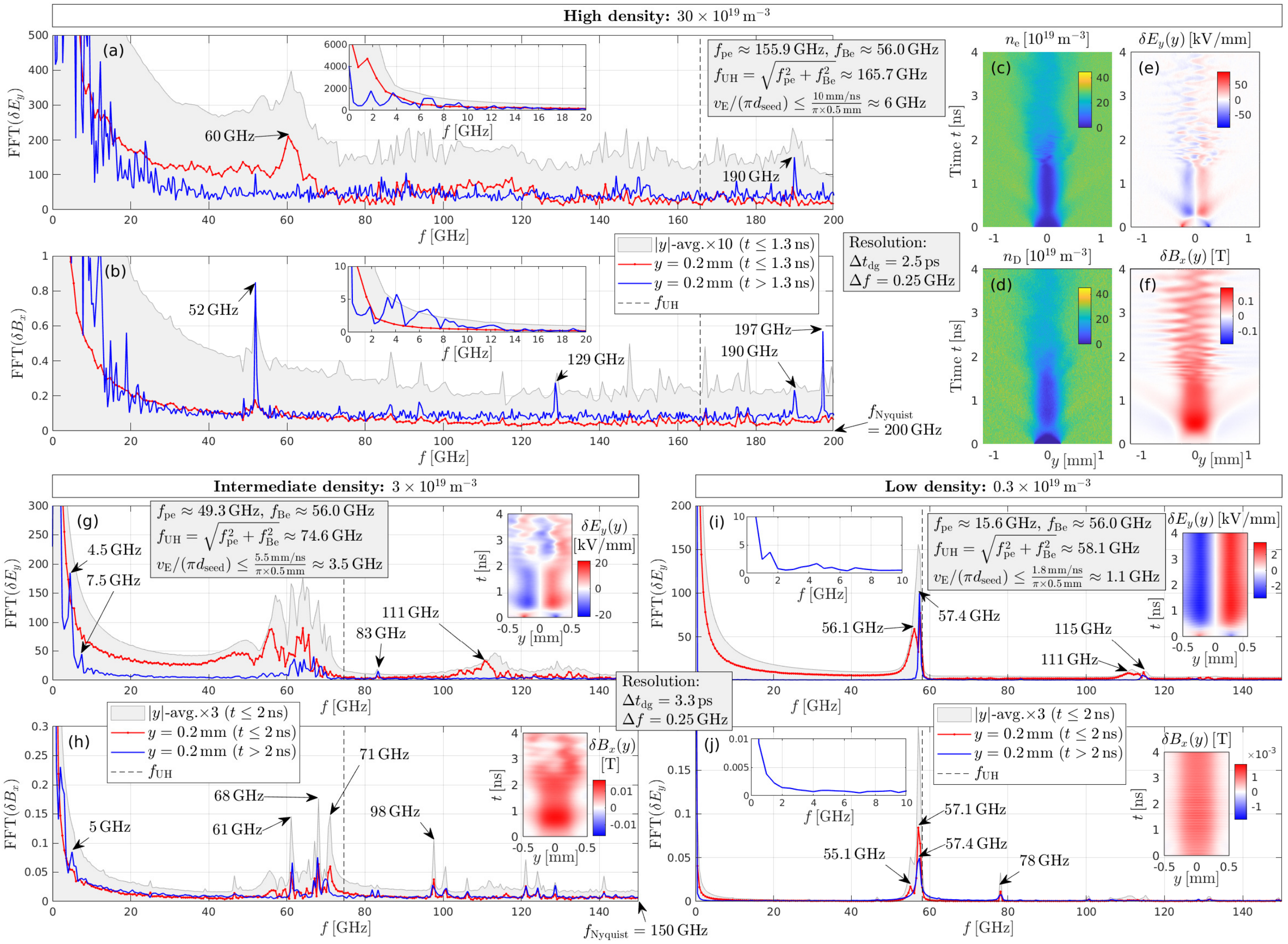

Supplementary Fig 14. Time domain FFT spectral analysis of REMP results from Fig. 7 of the main article and Supplementary Fig. 13(e)–(h) of Supplementary Sec. 3.4. As indicated in the legends, FFTs were performed for time windows before and after a certain time t_0. In the cases with high (a,b) and intermediate densities (g,h), we let $t_0 = 1.3$ ns and 2 ns, which are roughly the respective instability onset times. In the low-density case (i,j), there is no instability and we chose $t_0 = 2$ ns to be half the simulation time. The gray shaded areas are spectra averaged over the domain $-0.5\,\mathrm{mm} \leq y \leq 0.5\,\mathrm{mm}$; that is, twice the size of the seed cavity. The red and blue curves are averaged over 9 spatial samples around $y \approx 0.2$ mm. All data are evaluated at $z = 0$. Panels (a) and (b) for the high-density case are based on δE_y and δB_x data in panels (e) and (f). The case in panels (c)–(f) is the same as in Fig. 5(b)–(e) of the main article, except that the latter used a larger domain size ($L_{x,y} = 12$ mm instead of 4 mm) since it was run for a longer time (10 ns). This is the reason for the small differences in their detailed evolution. Panels (g)–(j) for the intermediate- and low-density cases are based on the data in the main article's Figs. 7(g,h) and 7(l,m), respectively, portions of which are reproduced in the insets. Vertical dashed lines indicate the upper hybrid frequencies f_UH.

Supplementary Fig. 14(h) does have some peaks around that frequency and it is possible that the electric fluctuation activity around (110...120) GHz in Supplementary Fig. 14(g) may also be related to warm-plasma upper-hybrid oscillations, but all this is mere speculation. Moreover, the validity of Eq. (23) is questionable since $\lambda_\mathrm{De} \sim \rho_\mathrm{Be}$. In the low-density case, the Bohm-Gross dispersion relation is invalid at electron gyration scales because $\lambda_\mathrm{De} > \rho_\mathrm{Be}$. In this case, however, Supplementary Fig. 14(i,j) shows a clear peak near the theoretically predicted cold plasma upper hybrid frequency, $f_\mathrm{UH} = 58.1$ GHz, so thermal effects do not seem to have a strong influence here.

Interestingly, the dominant frequency peak in Supplementary Fig. 14(i,j) is slightly down-shifted relative to f_UH, which disagrees even qualitatively with the trend predicted by the Bohm-Gross dispersion relation (8). Perhaps such down-shifts also occur in the intermediate- and high-density cases, so our discussion based on Eq. (23) in the previous paragraph may be misguided. With the possibility of reversed trends, it is then possible that the warm plasma oscillations are the cause of activity seen around 50...75 GHz in Supplementary Fig. 14(g,h) in the intermediate-density case. In the high-density case it may be spread out even further and cause the broad-band noise seen in Supplementary Fig. 14(a,b). It must also be kept in mind that fluctuations with frequencies above the Nyquist frequency of our diagnostics — indicated by arrows below panels (b) and (h) in Supplementary Fig. 14 — will cause aliasing artifacts.

References

1. Bierwage, A., Esirkepov, T., Koga, J. & Pirozhkov, A. Similarity of magnetized plasma wake channels behind relativistic laser pulses with different wavelengths. *Comp. Phys. Comm* **244**, 49 (2019).
2. Bierwage, A. *et al.* Effect of relativistically intense laser pulses on magnetically confined fusion plasmas. In *Proceedings of the 46th EPS Conference on Plasma Physics*, Poster P1.1091 (Milan, Italy, July 8-12, 2019). `http://ocs.ciemat.es/EPS2019PAP/pdf/P1.1091.pdf`.
3. de Vries, P. & Gribov, Y. ITER breakdown and plasma initiation revisited. *Nucl. Fusion* **59**, 096043 (2019).
4. Sugiyama, S., Shinya, K., Utoh, H., Aiba, N. & Sakamoto, Y. A study on ohmic plasma initiation for JA DEMO. *Fusion Eng. Design* **172**, 112779 (2021).
5. Zohm, H. Edge localized modes (ELMs). *Plasma Phys. Control. Fusion* **38**, 105 (1996).
6. Connor, J. A review of models for ELMs. *Plasma Phys. Control. Fusion* **40**, 191 (1998).
7. Connor, J. Edge-localized modes – physics and theory. *Plasma Phys. Control. Fusion* **40**, 531 (1998).
8. Wolf, R. Internal transport barriers in tokamak plasmas. *Plasma Phys. Control. Fusion* **45**, R1 (2003).
9. Wagner, F. A quarter-century of H-mode studies. *Plasma Phys. Control. Fusion* **49**, B1 (2007).
10. Wagner, F. *et al.* Development of an edge transport barrier at the H-mode transition of ASDEX. *Phys. Rev. Lett.* **53**, 1453 (1984).
11. ITER Organization. ITER Research Plan within the Staged Approach (Level III - Provisional Version). Permalink: `https://www.iter.org/technical-reports?id=9` (2018). Reference: ITR-18-003. Accessed 21 February 2023.
12. Burrell, K. *et al.* Quiescent double barrier high-confinement mode plasmas in the DIII-D tokamak. *Phys. Plasmas* **8**, 2153 (2001).
13. Evans, T. *et al.* Edge stability and transport control with resonant magnetic perturbations in collisionless tokamak plasmas. *Nature Phys.* **2**, 419 (2006).
14. Loarte, A. Chaos cuts ELMs down to size. *Nature Phys.* **2**, 369 (2006).
15. Lang, P. *et al.* ELM control strategies and tools: status and potential for ITER. *Nucl. Fusion* **53**, 043004 (2013).
16. Park, J.-K. *et al.* 3D field phase-space control in tokamak plasmas. *Nature Phys.* **14**, 1223 (2018).
17. Garcia, J. *et al.* New H-mode regimes with small ELMs and high thermal confinement in the Joint European Torus. *Phys. Plasmas* **29**, 032505 (2022).
18. Harrer, C. *et al.* Quasicontinuous exhaust scenario for a fusion reactor: The renaissance of small edge localized modes. *Phys. Rev. Lett.* **129**, 165001 (2022).
19. Horacek, J. *et al.* Novel concept suppressing plasma heat pulses in a tokamak by fast divertor sweeping. *Sci. Rep.* **12**, 17013 (2022).
20. Zweben, S. *et al.* Edge turbulence measurements in toroidal fusion devices. *Plasma Phys. Control. Fusion* **49**, S1 (2007).
21. Han, W. *et al.* Tracking blobs in the turbulent edge plasma of a tokamak fusion device. *Sci. Rep.* **12**, 18142 (2022).
22. Yun, G. *et al.* Two-dimensional visualization of growth and burst of the edge-localized filaments in KSTAR H-Mode plasmas. *Phys. Rev. Lett.* **107**, 045004 (2011).
23. Lee, J., Seo, P., Bak, J. & Yun, G. Solitary perturbations in the steep boundary of magnetized toroidal plasma. *Sci. Rep.* **7**, 45075 (2017).
24. Lee, J., Seo, P., Bak, J. & Yun, G. A machine learning approach to identify the universality of solitary perturbations accompanying boundary bursts in magnetized toroidal plasmas. *Sci. Rep.* **21**, 3663 (2021).
25. Wenninger, R. *et al.* Solitary magnetic perturbations at the ELM onset. *Nucl. Fusion* **52**, 114025 (2012).
26. Yan, N. *et al.* Langmuir-magnetic probe measurements of ELMs and dithering cycles in the EAST tokamak. *Plasma Phys. Control. Fusion* **56**, 095023 (2014).
27. Schmid, P. Nonmodal stability theory. *Annu. Rev. Fluid Mech.* **39**, 129 (2007).
28. Leconte, M., Jeon, Y. & Yun, G. S. Ginzburg-Landau model in a finite shear-layer and onset of transport barrier nonlinear oscillations: A paradigm for Type-III ELMs. *Contrib. Plasma Phys.* **56**, 736 (2016).

29. Oh, Y., Hwang, H., Leconte, M., Kim, M. & Yun, G. Effect of time-varying flow-shear on the nonlinear stability of the boundary of magnetized toroidal plasmas. *AIP Adv.* **8**, 025224 (2018).

30. Leconte, M. Phase-mixing v.s. phase synchronization in the dynamics of flow-shear induced edge transport barrier. *Phys. Plasmas* **26**, 072302 (2019).

31. Lee, M., Yun, G. & Ji, J.-Y. Nonlinear harmonics coupled by parallel wave propagations in a time-dependent plasma flow. *Plasma Phys. Control. Fusion* **64**, 055005 (2022).

32. Lee, M., Yun, G. & Ji, J.-Y. Dispersion relation and instability for an anisotropic nonuniform flowing plasma. *Plasma Phys. Control. Fusion* **64**, 125003 (2022).

33. Thatipamula, S. *et al.* Dynamic spectra of radio frequency bursts associated with edge-localized modes. *Plasma Phys. Control. Fusion* **58**, 065003 (2016).

34. Kim, M. *et al.* Distinct stages of radio frequency emission at the onset of pedestal collapse in KSTAR H-mode plasmas. *Nucl. Fusion* **58**, 096034 (2018).

35. Kim, M. *et al.* Intense whistler-frequency emissions at the pedestal collapse in KSTAR H-mode plasmas. *Nucl. Fusion* **60**, 126021 (2020).

36. Pirozhkov, A. *et al.* Burst intensification by singularity emitting radiation in multi-stream flows. *Sci. Rep.* **7**, 17968 (2017).

37. Tajima, T. & Dawson, J. Laser electron accelerator. *Phys. Rev. Lett.* **43**, 267 (1979).

38. Pirozhkov, A. *et al.* Approaching the diffraction-limited, bandwidth-limited Petawatt. *Optics Express* **25**, 20486 (2017).

39. Kando, M. *et al.* Research on laser acceleration and coherent x-ray generation using J-KAREN-P laser. *Springer Proc. Phys.* **202**, 135 (2018).

40. Kiriyama, H. *et al.* Status and progress of the J-KAREN-P high intensity laser system at QST. *High Energy Density Phys.* **36**, 100771 (2020).

41. Bulanov, S. *et al.* Nonlinear plasma wave in magnetized plasmas. *Phys. Plasmas* **20**, 083113 (2013).

42. Haberberger, D., Tochitsky, S. & Joshi, C. Fifteen terawatt picosecond CO_2 laser system. *OPT. EXPRESS* **18**, 17865 (2010).

43. Polyanskiy, M. *et al.* High-peak-power long-wave infrared lasers with CO_2 amplifiers. *Photonics* **8**, 101 (2021).

44. Militsyn, B., von Helden, G., Meijer, G. & van der Meer, A. FELICE—the free electron laser for intra-cavity experiments. *Nucl. Instr. Methods Phys. Res. A: Accelerators, Spectrometers, Detectors and Associated Equipment* **507**, 494 (2003).

45. FELIX Laboratory. URL: `https://www.ru.nl/felix/facility/overview-specification/`. Accessed 24 January 2023.

46. Rauer, P. *A Proof-Of-Principle Cavity-Based X-Ray Free-Electron-Laser Demonstrator at the European XFEL*. Ph.D. thesis, Fachbereich Physik, der Universität Hamburg (2022). DESY Publications Database. URL: `https://bib-pubdb1.desy.de/record/478856/`.

47. Pogorelsky, I., Babzien, M., Ben-Zvi, I., Skaritka, J. & Polyanskiy, M. BESTIA – The next generation ultra-fast CO_2 laser for advanced accelerator research. *Nucl. Instr. Meth. Phys. Res. A* **829**, 432 (2016).

48. Polyanskiy, M., Babzien, M. & Pogorelsky, I. BESTIA (Brookhaven Experimental Supra-Terawatt Infrared at ATF) laser: A status report. *AIP Conf. Proc.* **1812**, 110007 (2017).

49. Arber, T. *et al.* Contemporary particle-in-cell approach to laser-plasma modelling. *Plasma Phys. Control. Fusion* **57**, 113001 (2015).

50. Johnson, L., Gordon, D., Palastro, J. & Hafizi, B. Backward Raman amplification in the long-wavelength infrared. *Phys. Plasmas* **24**, 033107 (2017).

51. Kruer, W. *The Physics of Laser Plasma Interactions*. Frontiers in Physics (CRC Press, Boca Raton, 2001), 1st edn.

52. Silveira, F., Benetti, M. & Caldas, I. Suprathermal corrections to Bohm–Gross dispersion. *Phys. Plasmas* **29**, 052113 (2022).

53. Bohm, D. & Gross, E. Theory of plasma oscillations. A. Origin of medium-like behavior. *Phys. Rev.* **75**, 1851 (1949).

54. Wesson, J. *Tokamaks* (Oxford University Press, New York, 2011), 4th edn.

55. Kamiya, K. *et al.* Modifications to the edge radial electric field by angular momentum injection in JT-60U and their implication for pedestal transport. *Nucl. Fusion* **52**, 114010 (2012).

56. Viezzer, E. *et al.* High-accuracy characterization of the edge radial electric field at ASDEX Upgrade. *Nucl. Fusion* **53**, 053005 (2013).

57. White, R. B. *The Theory of Toroidally Confined Plasmas* (Imperial College Press, London, 2014), 3rd edn.

58. Bierwage, A. *et al.* Energy-selective confinement of fusion-born alpha particles during internal relaxations in a tokamak plasma. *Nature Commun.* **13**, 3941 (2022).

59. Esirkepov, T. Exact charge conservation scheme for particle-in-cell simulation with an arbitrary form-factor. *Comp. Phys. Comm.* **135**, 144 (2001).

60. Askaryan, G. Effect of the gradient of a strong electromagnetic ray on electrons and atoms. *Zhur. Eksptl'. i Teoret. Fiz.* **42**, 1567 (1962).

61. Litvak, A. Finite-amplitude wave beams in a magnetoactive plasma. *Sov. Phys. JETP* **30**, 344 (1970).

62. Sun, G.-Z., Ott, E., Lee, Y. & Guzdar, P. Self-focusing of short intense pulses in plasmas. *Phys. Fluids* **30**, 526 (1987).
